# Adverse effects of removable orthodontic aligners: A systematic review with single-arm meta-analysis

**DOI:** 10.1371/journal.pone.0350741

**Published:** 2026-07-20

**Authors:** Cintia Ronchi Lemos, Marianella Aguilar Ventura Fadel, Helena Polmann, Júlia Meller Dias de Oliveira, Patrícia Pauletto, Cristine Miron Stefani, Carlos Flores-Mir, Graziela De Luca Canto

**Affiliations:** 1 Brazilian Centre for Evidence-Based Research (COBE), Department of Dentistry, Federal University of Santa Catarina (UFSC), Florianópolis, Brazil; 2 School of Dentistry, Universidad De Las Américas (UDLA), Quito, Ecuador; 3 Brazilian Center for Evidence-Based Research (COBE), University of Brasilia, Brasilia, Brazil; 4 Mike Petryk School of Dentistry, University of Alberta, Edmonton, Alberta, Canada; 5 Postgraduate Program in Evidence-Based Health, Federal University of São Paulo, São Paulo, Brazil; Justus Liebig University Giessen, GERMANY

## Abstract

**Background:**

Removable orthodontic aligners are widely used due to their aesthetic appeal, removability, and perceived comfort. However, evidence regarding their potential adverse effects remains limited.

**Objective:**

To identify and synthesize the adverse effects associated with removable orthodontic aligner therapy.

**Methods:**

This systematic review with single-arm meta-analysis evaluated adverse effects related to removable orthodontic aligner therapy. Seven electronic databases (Cochrane, Embase, LILACS, Livivo, MEDLINE, Scopus, and Web of Science) and gray literature sources (ProQuest Dissertations and Google Scholar) were searched without language or date restrictions. Two calibrated reviewers independently selected studies. Risk of bias was assessed using RoB 2 for randomized trials and ROBINS-I for non-randomized studies. Due to clinical and methodological heterogeneity, most outcomes were synthesized narratively; meta-analyses were conducted only for pain/discomfort, apical root resorption, and plaque.

**Results:**

Thirty-four studies met the inclusion criteria, including 10 randomized controlled trials, 6 non-randomized clinical studies, and 18 cohort studies. Randomized trials were predominantly at low risk of bias, whereas most non-randomized and cohort studies were at moderate to high risk. Pain and apical root resorption were the most frequently reported adverse effects. Pain peaked within 24 hours, decreased by day 3, and was minimal by 1 week. Apical root resorption was generally small, with a mean linear loss of −0.33 mm (95% CI −0.55 to −0.11) and a volumetric loss of −4.37 mm³ (95% CI −5.51 to −3.24). Findings on plaque at 3 months were inconclusive. Other reported outcomes included periodontal changes, enamel demineralization, white spot lesions, speech alterations, halitosis, open gingival embrasures, temporomandibular symptoms, and awake bruxism.

**Conclusion:**

Short-term pain and minor apical root resorption appear to be the most frequently reported adverse effects of removable orthodontic aligners, although the certainty of evidence varies across outcomes. Speech alterations, discomfort, and white spot lesions have also been reported.

## Introduction

Growing demand for more aesthetic and comfortable orthodontic care has led patients and clinicians to seek alternatives to fixed appliances. Removable orthodontic aligners (clear aligners) have become increasingly popular because they are virtually invisible, are perceived as more comfortable, and are easier to maintain than traditional metal braces [1–3].

However, although orthodontic treatment corrects malocclusions and restores function, it may also result in adverse effects. Enamel demineralization can occur, leading to white spot lesions (WSLs) and an increased risk of caries [4]. Apical root resorption (ARR), associated with the magnitude of applied forces as well as genetic, biological, and systemic factors, remains common [5, 6], and inadequate oral hygiene during treatment may lead to gingival inflammation and attachment loss [7].

With fixed appliances, patients frequently experience discomfort and pain following appliance adjustments, particularly during the early phases of treatment [8]. Allergic reactions to nickel in metal alloys may cause oral irritation or contact dermatitis [9]. Concerns regarding metal ion release during orthodontic therapy have also been raised, although their clinical significance remains unclear [10]. Previous studies suggest that patients treated with clear aligners may experience slightly lower pain intensity and greater comfort, possibly due to reduced soft tissue irritation and the removability of the appliance [11]. Randomized trials comparing aligners and fixed appliances have examined patient-reported outcomes and provide context for interpreting harms reported in single-arm aligner studies [12].

Despite these advantages, clear aligners may also be associated with several adverse effects. Reported outcomes include transient pain and discomfort during the early stages of treatment, ARR, and periodontal changes such as variations in alveolar bone height or thickness and gingival inflammation [13, 14, 15–17]. Dental alterations, including enamel demineralization, white spot lesions, and caries, have also been described during aligner therapy [18–21]. In addition, patient-reported outcomes such as speech alterations, halitosis, open gingival embrasures, and temporomandibular symptoms have been documented in clinical studies evaluating clear aligner treatment [22–24]. However, the magnitude, frequency, and clinical relevance of these adverse outcomes remain uncertain, as available studies vary substantially in design, follow-up duration, outcome definitions, and measurement methods.

As the use of clear aligners continues to expand, it is increasingly important to investigate their potential adverse effects. Recent systematic reviews (SRs) [25–27] have examined specific aspects of aligner therapy, including speech alterations, apical root resorption, and oral health outcomes. However, these reviews have generally focused on isolated outcomes rather than providing a comprehensive synthesis of the full range of adverse effects associated with aligner therapy.

An updated and inclusive synthesis should address the full spectrum of potential adverse effects, including those related to periodontal health, speech, soft tissue responses, biocompatibility, systemic implications, and dental changes. Given the substantial variability in outcome definitions, measurement instruments, and follow-up periods across studies, interpreting the overall burden and clinical relevance of these effects remains challenging.

Therefore, this systematic review aims to provide a comprehensive, evidence-based synthesis of adverse effects associated with clear aligners. By focusing exclusively on adverse outcomes reported in patients treated with aligners, this review seeks to support informed clinical decision-making and the development of strategies to minimize risks while optimizing treatment safety and efficacy.

## Materials and methods

### Protocol and registration

The protocol of this SR was developed in accordance with the Preferred Reporting Items for Systematic Reviews and Meta-Analyses Protocols (PRISMA-P) and has been registered in the International Prospective Register of Systematic Reviews (PROSPERO) under CRD42023458491. The protocol has also been published previously [28]. As this study is a systematic review based exclusively on data from previously published studies, no new data were collected from human participants, no individual participant data were accessed, and no identifiable personal information was used. Therefore, ethics committee approval and informed consent were not required. This SR was reported in accordance with the Preferred Reporting Items for Systematic Reviews and Meta-Analyses (PRISMA) 2020 statement [29] (S1 Checklist – PRISMA checklist) and the Synthesis Without Meta-analysis (SWiM) guidelines (S2 Checklist – SWiM checklist) [30].

### Research question

**Fig 1:**
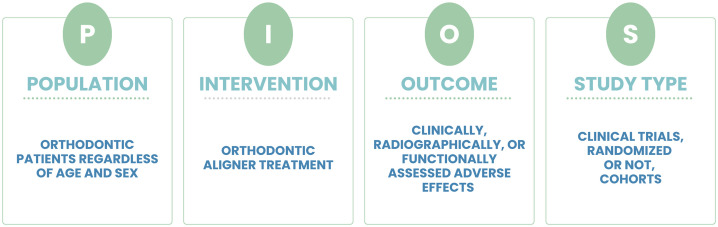
PIOS-Based Structuring of the Research Question.

This SR aimed to address the following question: “Among orthodontic patients wearing orthodontic removable aligners, what are the potential adverse effects?”. The research question was structured according to the PIOS framework (Population, Intervention, Outcomes, and Study Design) (Fig 1).

### Eligibility criteria

The review included cohort studies and clinical trials, whether randomized or not, involving orthodontic patients of any age or sex. Eligible studies evaluated clear aligners and reported adverse effects during or after treatment. For studies with multiple arms, only data from the group treated with clear aligners were included for analysis. No language or publication date restrictions were applied. Studies were excluded for the following reasons:

1. Ineligible population;
2. Intervention not limited exclusively clear aligners (e.g., hybrid with fixed/anchorage, other removable devices) or where an aligner-only sample could not be isolated.
3. Ineligible outcomes (e.g., studies reporting only treatment effectiveness or patient satisfaction, without assessing adverse effects such as ARR, periodontal changes, pain, or speech impairment);
4. Non-primary research and ineligible designs: opinion pieces, conference materials, technical articles, clinical guidelines, reviews, descriptive studies, and case reports.

As pre-specified, studies without available full text or with missing data were to be excluded after three attempts to contact the corresponding authors by email. Full texts were obtained for all eligible studies.

### Information sources

Searches were conducted in Cochrane (CENTRAL), Embase, Latin American and Caribbean Health Sciences Literature (LILACS, via BVS [from Portuguese: Biblioteca Virtual em Saúde]), Livivo, MEDLINE (via PubMed), Scopus, and Web of Science. Grey literature sources were also searched, including Google Scholar and ProQuest Dissertations & Theses Citation Index, to reduce potential publication bias and identify relevant studies not indexed in traditional bibliographic databases. This approach is particularly important in SR of adverse effects, where harms may be incompletely reported in published literature. Reference lists of included studies and experts on the topic were also consulted. The search was conducted by the first author on May 4, 2025.

### Search strategy and study selection

A comprehensive search strategy, developed in collaboration with a health sciences librarian, was applied to seven key databases (S3 Table – Search strategy), with syntax adapted for each platform. References were managed in EndNote-Web™ (Clarivate, USA) to remove duplicates before import into Rayyan® (Qatar Computing Research Institute) for further deduplication and selection [31, 32]. Two reviewers (CRL and MAVF) independently screened titles and abstracts in phase 1 and full texts in phase 2 according to predefined eligibility criteria. Prior to formal screening, the reviewers conducted a calibration exercise using a pilot sample of studies to ensure consistent interpretation of the eligibility criteria. Discrepancies were discussed until consensus was reached and the screening criteria were refined when necessary. During the selection process, disagreements were resolved through discussion and, when necessary, consultation with a third reviewer (HP). Studies published in non-Latin scripts were translated using the Google extension Sider AI [33], and fully rendered into English via ChatGPT [34], including figures and tables. Studies published online ahead of print or available as early online versions at the time of the search were considered eligible and included when they met the predefined criteria. Publication year was recorded according to the final journal assignment when available.

### Data items and collection

Two reviewers (CRL and MAVF) independently extracted data into Excel® (Microsoft, USA), with discrepancies resolved through discussion or consultation with a third reviewer (HP). When necessary, data transformations were performed, including conversion of median to mean [35, 36], and extraction of data from graphical representations using WebPlotDigitizer (https://automeris.io/WebPlotDigitizer) [37]. A summary table presents key study characteristics, including authors, country, year, sample size, intervention type, treatment duration, measurement methods, outcomes, adverse effects, frequency, and follow-up. Additional comprehensive tables were developed to organize and detail all extracted data.

### Risk of bias/quality assessment in individual studies

The risk of bias of the included studies was assessed using the RoB 2 tool [38] for randomized controlled trials (RCTs) and the ROBINS-I tool [39] for non-randomized studies (NRS) and cohorts. ChatGPT (OpenAI) [34] was used solely to assist in identifying relevant text excerpts to inform domain-specific judgments through predefined prompts (S4 - AIs Risk of Bias Prompts). All risk-of-bias assessments and data extraction were performed independently by two reviewers (CRL and MAVF), with AI outputs used only as supportive material and critically appraised before any decision-making. Disagreements were resolved by consultation with a third reviewer (HP). Prior to formal assessment, a calibration exercise was conducted using one study from each design category to ensure consistent interpretation of the RoB 2 and ROBINS-I domains. Results were reported according to each tool’s guidance, and visualizations were generated using robvis [40].

### Synthesis methods

Given the absence of comparison groups, meta-analyses of single-arm means (and change scores, when applicable) were conducted only when outcomes were consistently defined, measured, and reported across studies. Quantitative synthesis was restricted to outcomes assessed using comparable instruments and reported at similar follow-up intervals. For most outcomes, substantial heterogeneity in measurement instruments, diagnostic criteria, data formats, and follow-up precluded meta-analysis. Such heterogeneity is expected in systematic reviews of adverse effects, reflecting variability in outcome definitions, populations, and measurement approaches [41]. In line with Cochrane guidance, statistical heterogeneity alone does not preclude meta-analysis when studies address a common clinical question; therefore, pooled estimates were interpreted as average effects across heterogeneous contexts rather than as a single underlying effect. Where heterogeneity could not be explained, findings were interpreted with caution and certainty of evidence was downgraded accordingly.

Outcome harmonization procedures were applied prior to synthesis using a predefined stepwise approach to ensure comparability across heterogeneous outcomes, in line with methodological guidance for adverse effects. Measurement scales were standardized (e.g., pain outcomes on different Visual Analogue Scales [VAS] were converted to a common 0–10 scale), and summary statistics were harmonized by estimating means and standard deviations (SDs) from medians and ranges using established methods [35, 36]. Units of analysis were aligned by aggregating tooth- or site-level data to the study level. When only baseline and follow-up values were available, change scores were derived and SDs were estimated assuming baseline–follow-up correlations. A correlation coefficient of r = 0.5 was assumed for the primary analyses, and sensitivity analyses were conducted using r values ranging from 0.3 to 0.7. When multiple reporting formats were available, post-intervention means were prioritized and change scores were analyzed separately. These procedures ensured transparent and clinically justified handling of heterogeneous outcome measures and are described in detail, with worked examples, in the Supplementary Material (S5 - Outcome harmonization procedures and worked examples).

Random-effects models with restricted maximum likelihood estimation and Hartung–Knapp adjustment were applied. Statistical heterogeneity was quantified using I² and τ². Analyses were conducted in R version 3.4.4 (The R Foundation for Statistical Computing, Vienna, Austria). Publication bias could not be formally assessed due to the small number of studies per outcome; this limitation was considered in the interpretation. Selective outcome reporting was assessed by comparing Methods and Results sections of each study. A structured assessment of selective outcome reporting for harms was conducted (S6 - Assessment of selective reporting and completeness of adverse effects). Although most studies reported at least one adverse effect, the evaluation was typically restricted to a single domain, such as pain, ARR, periodontal parameters, or enamel demineralization. Only a minority of studies assessed multiple adverse outcomes. Overall, the evidence indicates consistent incomplete reporting of harms, as many clinically relevant adverse effects were not assessed or reported, suggesting a moderate to high risk of selective outcome reporting across the included studies. Previous meta-epidemiological work highlights inconsistent harm reporting in orthodontic trials, underscoring the need for standardized adverse-event protocols [42].

For outcomes not suitable for meta-analysis, narrative synthesis was conducted following SWiM guidelines [30], considering direction and magnitude of effects across study design, sample characteristics, interventions, follow-up, and measurement methods. Results are presented in structured tables and figures to highlight patterns and inconsistencies.

### Confidence in cumulative evidence

The overall certainty of evidence was assessed by outcome using the Grading of Recommendations Assessment, Development and Evaluation (GRADE) approach. Two reviewers (CRL and MAVF) independently evaluated certainty across the five GRADE domains, with disagreements resolved by consensus or consultation with a third reviewer (HP). Certainty ratings started at high for randomized evidence and at low for non-randomized evidence and were subsequently downgraded or, when justified, upgraded. Summary of findings tables were generated using GRADEpro GDT, with explicit outcome-level justifications based on pooled estimates or, when meta-analysis was not feasible, on the overall body of evidence [43].

## Results

### Study selection and characteristics

**Fig 2:**
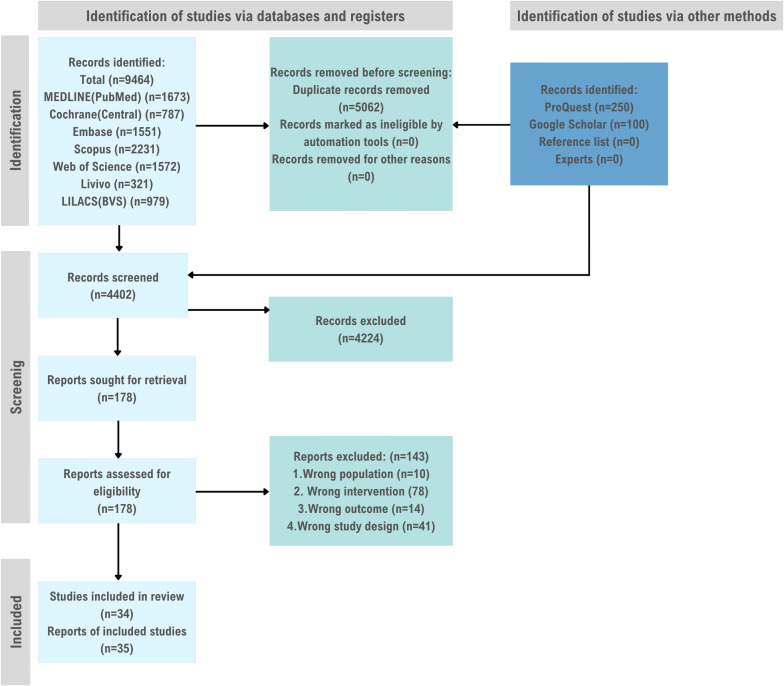
Flow diagram – Identification of studies via databases and registers (last search conducted on May 4, 2025).

A total of 9,464 records were identified, of which 5,062 duplicates were removed. Following screening of the remaining 4,402 records, 178 were selected for full-text screening. The study selection process is summarized in a PRISMA 2020 flow diagram (Fig 2). Ultimately, 34 unique studies (35 reports) were included: 10 RCTs, six NRSs, and 18 cohort studies; one study was published in two separate reports. Excluded studies and reasons for exclusion are provided in S7 - Excluded studies and reasons for exclusion.

The included studies were conducted in 15 countries across four continents: Asia [13, 14, 18, 22, 44–50], the Americas [15, 19, 23, 51–53], Europe [11, 24, 54, 55], and Africa [56], involving 1,636 participants. Sample sizes ranged from 10 [54–56] to 244 [19] including both males and females, with mean ages ranging 13.9 [57] to 45 [46] years. Additional details on the included studies are provided in Tables 1, 2, and 3.

**Table 1:** Study design and population characteristics of the included studies. Legend: AAP/EFP, American Academy of Periodontology/European Federation of Periodontology; BMI, body mass index; CA, clear aligner; CA10, clear aligner 10-day change; CA14, clear aligner 14-day change; CBCT, cone beam computed tomography; CHX, chlorhexidine; CON, conventional appliance; Damon, Damon self-ligating bracket system; d, day(s); EIG, Edgewise and Invisalign Group; EMA, Ecological Momentary Assessment; FA, fixed appliance; FA(FM), fixed appliance with miniscrew; INV, Invisalign; LF, low friction appliance; LO, lingual appliance; LIPUS, low-intensity pulsed ultrasound; MBGR; h, hour(s); m, month(s); w, week(s); N/A, not applicable; N/R, not reported; NRS, numeric rating scale; NPRS, Numeric Pain Rating Scale; QLF, Quantitative Light-induced Fluorescence; RDC/TMD, Research Diagnostic Criteria for Temporomandibular Disorders; VAS, Visual Analog Scale; WSL, white spot lesion.

Study characteristics		Population	
Author (year), Country	Study design	Eligibility criteria	Characteristics Of Sample
Altkuri et al (2024), Saudi Arabia	Cohort	The included participants were patients who were currently undergoing orthodontic treatment, aged between 15 and 65 years, and had no history of previous orthodontic therapy.	The study included 209 participants: 104 in the fixed appliance group (G1) and 105 in the clear aligner group (G2). Nine participants were lost to follow-up. The mean age was 25 years (SD ± 5.7), with a gender distribution of 55.5% male and 44.5% female. Regarding education, 32% had a high school degree or less, 55% held a bachelor’s degree, and 13% had a master’s degree or higher.
Al-Dboush et al (2022), Canada	Cohort	Adult patients treated with clear aligners, having pretreatment and post-treatment CBCT images, mild to moderate crowding (0–6 mm, Little’s irregularity index), Class II or III molar relationship, and no history of orthodontic treatment, periodontal disease, significant medical conditions, asthma, craniofacial anomalies, missing teeth, or maxillary incisor trauma. Only non-extraction and non-surgical cases were included.	21 patients at CA Group (6 male and 15 female) age 35.6 (SD = 11.7)
			21 patients at CA-LIPU Group (7 males and 14 female) age 38.1 (SD = 12.96)
Alam et al (2024), Saudi Arabia	Cohort	Adolescents aged 12–18 years were enrolled based on criteria including the need for orthodontic treatment for malocclusion, no prior history of TMDs, and willingness to participate throughout the study duration.	The study included 200 adolescents: 150 treated with fixed appliances and 50 with aligners. The mean ages were 15.2 years (SD = 1.8) for the fixed group and 15.4 years (SD = 1.7) for the aligner group. Gender distribution was comparable, with slightly more females in both groups.
Albhaisi et al (2020), Jordan	RCT	Healthy patients aged 17–24 years, with Class I malocclusion, mild-to-moderate crowding (≤5 mm), non-extraction treatment, optimal oral hygiene (Plaque Index ≤1, Gingival Index ≤1, and plaque surface area score ≤1.5 in QLF images), up to 3 restored teeth, and no enamel defects such as hypocalcification or hypoplasia.	27 participants at CA Group (7 males, 20 females, mean age 21.2 years) was treated with clear aligners (eonAligner)
			22 participants at FA Group (3 males, 19 females, mean age 21.3 years) received treatment with fixed appliances (Gemini Metal Brackets)
Alcón et al (2021), Spain	Cohort	Adult patients (≥18 years) with permanent dentition, no prior orthodontic treatment, negative bone-dental discrepancy (−6 to −2 mm TSALD in both arches), no missing teeth except third molars, skeletal Class I or mild Class II/III (ANB 0−5°), and a treatment plan predicting interproximal reduction (IPR) of 0.1–0.5 mm per tooth.	70 patients at CA group (33 male and 37 female) age 31.74 (SD = 11.39)
			70 patients at FA group (35 male and 35 female) age 26.97 (SD = 7.23)
Ali et al (2023), Kuwait	NRS	Patients with dental malocclusions seeking orthodontic treatment; patients scheduled for Orthodontic treatment using fixed orthodontic appliances; patients scheduled for Ot using clear aligner treatment; and individuals with a normal occlusion not seeking any type of orthodontic treatment (controls)	24 participants at CA Group (16 males and 8 females, with a mean age of 31.07 years (SD = 1.6)
			24 participants at FA Group (14 males and 10 females, with a mean age of 29.2 years (SD = 2.5)
			25 participants at Control Group (15 males and 10 females, and a mean age of 26.6 years (SD = 0.5)
Almagrami et al (2023), China	Cohort	Adult patients aged≥18 years, mild to moderate crowding, non-extraction treatment, full permanent dentition (excluding third molars), and good quality pre- (T0) and post-treatment (T1) records (CBCT, photographs, and model casts), which were obtained as part of their orthodontic diagnosis and treatment plan.	20 participants at CA Group (mean age 25.15 years, SD = 6.67)
			20 participants at FA Group (mean age 22.33 years, SD = 4.33)
Almasoud (2018), Saudi Arabia	NRS	Adult patients with permanent dentition, no previous orthodontic treatment, no missing teeth, no mucosal or periodontal diseases, good general health, mild to moderate crowding (LII 3–5), and Class I molar relationship were included.	32 participants at CA Group (10 males and 22 females, with a mean age of 28.47 years (SD = 8.17)
			32 participants at FA Group (12 males and 20 females, with a mean age of 23.56 years (SD = 5.44)
Antonio-Zancajo (2021), Spain	NRS	Patients aged 18−40 years with permanent dentition, skeletal Class I or II (mild III with Ricketts convexity ±2 mm), negative TSALD (−6 to −2 mm), no tooth extractions (except third molars), and no prior orthodontic treatment were included. All patients had good oral, periodontal, and general health, with no periodontal pathology at the study’s start.	30 participants at INV Group (aligners: Invisalign), 16 males and 14 females, with a mean age of 33.4 years (SD = 5.1)
			30 participants at LF Group (vestibular low friction brackets), with a mean age of 30.0 years (SD = 7.5)
			30 participants at LO Group (lingual brackets), with a mean age of 30.0 years (SD = 7.5)
			30 participants at CON Group (vestibular conventional brackets), with a mean age of 30.0 years (SD = 7.5)
Buschang et al (2019), United States	Cohort	Only patients for whom high-quality pre and post treatment digital photographs were available and who were in the late mixed or permanent dentitions were includedin the study. No malocclusions were excluded, provided that the gingival thirds of the anterior teeth were visible on the photographs.	244 participants at CA Group, with a mean age of 30.4 years (SD = 14)
			206 participants at FA Group, with a mean age of 29.2 years (SD = 11.5)
Çetin and Akdeniz (2025), Turkey	Cohort	Eligibility criteria included patients with permanent dentition, good oral hygiene, and mild to moderate crowding, without plaque, gingival inflammation, or bleeding. Exclusion criteria included high caries activity, xerostomia, periodontal disease, systemic or craniofacial conditions, poor hygiene, or lack of informed consent.	The study included 50 orthodontic patients with mild to moderate crowding, evenly split between clear aligners (mean age 21.8 years) and fixed appliances (mean age 17.7 years), all with good oral hygiene and no active oral or systemic conditions.
Damasceno Melo et al (2020), Brazil	RCT	Participants 13–35 years old, Angle Class I malocclusion, moderate crowding, and treatment without extraction.	20 participants at CA Group (12 males and 8 females, with a mean age of 23.6 years (SD = 5.65), a Degree of Crowding of 4.69 (SD = 1.35), and a Severity of Malocclusion of 7.70 (SD = 4.66)
			20 participants at FA Group (13 males and 7 females, with a mean age of 20.56 years (SD = 4.51), a Degree of Crowding of 4.99 (SD = 1.88), and a Severity of Malocclusion of 7.50 (SD = 3.18)
Diddige et al (2019), India	RCT	Subjects requiring non extraction fixed orthodontic therapy. Age ranging between 18–30yrs. A full complement of teeth till 2nd molars. Class I malocclusion with Little’s irregularity index between 3–5 mm.	12 participants at CA Group (Smile Align, Mumbai, India), (6 males and 6 females, aged 18–30 years, with a similar degree of teeth irregularity)
			12 participants at FA Group (Damon 3MX, Ormco, Glendora, USA), (6 males and 6 females, aged 18–30 years, with a similar degree of teeth irregularity)
			12 participants at FA Group (Mini Twin, Ormco, Glendora, USA), (6 males and 6 females, aged 18–30 years, with a similar degree of teeth irregularity)
Eissa et al (2018), Canada	NRS	The study included males and females aged 14–25 years, with a specific subgroup consisting of females within the same age range. Participants were required to have minimal to moderate dental crowding, fully developed root apices, and cone-beam computed tomography (CBCT) scans taken both before and after orthodontic treatment.	11 participants at CA Group (Smart Track® aligners – San Jose, California, United States), (5 males and 6 females, with a mean age of 18.34 years (SD = 2.82)
			11 participants at Damon Group (Damon-Q self-ligating brackets – Ormco Corporation, Orange, CA, United States), (4 males and 7 females, with a mean age of 17.71 years (SD = 2.22)
			11 participants at Regular Brackets Group (3M Unitek, California, United States), (6 males and 5 females, with a mean age of 17.34 years (SD = 2.38)
Fraundorf and Kim (2020), United States	Cohort	Patients >18 years old, native English speaker, raised in a monolingual environment, Caucasian, and Class I or Class II malocclusion patients, who were planned for non extraction treatment.	24 patients at CA group (6 male and 18 female) average age 34.88 years.
			20 patients at FA group (5 male and 15 female), average age 38.85 years.
Fujiyama et al (2014), Japan	Cohort	Orthodontic patients >18 years	38 participants at CA Group (10 males and 28 females, with a mean age of 26.64 years (SD = 5.69)
			55 participants at FA Group (20 males and 35 females, with a mean age of 26.45 years (SD = 5.45)
			52 participants at EIG Group (19 males and 33 females, with a mean age of 25.24 years (SD = 6.51)
Gao et al (2021), China	Cohort	Patients with ages greater than 18 years old, good general health, and receiving orthodontic treatment in both arches.	55 participants at CA Group (13 males and 42 females, with a mean age of 26.0 years (SD = 5.47)
			55 participants at FA Group (13 males and 42 females, with a mean age of 24.6 years (SD = 5.20)
Huo et al (2024), China	Cohort	Eligible patients had mild to moderate Class I malocclusion, good general health, no prior orthodontic treatment, and demonstrated high treatment adherence and understanding.	The study included 120 patients with mild to moderate Class I malocclusion, randomly assigned to clear aligners (mean age 21.64 ± 3.97 years) or fixed appliances (mean age 20.22 ± 0.92 years), with a similar gender distribution across groups.
Khalil et al (2023) Egypt	NRS	The study included patients aged 12–18 years with good systemic health, no anti-inflammatory drug use at the start, good oral hygiene, cooperation, and permanent dentition (excluding third molars) Participants had mild to moderate anterior crowding and no prior orthodontic treatment.	10 participants at CA Group (treated with removable clear aligners) Age and gender not reported.
			10 participants at FA Group (treated with conventional fixed orthodontic appliances) Age and gender not reported.
			10 participants at CA+Laser Group (treated with removable clear aligners combined with low-level laser application) Age and gender not reported.
Levrini et al (2013), Italy	RCT	Subjects with Class I skeletal relationship, normo-divergent, Class I molar relationship, and with minimal irregularity in a range of mandibular crowding from 1 to 3, according to Little’s Index were selected.	10 participants at Invisalign Group (3 males and 7 females, with a mean age of 24.6 years (SD = 6.4), treated with Invisalign® aligners (Align Technology, Santa Clara, California)
			10 participants at FA Group (3 males and 7 females, with a mean age of 25.7 years (SD = 3.4), treated with traditional fixed orthodontic appliances)
			10 participants at Control Group (3 males and 7 females, with a mean age of 25.0 years (SD = 3.4), receiving no orthodontic treatment)
Liu & Song (2024), China	Cohort	The study included adolescents aged 10–19 years with permanent dentition who had completed clear aligner treatment and had complete clinical records and quality photographs. Exclusion criteria included prior orthodontic treatment, dental or systemic conditions, craniofacial syndromes, or incomplete data.	The study involved 203 adolescents (mean age 13.9 ± 1.7 years) treated with clear aligners, all with permanent dentition and no prior orthodontic treatment or systemic conditions. Of these, 131 were in the no-WSL group (53 males, 78 females) and 72 in the WSL group (40 males, 32 females). The average treatment duration was 24.1 ± 7.8 months, with complete records and high-quality pre- and post-treatment photographs available for all participants.
Mahayyudin et al (2024), China	Cohort	The study included patients aged 18–35 years with good general and oral health, no significant periodontal disease, and no prior orthodontic treatment. Exclusion criteria included systemic conditions affecting bone metabolism and a history of dental or jaw trauma.	The study included 155 orthodontic patients aged 18–35 years, randomly assigned to clear aligners (n = 78) or fixed appliances (n = 77). Both groups underwent treatment for 12–18 months, with evaluations of alveolar bone and root structure conducted at baseline, 6 months, and post-treatment.
Mertoglu et al (2025), Turkey	RCT	Eligible participants had mild to moderate anterior crowding, fully developed roots, no prior orthodontic treatment, good baseline periodontal health, and no dental anomalies or anterior tooth damage. Exclusion criteria included craniofacial syndromes, systemic diseases, cleft lip/palate, trauma history, poor compliance, mental disability, use of antiseptic mouthwash during treatment, need for scaling and root planing, or delayed anterior tooth movement in the aligner group.	The study included 49 patients with mild to moderate anterior crowding and good baseline periodontal health, randomly assigned to clear aligners (n = 27; mean age 22.57 ± 7.37 years) or fixed appliances (n = 22; mean age 17.05 ± 4.51 years).
Pereira et al (2020), Brazil	RCT	Age between 13 and 35 years; both genders; Angle Class I malocclusion; moderate lower anterior crowding (3–6 mm); and treatment without extraction.	19 participants at CA Group (Align Technology – Santa Clara, California, United States), (11 males and 8 females, with a mean age of 23.60 years (SD = 5.65))
			19 participants at FA Group (3M Unitek, Monrovia, Calif), (12 males and 7 females)
Rossi et al (2024), Canada	Cohort	The study included patients treated with clear aligners (Invisalign®) who had CBCT scans available at both pre-treatment (T0) and post-treatment (T1). For the PBM group, inclusion required use of the OrthoPulse® photobiomodulation device as instructed, with recorded compliance data.	CBCT images from 32 subjects treated with clear aligners were retrospectively analyzed, including 16 patients who used photobiomodulation (PBM) (mean age 29.94 years (SD = 7.67)) and 16 matched controls (mean age 27.38 years (SD = 8.28)), all meeting the study’s inclusion and exclusion criteria.
Rucker et al (2012), United States	Cohort	Participants were at least 18 years old, received treatment in both dental arches, had initial crowding of 2–7 mm per arch, and did not undergo extractions, although interproximal reductions of up to 0.5 mm per contact were permitted.	CA group (Invisalign) with 30 participants (14 male and 16 female), age not reported.
			FA group (Damon System – ORMCO) with 30 subjects (10 male and 20 female), age not reported.
Selvakumar et al (2024), India	RCT	Patients who met the following requirements were eligible: no prior orthodontic treatment, no history of periodontal disease, and excellent overall health.	The study involved 100 patients equally divided between fixed braces and clear aligners, with comparable demographic and baseline periodontal characteristics across both groups.
Schaefer et al (2010), Germany	NRS	All the patients were physically healthy, had no relevant allergies and were not taking antibiotics or medicines that inhibit plaque or saliva production.	31 periodontally healthy subjects (7 male, 24 female) were included in the study in a crossover design and alternately assigned to one of two groups.
Wang et al (2017), China	Cohort	Patients with Angle Class I malocclusion, mild to moderate anterior crowding, and a Discrepancy Index (DI) of 10–20 (ABO assessment) were included. Treatment lasted 1.5–2 years without extractions, with complete radiographic documentation. CBCT scans were performed before treatment, six months after starting, and at the end of treatment to assess ARR.	28 participants at CA Group (14 males and 14 females, with a mean age of 16 years (SD = 3.5))
			28 participants at FA Group (13 males and 15 females, with a mean age of 15 years (SD = 2.3))
White et al (2017), United States	RCT	Class I molar and canine relationships. Nonextraction treatment. Maxillary and mandibular crowding of 4 mm or less. No missing teeth (from second molar to second molar)	23 participants at CA Group (Align Technology’s), (exact numbers of males and females not specified)
			18 participants at FA Group (American Orthodontics’ Radiance), (exact numbers of males and females not specified)
Withayanukonkij et al (2023), Thailand	RCT	Participants were adults aged 18–35 years with anterior open bite (AOB) of 0–4 mm, Angle Class I or II, Skeletal Class I (ANB 0–5°), normo- to hyperdivergent pattern (MPA 23–39°), and healthy periodontal status.	20 participants at CA Group (8 males and 12 females, with a mean age of 21.69 years (SD = 2.67))
			20 participants at FM Group (7 males and 13 females, with a mean age of 21.85 years (SD = 2.71))
Yang et al (2023), China	Cohort	Patients were over 18 years old, periodontally healthy or with stage I periodontitis (based on the 2017 AAP/EFP classification), and underwent orthodontic treatment without tooth extractions, except for third molars.	100 participants at CA Group (15 males and 85 females, with a mean age of 25.13 years (SD = 4.11))
			100 participants at FA Group (19 males and 81 females, with a mean age of 24.14 years (SD = 3.51))
Zhang et al (2025), China	Cohort	Patients included in the study were systemically healthy, with no history of metal allergies, alcohol consumption, or smoking, and had not been exposed to nickel-contaminated environments. They had no metal restorations, a normal BMI, no impacted or congenitally missing teeth (except third molars), and presented with mild malocclusion. All participants were able to understand the study’s purpose, cooperated with the research, and provided informed consent along with their legal guardians.	The study included 120 clear aligner patients—66 adolescents (mean age 14.2) and 54 adults (mean age 25.7)—randomized into study and control subgroups. All participants were systemically healthy, had normal BMI, no harmful habits or metal exposure, a baseline gingival index of zero, and provided informed consent.
Zhao et al (2023), China	RCT	Patients eligible for the trial required clear aligners in both arches, had permanent dentition without congenitally missing teeth (except third molars), and were willing to participate. They required significant orthodontic tooth movements (OTM), such as torque >5°, derotation >5°, or movements >1 mm (e.g., retraction, intrusion, extrusion, expansion) Treatment involved more than 16 aligner sets, with at least one tooth not moved in the ClinCheck plan during the first phase for superimposition analysis using stable teeth.	33 participants at 10-day group (8 males and 25 females, with 32 subjects aged ≤45 years and 1 subject aged >45 years)
			30 participants at 14-day group (6 males and 24 females, with 28 subjects aged ≤45 years and 2 subjects aged >45 years)

**Table 2:** Intervention, comparator, and methodological characteristics of the included studies. Legend: AAP/EFP, American Academy of Periodontology/European Federation of Periodontology; BMI, body mass index; CA, clear aligner; CA10, clear aligner 10-day change; CA14, clear aligner 14-day change; CBCT, cone beam computed tomography; CHX, chlorhexidine; CON, conventional appliance; Damon, Damon self-ligating bracket system; d, day(s); EIG, Edgewise and Invisalign Group; EMA, Ecological Momentary Assessment; FA, fixed appliance; FA(FM), fixed appliance with miniscrew; INV, Invisalign; LF, low friction appliance; LO, lingual appliance; LIPUS, low-intensity pulsed ultrasound; MBGR; h, hour(s); m, month(s); w, week(s); N/A, not applicable; N/R, not reported; NRS, numeric rating scale; NPRS, Numeric Pain Rating Scale; QLF, Quantitative Light-induced Fluorescence; RDC/TMD, Research Diagnostic Criteria for Temporomandibular Disorders; VAS, Visual Analog Scale; WSL, white spot lesion.

Intervention characteristics			Method	
Author (year), Country	Intervention	Comparison	Intervention	Comparison
Altkuri et al (2024), Saudi Arabia	CA	FA	Group 2 (G2) included patients treated with clear removable aligners, all manufactured by Invisalign®. Participants received the pain questionnaire via mobile link twice: once at baseline (tray switch) and again 24 hours later. Study ID numbers were used to match responses from both time points.	N/A
Al-Dboush et al (2022), Canada	CA	CA_LIPUS	CBCT images were taken at T0 (baseline) and T1 (26 months), converted to DICOM format, and processed in ITK-SNAP using “Active Contour Segmentation” for the anterior maxilla, followed by semi-automatic 3D segmentation and manual refinements to isolate maxillary incisors. Each tooth was colored, and volumes recorded. Surface files (VTK) were aligned in 3D Slicer, where crowns were clipped using the Easy Clip module to analyze root volumes. Volumetric ARR was calculated and classified as mild (<10%), moderate (10%−20%), or severe (>20%)	N/A
Alam et al (2024), Saudi Arabia	CA	FA	The study enrolled adolescents aged 12–18 years who required orthodontic treatment for malocclusion, had no history of TMDs, and agreed to participate for the entire study period.	N/A
Albhaisi et al (2020), Jordan	CA	FA	Patients treated with clear aligners received oral hygiene and dietary instructions, including brushing twice daily, avoiding eating with aligners, and cleaning teeth and aligners before reinsertion. Risks like periodontal issues and WSLs were explained, and fluoridated toothpaste, mouth rinse, and interdental brushes were prescribed. Compliance was assessed through plaque and gingival evaluations. Composite resin attachments were bonded using phosphoric acid etching and light curing. Fluorescence images of anterior and premolar teeth were taken before treatment and after 3 months using the QLF system, ensuring consistent and reproducible imaging conditions. Images were analyzed for mineral content changes using specialized software.	N/A
Alcón et al (2021), Spain	CA	FA	The CA group had impressions taken with polyvinyl-siloxane silicone using plastic trays, which were sent to Align Technology for scanning and aligner fabrication via ClinCheck. At the second visit, attachments were bonded as prescribed by the digital plan, and aligners with treatment instructions were provided. Interproximal reduction (IPR) was performed progressively during treatment, up to a maximum of 0.5 mm per tooth.	N/A
Ali et al (2023), Kuwait	CA	FA and Control	Clear aligner treatment (Align Tech. invisalign®, ca, United States) was performed for the correction of malocclusion.	N/A
Almagrami et al (2023), China	CA	FA	Pre- and post-treatment CBCT scans were taken at 120 kV, 5 mA, 0.3 mm voxel size, with a 230 × 170 mm field of view. Patients were positioned upright with proper occlusal and anatomical alignment. DICOM data were imported into Invivo 6 software for 3D analysis. CBCT volumes were oriented perpendicular to each tooth’s long axis, and labial/palatal alveolar bone thickness (ABT) was measured at 3, 6, and 9 mm from the CEJ, while alveolar bone height (ABH) was measured from the CEJ to the alveolar ridge crest. Root length and maxillary incisor inclination (UI-PP) were also assessed. Measurements were performed on pre- and post-treatment scans independently, under supervision by oral and maxillofacial radiologists.	N/A
Almasoud (2018), Saudi Arabia	CA	FA	Patients were treated with Invisalign aligners were provided initial-stage aligners (first aligners)	N/A
Antonio-Zancajo (2021), Spain	CA (INV)	FA (LF, LO and CON)	After appliance placement, patients completed the Short-Form McGill Pain Questionnaire (Ortho-SF-MPQ), marking pain locations on a dental arch diagram and rating pain intensity (none, mild, moderate, severe) and type (throbbing, shooting, stabbing, burning, acute, piercing, cramping, dull, heavy, sensitive, exhausting, cruel, terrible, and frightening)	N/A
Buschang et al (2019), United States	CA	FA	Initial and final intraoral photographs were retrieved, enlarged, and evaluated side-by-side in a dark room. Photographs were used to assess WSL incidence and oral hygiene before and after treatment. Pre-treatment photographs were also used to evaluate fluorosis, which was considered significant if it affected multiple teeth and extended beyond incisal edges. White spots present on both pre- and post-treatment images were deemed developmental and excluded as WSLs. Worsened or new WSLs identified in the post-treatment photographs were recorded. Different criteria were applied for pre- and post-treatment evaluations due to enamel changes from debanding and composite removal.	N/A
Çetin and Akdeniz (2025), Turkey	CA	FA	In the clear aligner group, patients wore aligners for at least 20 hours daily, initially changing them every 15 days and then every 10 days, with selective attachment use for targeted tooth movement; they were instructed to clean aligners with a toothbrush under running water and brush their teeth after meals.	N/A
Damasceno Melo et al (2020), Brazil	CA	FA	Patients treated with Smart Track aligners (Invisalign) followed a virtual treatment plan (ClinCheck Pro, version 5.6) Aligners were replaced every 10 days with a recommended wear time of 22 hours daily, and monitoring occurred monthly. Outcomes from the first 6 months of treatment were recorded and analyzed.	N/A
Diddige et al (2019), India	CA	FA (3MX and Mini Twin)	CA were treated using clear aligners (Smile Align, Mumbai, India) A single set of clear aligners was given to subjects in the aligner group and they were instructed to wear them for a minimum of 22 hours per day for a duration of two week.	N/A
Eissa et al (2018), Canada	CA	FA (Damon and Regular)	The lengths of the maxillary central and lateral incisors were measured before and after treatment using CBCT using Mimics software version19. The incisal edge and root apex were first determined on in the sagittal plane based upon visual inspection only. If necessary, adjustment of their exact position was made in the coronal plane and axial plane. Using the Mimics software, the coordinates of these two points were determined, and the length of each tooth was recorded from the incisal edge to the apex as the distance between these two points.	N/A
Fraundorf and Kim (2020), United States	CA	FA	All patients received bonding and fitting of clear aligners or fixed labial appliances for both arches during the same appointment. Clear aligners were changed weekly, while fixed appliances were adjusted every 4–6 weeks. Speech performance was assessed at three time points: before (T0), immediately after (T1), and 2 months after (T2) fitting. Patients read the “Grandfather Passage” under standardized conditions, and recordings were processed into mp3 files for evaluation.	N/A
Fujiyama et al (2014), Japan	CA	FA and EIG	Patients were instructed to wear the aligners for at least 20 hours per day and to record their pain levels during three treatment stages using a 10-cm Visual Analog Scale (VAS) The scale ranged from 0 (no pain) to 100 (maximum pain), with 50 indicating moderate pain. The scoring method was explained to all patients before the study.	N/A
Gao et al (2021), China	CA	FA	A total of 55 patients treated with clear aligners between 2013 and 2015 were enrolled. Attachments and interproximal reductions were applied on day 0, and participants were followed for the first 14 days, with data collected before and during this period.	N/A
Huo et al (2024), China	CA	FA	Patients in the experimental group used clear aligners made from 3D digital scans and computer simulations, provided by Shanghai Times Angel Medical Technology. The aligners, made of thermoplastic polyurethane, were changed every two weeks and worn for at least 20 hours daily under professional supervision. Both groups underwent 12 months of treatment, with CBCT scans taken before and after by the same examiner, and the average of three measurements per patient was calculated.	N/A
Khalil et al (2023) Egypt	CA	FA and CA+Laser	CBCT scans were obtained before treatment (T1) and 6 months later (T2) Root reconstruction of the lower incisors was performed in 3D using Mimics software (version 18, Materialise) Threshold values for root segmentation were adjusted for consistency across scans, followed by manual segmentation to isolate the lower incisors from surrounding structures. The isolated teeth were converted into 3D models, separated from adjacent teeth, and decapitated at the cementoenamel junction to create isolated root models. Root volume was then measured in millimeters.	N/A
Levrini et al (2013), Italy	CA	FA and Control	One month before starting orthodontic therapy, patients underwent professional oral hygiene and received standardized oral hygiene instructions from an experienced dental hygienist, reinforced during check-ups. Electric toothbrushes were prohibited, and patients were required to use an orthodontic brush (Bass technique, 2 minutes) and dental floss three times daily. Invisalign® aligners were worn for 20 hours a day and replaced every two weeks as per the individualized treatment plan.	
Liu & Song (2024), China	CA-No WSL	CA-WSL pre	The treatment followed standard clinical protocols, including digital planning with ClinCheck®, attachment bonding with phosphoric acid and composite resin, and aligner use for over 20 hours daily across an average of 24.1 ± 7.8 months. Oral hygiene instructions were given, but no additional preventive measures were applied, as the study aimed to assess the natural occurrence of white spot lesions (WSLs) and related risk factors.	
Mahayyudin et al (2024), China	CA	FA	The treatment involved custom-made aligners worn 20–22 hours daily, with each set changed approximately every two weeks. The duration varied by case, averaging between one year and one and a half years.	N/A
Mertoglu et al (2025), Turkey	CA	FA	Patients in the clear aligner group were treated with the Invisalign® system, starting anterior tooth movement from the first aligners. Attachments were bonded using Microfill and brace paste primers, with aligners changed every two weeks during the treatment.	N/A
Pereira et al (2020), Brazil	CA	FA	Group CA was treated with Invisalign® aligners (Align Technology, Santa Clara, CA, USA), with 3D planning performed using ClinCheck Pro (version 5.6) based on patient needs and manufacturer guidelines. Aligners were replaced every 10 days.	N/A
Rossi et al (2024), Canada	CA-PBM	CA	CBCT imaging from 32 subjects who received comprehensive orthodontic treatment with clear aligners (16 consecutively treated PBM [Biolux Research, Fremont, Calif] patients who met the inclusion/exclusion criteria and 16 matched control patients) were retrospectively compared for this study.	
Rucker et al (2012), United States	CA	FA	The CA group was treated with aligners and instructed to wear them for at least 23 hours daily, replacing each aligner every two weeks.	N/A
Selvakumar et al (2024), India	CA	FA	Patients in the clear aligners group were instructed to wear their aligners for 22 hours daily, removing them only for meals and oral hygiene. They received guidance on proper oral care, including the use of fluoride toothpaste and interdental brushes.	N/A
Schaefer et al (2010), Germany	CA	CA-CHX	All patients were physically healthy, with no significant allergies or use of antibiotics or medications affecting plaque or saliva production. The study had two 3-month periods separated by a 2-month washout phase to prevent residual effects of the chlorhexidine mouthwash. In the first period, one group used 0.06% chlorhexidine mouthwash while the other had no intervention, and these conditions were reversed in the second period.	N/A
Wang et al (2017), China	CA	FA	The experimental group was treated with invisible aligners, involving preoperative molding, CBCT, digital imaging, treatment planning with the manufacturer, and instructions for wearing the aligners at least 20 hours daily. Orthodontic treatment and root length measurements on CBCT were performed by the same orthodontist for consistency. CBCT parameters included an 18-second scanning time, 3.4-second exposure time, 0.3 mm focal spot, 15 mA tube current, and 110V tube voltage.	N/A
White et al (2017), United States	CA	FA	Patients were treated by two clinicians, with one performing most ClinChecks and banding/debonding procedures. For the aligner group, either polyvinylsiloxane impressions or iTero scans were used, and aligners were fabricated with Align Technology’s ClinCheck software. Composite attachments were placed during the initial aligner delivery. Patients received two sets of aligners, wearing each set for 22 hours daily over two weeks, switching to the second set on day 15. Monthly evaluation appointments were conducted, with no significant changes to the methods during the trial.	N/A
Withayanukonkij et al (2023), Thailand	CA	FA (FM)	An intraoral scanner created an STL file, which was imported into 3Shape OrthoAnalyzer, and models were printed using a 3D printer. Attachments were designed to optimize open bite closure, with maxillary anterior teeth extruding 0.2 mm per aligner, changed every 3 weeks. Clear aligners were fabricated from 1-mm Duran thermoplastic sheets. Patients followed a squeezing protocol, clenching on the aligner for 1 minute per session (5 seconds of clenching at 80% MBF followed by 5 seconds of rest, repeated six times), performed at least five times daily. Aligners were worn for at least 22 hours daily, except during brushing and eating.	N/A
Yang et al (2023), China	CA	FA	All patients had complete intraoral photographs, panoramic radiographs, and digital models taken before and after treatment, which was completed entirely with clear aligners.	N/A
Zhang et al (2025), China	CA-Adults + control group	CA-Adolescents + control group	Adult participants were divided into a study group and a control group. The study group received personalized oral hygiene education and guidance at each follow-up, including the Bass brushing technique, use of floss, interdental brushes, and oral irrigators, with compliance monitored through self-reports and clinical observation. The control group maintained their usual oral hygiene practices and received routine periodontal care, including scaling, root planing, plaque disclosure, and general feedback, but no individualized oral hygiene instruction.	Adolescent participants were divided into a study group and a control group. The study group received personalized oral hygiene education at each follow-up, including instruction in the Bass brushing technique, use of floss, interdental brushes, and oral irrigators, along with educational materials. Compliance was monitored through clinical observation and self-reports, with tailored recommendations as needed. The control group continued their usual oral hygiene routines and received standard periodontal care, including scaling and root planing, with plaque disclosure for general feedback but no structured oral hygiene instruction.
Zhao et al (2023), China	CA10	CA14	Participants changed their clear aligners every 10 days, wearing each aligner for at least 22 hours daily and using “chewies” for 5 minutes after inserting a new aligner. Compliance was monitored through a smartphone app where patients recorded their daily wear time. The trial covered stages 1–16 of aligner treatment, lasting 160 days.	Participants changed their clear aligners every 14 days, wearing each aligner for at least 22 hours daily and using “chewies” for 5 minutes after insertion. Compliance was tracked through a smartphone app where patients recorded daily wear time. The trial spanned stages 1–16 of aligner treatment, lasting 224 days.

**Table 3:** Adverse effects, outcome measures, follow-up, and funding information of the included studies. Legend: AAP/EFP, American Academy of Periodontology/European Federation of Periodontology; BMI, body mass index; CA, clear aligner; CA10, clear aligner 10-day change; CA14, clear aligner 14-day change; CBCT, cone beam computed tomography; CHX, chlorhexidine; CON, conventional appliance; Damon, Damon self-ligating bracket system; d, day(s); EIG, Edgewise and Invisalign Group; EMA, Ecological Momentary Assessment; FA, fixed appliance; FA(FM), fixed appliance with miniscrew; INV, Invisalign; LF, low friction appliance; LO, lingual appliance; LIPUS, low-intensity pulsed ultrasound; MBGR; h, hour(s); m, month(s); w, week(s); N/A, not applicable; N/R, not reported; NRS, numeric rating scale; NPRS, Numeric Pain Rating Scale; QLF, Quantitative Light-induced Fluorescence; RDC/TMD, Research Diagnostic Criteria for Temporomandibular Disorders; VAS, Visual Analog Scale; WSL, white spot lesion.

Outcome		Measurement					Additional Information
Author (year), Country	Adverse effect	Tool	Type or Location	Unit	Follow_up	Duration	Funding/Conflict of interest
Altkuri et al (2024), Saudi Arabia	Pain	NPRS	Intensity	11-point Numeric Pain Rating scale	Baseline, 24h	24 hours	The authors declare no conflict of interest, financial or otherwise.
	Pain descriptor (pulsanting, eletrical, sttabing, sharp, pressure, bite and touch, exhausting, disgustig, scary, severing)	NRS	Mild, moderate, severe	zero= no pain, 1-3= mild, 4-6= moderate, or 7-10= severe			
	Overall pain	Present Pain Index	Mild, discomforting, distressing, horrible	“no pain”, “mild”, “discomforting”, “distressing”, “horrible”, “excruciating”			
Al-Dboush et al (2022), Canada	Volumetric ARR	CBCT	Upper Central and Lateral Incisors	mm3	Baseline, 26m	All treatment	The authors report no commercial, proprietary or financial interest in the products or companies described in this article.
Alam et al (2024), Saudi Arabia	Temporo mandibular joint disorders	RDC/TMD	Incidence	%	Baseline, 6m, 12m	12 months	The study received no financial support or sponsorship, and the authors declared no conflicts of interest.
			Severity	Mild, moderate, severe	12m		
Albhaisi et al (2020), Jordan	Enamel demineralization	QLF	fluorescence loss	%	Baseline, 3m	3 months	Funding: Deanship of Scientific Research/Jordan University of Science and Technology. The QLF system was supported by a grant from the Scientific Research Fund, Ministry of Higher Education and Research, Jordan. No conflit of interest.
	White spot lesions	QLF	surface area	%			
	Plaque	QLF (red)	surface area	%			
Alcón et al (2021), Spain	Periodontal pain	VAS	first, fourth and eigth month	0 to 10 cm	4h, 8h, 24h, 2d, 3d, 4d, 5d, 6d, 1w	7 days	Funding: This research received no external funding. No conflict of interest.
			overall	0 to 10 cm	1m, 2m, 3m, 4m, 5m, 6m, 7m, 8m, 9m, 10m, 11m, 12m	12 months	
Ali et al (2023), Kuwait	Pain	VAS	N/A	0 to 10 cm	Baseline, 24h, 1m	30 days	Funding: There was no source of funding for the present study. No conflict of interest.
Almagrami et al (2023), China	Bone height and thickness	CBCT	Central incisors labial and palatal, lateral incisors labial and palatal		Baseline, 26m	25,85 months	NR
	ARR		Central and lateral incisors	0° = No ARR (0 mm) 1° = Slight blunting of the root apex (1–2 mm) 2° = Moderate root apex blunting up to one-fourth root length (2 mm-1/4 root length) 3° = Excessive blunting of the root apex beyond one-fourth of the length of the root (>1/4 root length)			
Almasoud (2018), Saudi Arabia	Pain	VAS	N/A	0 to 10 cm	4h, 24h, 3d, 1w	1 week	No conflict of interest.
Antonio-Zancajo (2021), Spain	Periodontal pain	Short-Form McGill Pain Questionnaire	Both arch’s	%	4h, 8h, 24h, 2d, 3d, 4d, 5d, 6d, 1w	7 days	Funding: This research received no external funding. No conflict of interest.
		Questionnaire of intensity	intense, mild, moderate, no pain	%	4h, 8h, 24h, 2d, 3d, 4d, 5d, 6d, 1w	7 days	
	Periodontal type of pain	Questionnaire of type of pain	acute, dull, no pain, other types of pain, sensitive, stabbing	%	4h, 8h, 24h, 2d, 3d, 4d, 5d, 6d, 1w	7 days	
Buschang et al (2019), United States	White spot lesions	Frequencie	Mandibular and maxillary	%	Baseline, 18m	1,5 years	NR
Çetin and Akdeniz (2025), Turkey	Carie+filling	Bite-wing radiographs	Proximal	n	Baseline, 6m	6 months	The authors reported no conflicts of interest and received no specific funding for the study.
			Non-proximal				
	Decay, Missing, Filled Teeth Index	DMFT index		n			
Damasceno Melo et al (2020), Brazil	Perception of speech	VAS	N/A	0 to 10 cm	After, 3d, 1m, 6m	6 months	NR
	Speech evaluation	MBGR	N/A	%	After, 3d, 1m, 6m	6 months	
Diddige et al (2019), India	Pain	VAS	N/A	0 to 100 mm	4h, 24h, 3d, 1w	7 days	NR
Eissa et al (2018), Canada	ARR	CBCT	Upper central and lateral incisors	mm	Baseline, 18m	All treatment	Funding: The authors would like to thank Align Technology, Inc. for supporting this research through the 2016 Align Technology Research Awards Program. No conflicts of interest.
Fraundorf and Kim (2020), United States	Speech performance	Digital sonography	N/A	Upper boundary frequency (UBF; the maximum frequency ofthe band width of the fricative) of the middle 50 ms ofthe /s/ sound.	Baseline, After, 2m	2 months	NR
		Six certified speech and language pathologis		Likert scale: (4)severely altered speech production, (3)moderately altered speech production, (2)mildly altered speech production, an (1)normal speech production.	Baseline, After, 2m	2 months	
		Patient Questionnaire		Visual linear scale (100 mm)	Baseline, After, 2m	2 months	
Fujiyama et al (2014), Japan	Pain	VAS	first. second and third stage	0 to 100 mm	Baseline, 6h, 12h, 24h, 2d, 3d, 4d, 5d, 6d, 1w		No conflicts of interest.
Gao et al (2021), China	Pain	VAS	N/A	0 to 100 mm	24h, 2d, 3d, 4d, 5d, 6d, 1w, 2w	2 weeks	The study was funded by the 2013 International Clear Aligner Research Awards, the National Natural Science Foundation of China, and other Chinese institutions, with no involvement from the funders in the research process or manuscript preparation.
Huo et al (2024), China	Root resoption	CBCT	Upper central incisor, upper lateral incisor and canine, lower central incisor, lower left lateral incisor, and lower canine.	mm	Baseline, 12 months	12 months	
	Bone height and thickness		Cervical (UBH), middle (UMH), and apical (ULH) regions of the upper and lower anterior teeth	mm			
Khalil et al (2023) Egypt	ARR	CBCT	Lower central and lateral incisors, lower canines	mm3	Baseline, 6m	6 months	Funding: the study was totally funded by the author number one.
Levrini et al (2013), Italy	Periodontal health status	Bleeding on probing	N/A	20 seconds after probing (absent = 0, present = 1)	Baseline, 1m, 3m	90 days	NR
	Periodontal health status	Pocket probing depth		mm	Baseline, 1m, 3m	90 days	
	Plaque Index	Silness&Loe		0 = no plaque/debris on inspection and probing 1 = thin film of plaque only visible after probing 2 = ribbon-like layer of plaque covering the gingival sulcus with no involvement of interproximal dental space 3 = thick layer of plaque clearly visible at inspection and involving an interproximal dental space	Baseline, 1m, 3m	90 days	
Liu & Song (2024), China	White spot lesion	Intra-oral photographs	Incidence	%	Baseline, 24m	24 months	The study was funded by the National Clinical Research Center for Oral Diseases (grant LCA202009) and the Key Research and Development Program of Shaanxi Province (grant 2021SF-048). The authors declare no conflicts of interest and have approved the manuscript for publication.
Mahayyudin et al (2024), China	Apical root resoption	N/R		mm	Baseline, 6m, 18m	18 months	N/R
	Clinically Significant ARR (>2 mm)			%	18m		
	Alveolar bone density			HU	Baseline, 6m, 18m		
	Alveolar bone volume			mm3	Baseline, 6m, 18m		
Mertoglu et al (2025), Turkey	ARR	Periapical radiographs	Upper right central incisor, upper left central incisor, upper left lateral incisor, upper right lateral incisor, lower right lateral incisor, lower right central incisor, lower left central incisor, lower left lateral incisor.	mm	Baseline, 3m, 6m	6 months	The study was funded by the Bezmialem Vakif University Scientific Research Projects Coordination Unit (20211212), and the authors declared no conflict of interest.
	Periodontal health status	Probing depth	Ramfjord tooth	mm			
		Bleeding on probing		%			
	Plaque Index	Silness&Loe	Ramfjord tooth	Plaque levels were scored as follows: 0 – no visible plaque; 1 – a thin film of plaque along the gingival margin; 2 – moderate plaque accumulation, including within the sulcus; and 3 – heavy plaque accumulation in the sulcus or pocket along the gingival margin.			
	Pain	VAS/5-question survey	N/A	0 to 100 mm	Baseline, 4h, 24h, 1w, 2w, 1m, 3m, 6m		
	Chewing performance	VAS/9-question survey	N/A				
Pereira et al (2020), Brazil	Awake Bruxism	EMA	N/A	a) I am not touching my teeth; b) I am not touching my teeth,but I feel my muscles are contracted; c) I am slightly touching my teeth; d) I am clenchingmy teeth; or e) I am grinding my teeth. (a+b+c+d =%)	Baseline, After, 1m, 2m, 3m, 6m	180 days	No conflicts of interest.
Rossi et al (2024), Canada	ARR	CBCT	Upper right canine, upper right lateral incisor, upper right central incisor, upper left central incisor, upper left lateral incisor, upper left canine, lower left canine, lower left lateral incisor, lower left central incisor, lower right central incisor, lower right lateral incisor, lower right canine.	mm3	Baseline, 24m	24 months	N/R
Rucker et al (2012), United States	Pain	VAS	N/A	10 bubble	24h, 1w, 2w, 3w	3 weeks	NR
Selvakumar et al (2024), India	Plaque	Plaque index		PI	Baseline, 3m, 6m	6 months	The study had no financial support or sponsorship, and no conflicts of interest were declared.
	Periodontal health status	Gingival index		GI			
		Probing depth		mm			
Schaefer et al (2010), Germany	Halitosis	Halimeter® sulfide monitor	N/A	Normal (70–110 ppb) and Malodor (>150 ppb)	1m, 2m, 3m	3 months	NR
		Loe&Silness		0 = no inflammation or redness, 1 = slight inflammation, slight redness, slight swelling, 2 = moderate inflammation with redness, edema and bleeding, 3 = severe inflammation, redness, edema, tendency to spontaneous bleeding, ulceration.	1m, 2m, 3m	3 months	
Wang et al (2017), China	ARR	Prevalence	N/A	%	6m, 12m	12 months	NR
White et al (2017), United States	Discomfort	VAS	initial delivery, first and second month	0 to 100 mm	Baseline, 24h, 2d, 3d, 4d	3 months	Fundind: This research was partially funded by the Robert E. Gaylord Endowed Chair in Orthodontics and by Align Technology.
Withayanukonkij et al (2023), Thailand	ARR	CBCT	Upper first molars (distal/mesial bucal root and palatine root)	mm	Baseline, 6m	6 months	NR
Yang et al (2023), China	Open gingival embrasures	Photograph	mandibular and maxillary	mm2 (Height of an OGE was the distance between the uppermost margin of interdental papilla and the contact point of central incisors)	26m	26,39 months	Funding: this study was supported by Nanjing Medical Science and technique Development Foundation and Nanjing Clinical Research Center for Oral Diseases. The funding body supported data analysis and writing the manuscript. No conflicts of interest.
Zhang et al (2025), China	Periodontal health status	Gingival Index		0–3, where 0 represents gingival health, 1 represents mild inflammation, 2 represents moderate inflammation, and 3 represents severe inflammation	Baseline, 6m	6 months	The study was supported by the Zhaoqing Science and Technology Innovation Guidance Category Project ((2020) No. 26 202004031206), and the authors declared no conflicts of interest regarding its publication.
	Gingivitis	Gingival Index	Incidence	%			
Zhao et al (2023), China	Pain	VAS	10 days	0 to 10 cm	24h, 2d, 3d, 1w	160 days	Funding: This work was supported by the Invisalign Award and the Orthodontic National Key Clinical Specialty Construction Program of China, West China Hospital of Stomatology, Sichuan University. However, they did not participate in the conduct of the research, analysing the data nor in writing the manuscript. No conflict of interest.
			14 days	0 to 10 cm	24h, 2d, 3d, 1w	244 days	

### Risk of bias within the studies

**Fig 3:**
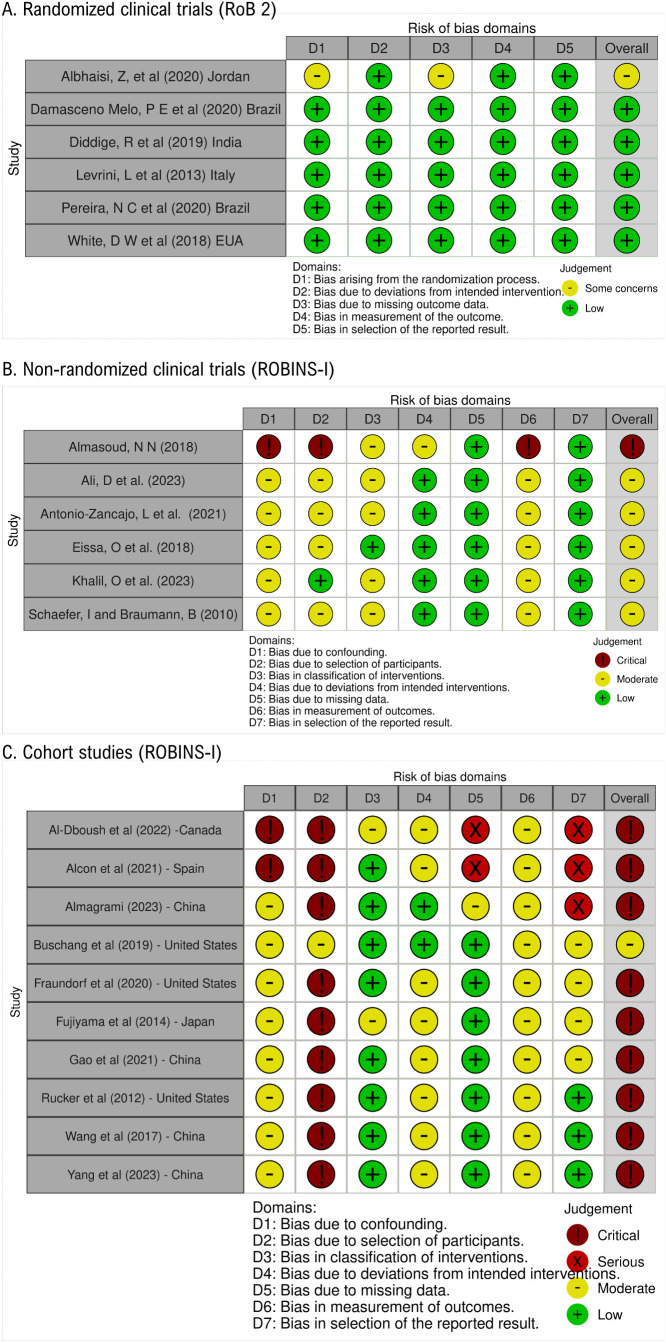
Risk of bias assessment – (A) Randomized Clinical Trials assessed by RoB 2 tool. (B) Non-Randomized Clinical Trials assessed by ROBINS-I tool. (C) Cohort Studies assessed by ROBINS-I tool.

The risk of bias assessment indicated that most RCTs were at low risk of bias, supported by robust methodological approaches. Among NRSs, five were assessed as having a moderate risk of bias, and one as having a high risk, primarily due to selection bias and lack of blinding. Cohort studies generally demostrated a moderate to high risk of bias, with concerns related to participant selection, control of confounding, and blinding; however, one study was rated as having a moderate risk with robust outcome measurement. Further details are presented in Fig 3.

### Synthesis of results

Based on the predefined synthesis criteria described in the Methods, meta-analyses were conducted for outcomes with sufficient methodological consistency (pain/discomfort, ARR, and plaque), while all other outcomes were synthesized narratively due to substantial heterogeneity in definitions, measurement methods, and follow-up periods.

**Fig 4:**
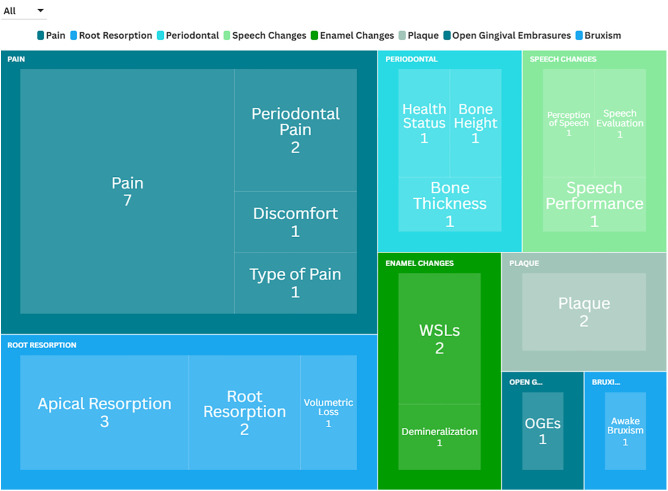
Study-based frequency distribution of adverse outcomes associated with orthodontic aligners.

The studies were categorized according to the study design (RCTs, cohort studies, and NRSs), assessed outcomes, measurement methods, and follow-up periods. The analyzed outcomes included awake bruxism (AB) [58], chewing performance [59], enamel changes (Caries/filling, enamel demineralization, and WSL) [18–20, 57], halitosis [24], open gingival embrasures [22], pain (discomfort, general pain, periodontal pain and type of pain) [11, 13, 46–50, 52, 53, 55, 60], periodontal health status (bone height/thickness, bleeding on probing, gingivitis, probing pocket depth) [16, 17, 44, 54, 61, 62], plaque (plaque and plaque index) [18, 54], ARR (apical and volumetric) [14–16, 44, 45, 51, 56, 59, 62, 63], speech (perception, evaluation, and performance) [23, 53], and temporomandibular joint disorders (TMD) [24] (Fig 4).

Meta-analysis was feasible for only three outcomes: pain/discomfort, ARR, and plaque. A total of 10 studies assessed pain or discomfort associated with removable aligner therapy [13, 46–50, 52, 53, 55, 60]. Apical root resorption was evaluated in six studies [14–16, 44, 59, 62], while three used three-dimensional (3D) techniques to measure volumetric loss [51, 56, 62]. Plaque was evaluated in three studies [17, 54, 59]. The following sections present the quantitative effect estimates across different follow-up intervals. All R scripts used for the statistical analyses are available in S8 - R Scripts.

### Quantitative synthesis

#### Pain and discomfort meta-analysis.

**Fig 5:**
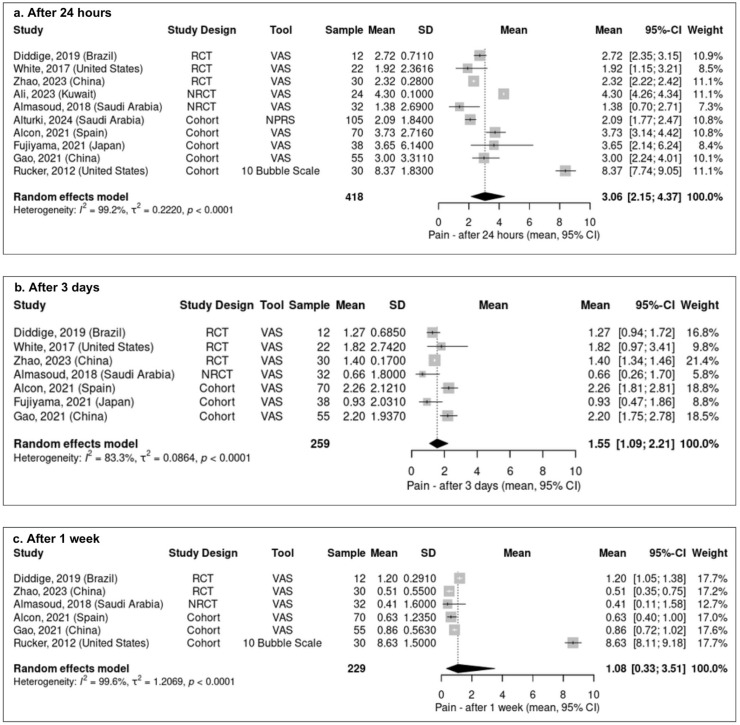
Random-effects meta-analysis of pain/discomfort at 24 hours (k = 10), 3 days (k = 7), and 1 week (k = 6). Legend: (a) Mean and SD of Pain/Discomfort after 24 hours. (b) Mean and SD of Pain/Discomfort after 3 days. (c) Mean and SD of Pain/Discomfort after 1 week.

Pain peaked at 24 hours (mean 3.06, 95% CI: 2.15 to 4.37) (Fig 5a), decreased to 1.55 (95% CI: 1.09 to 2.21) at three days (Fig 5b), and further declined to 1.08 (95% CI: 0.33 to 3.51) by one week (Fig 5c). High heterogeneity (I² > 80%) was observed at all time points.

#### Apical root resorption meta-analysis.

**Fig 6:**
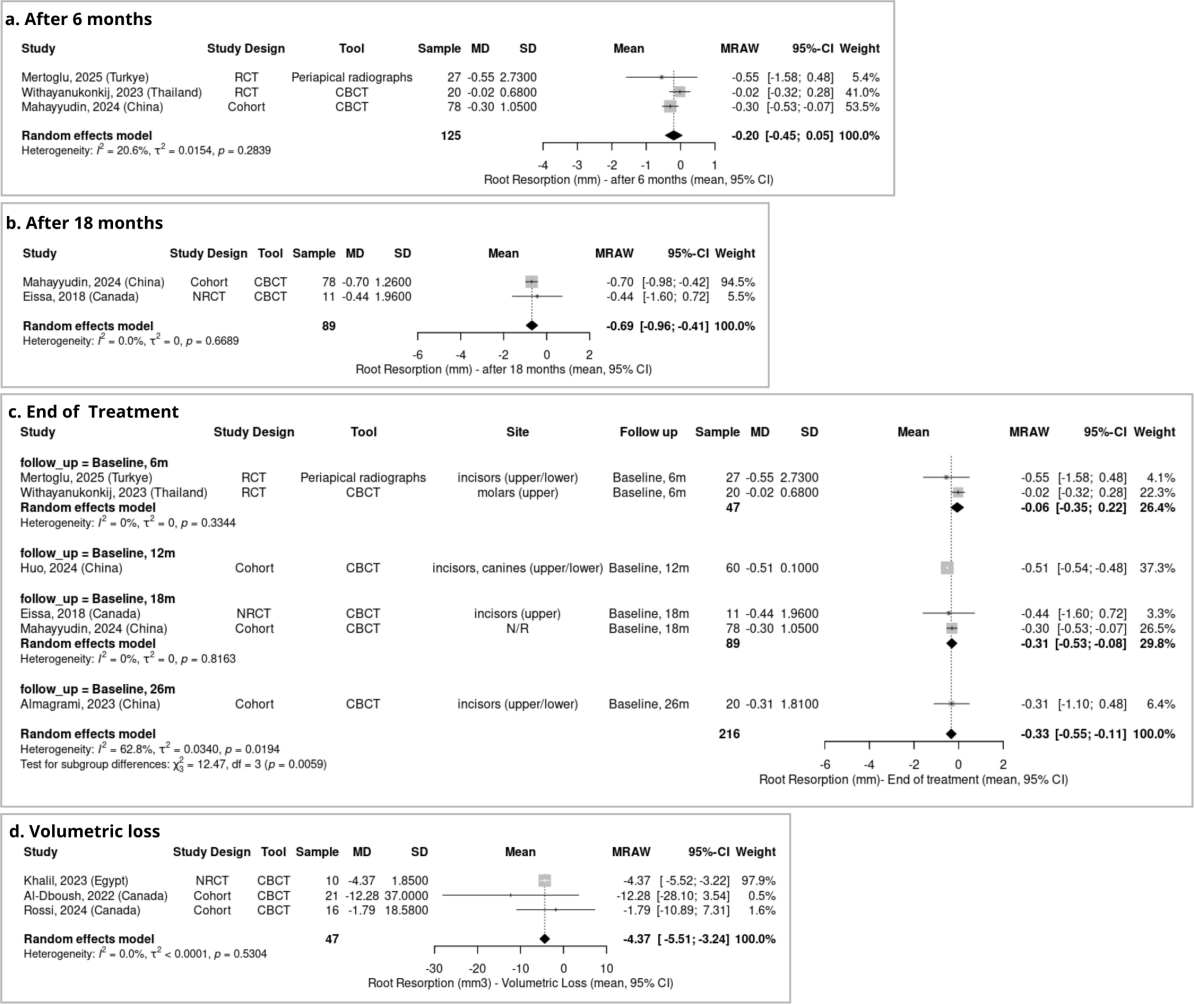
Random-effects meta-analysis of apical root resorption (root length change, mm) at 6 months (k = 3), 18 months (k = 2), and end of treatment (k = 6), and volumetric root resorption (mm³) (k = 3). Legend: (a) Apical root resorption at 6 months; (b) at 18 months; (c) at end of treatment; (d) volumetric root resorption (mm³).

Meta-analysis of ARR showed a mean loss of −0.33 mm (95% CI: −0.55 to −0.11) (Fig 6a). At six months, the estimate was −0.20 mm (95% CI: −0.45 to 0.05) (Fig 6b). Subgroup analyses by follow-up duration showed that resorption ranged from −0.06 mm at six months (95% CI: −0.35 to 0.22) to −0.51 mm at 12 months (95% CI: −0.54 to −0.48). Apical root resorption was also observed at 18 months (−0.31 mm; 95% CI: −0.53 to −0.08) and 26 months (−0.31 mm; 95% CI: −1.10 to 0.48). Moderate heterogeneity was present (I² = 62.8%), and subgroup differences by follow-up duration were statistically significant (p = 0.0059). At 18 months, no heterogeneity was detected (I² = 0%), and the mean reduction was −0.69 mm (95% CI: −0.96 to −0.41) (Fig 6c). Volumetric analysis showed a mean ARR of −4.37 mm³ (95% CI: −5.51 to −3.24) by the end of follow-up (Fig 6d).

#### Plaque meta-analysis.

**Fig 7:**
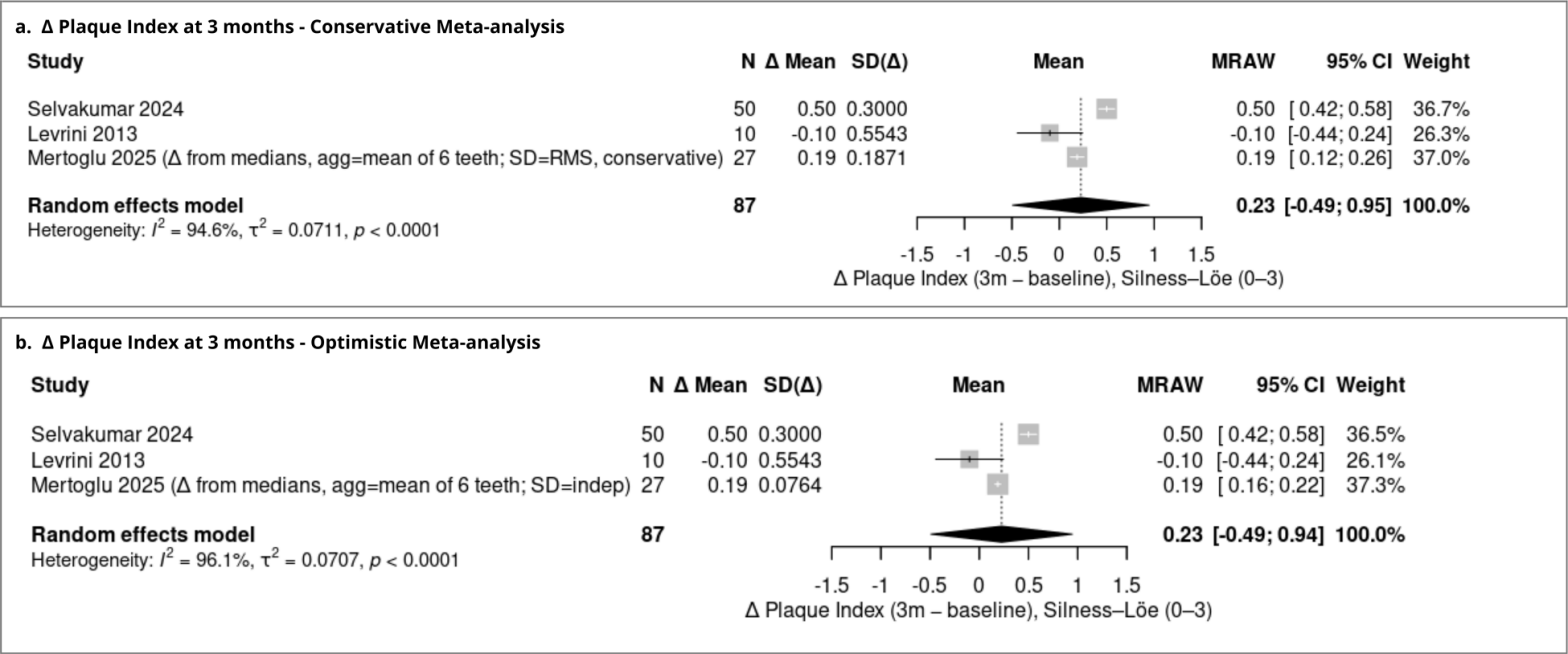
Random-effects meta-analysis of change in Plaque Index at 3 months (Silness–Löe 0–3), including conservative and optimistic imputations (k = 3 studies). Legend: (a) Conservative meta-analysis using imputed means and SDs; (b) Optimistic meta-analysis. Mertoglu et al. included based on imputed data from medians.

Of the three included studies, one RCT [46] assessed two Ramfjord sites and showed a reduction in Plaque Index at 3 months (Δ −0.10; 95% CI −0.44 to 0.24). In contrast, increases in Plaque Index were observed in the multicenter study [59] and in the study with imputed statistics [51] (Fig 7). Substantial heterogeneity was observed (I² = 95–96%).

#### Narrative synthesis.

The findings of the included studies for outcomes not suitable for meta-analysis are summarized in Table 4.

**Table 4:** Key findings on adverse effects associated with the use of orthodontic aligners. Legend: DMFT, Decay, Missing, Filled Teeth Index; VAS, Visual Analog Scale; NPRS, Numeric Pain Rating Scale; NRS, Numeric Rating Scale; EMA, Ecological Momentary Assessment; QLF, Quantitative Light-induced Fluorescence; QLF (red), QLF with red fluorescence mode; CBCT, Cone beam computed tomography; RDC/TMD, Research Diagnostic Criteria for Temporomandibular Disorders; MBGR, Orofacial Myofunctional Examination; N/R, Not reported.

Outcome	Study	Tool	Unit of Measurement	Follow-up	Baseline	SD Baseline	Maximum Value (m or %)	SD	Moment	Change	SD Change	p value	Percentage	Conclusion
Awake Bruxism	Pereira et al (2020), Brazil	EMA	%	Baseline, After, 1m, 2m, 3m, 6m	55.90	30.90	56.00	28.30	After	0.10	41.90	0.075	0.18%	Increase of 0.18% in the frequency of awake bruxism immediately after.
Carie/filling	Çetin et al (2025),Turkye	Decay, Missing, Filled Teeth Index (DMFT)	Number	Baseline, 6m	5.24	5.61	5.76	5.83	6 months	0.52	5.72	<0.05	9.92%	Increase of 9.92% in the number of DMFT Index after 6 months of treament.
Chewing performance	Mertoglu et al (2025), Turkey	VAS/9-question survey	0 to 100 mm	Baseline, 4h, 24h, 1w, 2w, 1m, 3m, 6m	10.93	02.02	19.56	4.63	24 hours	8.63	04.02	<0.001	78.96%	Chewing performance deteriorated by 78.9% within 24 hours of appliance placement.
Discomfort	White et al (2017). United States	VAS	0 to 100 mm	Baseline, 24h, 2d, 3d, 4d	10.76	10.90	19.36	22.51	2 days	8.60	25.01	0.041	79.93%	Increase of 79.93% in discomfort after 2 days of treatment.
Enamel demineralization	Albhaisi et al (2020), Jordan	QLF	%	Baseline, 3m	−7.30	4.40	−7.70	4.90	3 months	0.40	8.79	0,283	5.48%	Increase of 5.48% in enamel demineralization.
Halitosis	Schaefer et al (2010), Germany	Halimeter® sulfide monitor	Normal (70–110 ppb) and Malodor (>150 ppb).	1m, 2m, 3m	NR	NR	42.46	14.78	2 months	N/A	N/A	0.109	N/A	Did not reveal any evidence of halitosis; the findings are within the normal range.
Open gingival embrasures	Yang et al (2023), China	Photograph	%	26m	NR	NR	36.50	N/A	26 months	N/A	N/A	0.019	N/A	The incidence of OGEs were 36.50% at 26 months of treatment.
Pain	Ali et al (2023), Kuwait	VAS	0 to 10 cm	Baseline, 24h, 1m	NR	NR	4.30	0.10	24 hours	4.30	0.10	>0.01	N/A	Peak of pain in the first 24 hours with values of 4.30 (SD 0.10).
	Almasoud (2018), Saudi Arabia	VAS	0 to 10 cm	4h, 24h, 3d, 1w	NR	NR	1.38	2.69	24 hours	1.38	2.69	0.001	N/A	Peak of pain in the first 24 hours with values of 1.38 (SD 2.69).
	Altkuri et al (2024), Saudi Arabia	NPRS	11-point Numeric Pain Rating scale	Baseline, 24h	0.51	0.90	02.09	1.84	24 hours	1.58	1.59	<0.0001	309.8%	Pain increased by 309.8% at 24 hours compared to baseline.
		NRS	Pulsating, Electrical, Stabbing, Sharp, Pressure, Bite and touch, Exhausting, Disgusting, Scary, Severing (Mild, Moderate, Severe)	Baseline, 24h	Mild Pressure	N/A	Mild Bite and touch	N/A	24 hours	N/A	N/A	<0.0001	N/A	The most frequently reported pain type at baseline was ‘pressure,’ and at 24 hours it was ‘bite and touch’; both were classified as mild.
	Diddige et al (2019), India	VAS	0 to 10 cm	4h, 24h, 3d, 1w	NR	NR	2.72	0.71	24 hours	2.72	0.71	0.001	N/A	Peak of pain in the first 24 hours with values of 2.72(SD 0.71).
	Fujiyama et al (2014), Japan	VAS	0 to 10 cm	Baseline, 6h, 12h 24h, 2d, 3d, 4d, 5d, 6d, 1w	23.17	33.45	36.55	61.40	24 hours	13.38	33.45	<0.05	57.75%	Increases 57.75% at first 24 hours.
	Gao et al (2021), China	VAS	0 to 100 mm	24h, 2d, 3d, 4d, 5d, 6d, 1w, 2w	NR	NR	29.97	33.11	24 hours	N/A	N/A	< 0.05	N/A	Peak of pain in the first 24 hours with values of 29.97(SD 33.11).
	Mertoglu et al (2025). Turkey	VAS/5-question survey	0 to 100 mm	Baseline. 4h. 24h. 1w. 2w. 1m. 3m. 6m	1.63	3.50	16.39	10.66	24 hours	7.16	9.41	<0.001	905.5%	Pain increased by 905.5% within 24 hours compared to baseline.
	Rucker et al (2012), United States	VAS	10 bubble	24h, 1w, 2w, 3w	NR	NR	8,83	1,15	2 weeks	N/A	N/A	0,084	N/A	Pain of 8.83 (SD 1.15) after 2 weeks (insertion of new aligners).
	Zhao et al (2023), China	VAS	0 to 10 cm	24h, 2d, 3d, 1w	NR	NR	2,50	1,72	24 hours	2,67	0,31	>0,05	N/A	Peak pain perception is 2.50 (SD 1.72) within the first 24 hours.
Plaque	Selvakamur et al (2024), India	Plaque Index	0–3	Baseline, 3m, 6m	1,00	0,30	1,70	0,40	6 months	0,70	0,77	0,001	70,00%	A increase of 70% of plaque.
	Levrini et al (2013), Italy	Silness&Loe	0–3	Baseline, 1m, 3m	0,50	0,51	0,40	0,59	3 months	−0,10	0,77	<0,001	−0,2	A decrease of 20% of plaque.
	Albhaisi et al (2020), Jordan	QLF (red)	%	Baseline, 3m	0,50	0,80	1,70	2,40	3 months	1,20	2,45	0,148	2,4	Increase of 240% in plaque.
	Mertoglu et al (2025), Turkey	Plaque Index	Silness&Loe (Ramfjord teeth 16)	Baseline, 3m, 6m		N/R	N/R	N/R	6 months	0,323*	0,208*	<0.001	N/A	An increase of 0.323 (SD = 0.21) in plaque index was observed over 6 months.
Periodontal health status	Levrini et al (2013), Italy	Bleeding on probing	20 seconds after probing (absent = 0, present = 1)	Baseline, 1m, 3m	0,25	0,44	0,40	0,50	3 months	0,15	0,67	<0,001	60,00%	An increase of 60% of bleeding on probing.
		Pocket probing depth	mm	Baseline, 1m, 3m	2,00	0,00	2,30	0,47	3 months	0,30	2,05	0,002	15,00%	An increase of 15% of pocket probing depth.
	Almagrami et al (2023), China	CBCT	Bone Height (mm)	Baseline, 26m	2,84	1,81	4,85	3,88	26 months	2,01	4,28	0,160	70,77%	Increase of 70% in the distance between the crest of the alveolar ridge and CEJ on the labial and palatal surfaces, considering a decrease in bone height.
		CBCT	Bone Thickness (mm)	Baseline, 26m	0,95	0,46	0,86	0,66	26 months	−0,09	0,80	0,000	−9,47%	Decrease of 9,47% in thickness of alveolar bone.
	Selvakumar et al (2024), India	Gingival Index	0-3	Baseline, 3m, 6m	0,90	0,20	1,30	0,30	6 months	0,40	0,26	0,001	44,44%	Increase of 44,44% of gingival index after 6 months.
		Probing Depth	0-3	Baseline, 3m, 6m	2,50	0,40	2,80	0,40	6 months	0,30	0,40	0,001	12,00%	Increase of 12% of probing depth after 6 months.
	Mahayyudin et al (2024), China	N/R	Alveolar bone density (HU)	Baseline, 6 months, 18 months	950,00	41,09*	890,00	56,864*	18 months	−60,00	61,28*	0,450	−6,32%	Decrease of 60.00 units (–6.32%) over 18 months.
		N/R	Alveolar bone volume (mm3)	Baseline, 6 months, 18 months	0,25	0,07*	0,22	0,053*	18 months	−0,03	0,063*	0,020	−12,00%	Decrease of 0,03mm3 (–12%) over 18 months.
	Mertoglu et al (2025), Turkey	Bleeding on probing (Ramfjord teeth – 16)		Baseline, 3m, 6m	N/R	N/R	N/R	N/R	6 months	0,32	0,21	<0.001	N/A	Bleeding on probing increased by 0.32 over a 6-month period.
		Probing depth (Ramfjord teeth – 16)		Baseline, 3m, 6m	N/R	N/R	N/R	N/R	6 months	0,10	0,13	0,020	N/A	Probing depth increased by 0.10 mm over a 6-month period.
	Huo et al (2024), China	CBCT	Bone Thickness (mm)	Baseline, 12m	N/R	N/R	N/R	N/R	12 months	0,09	0,02	<0,000	N/A	Decrease of 0,09 mm (SD = 0,02) in thickness of alveolar bone.
			Bone Height (mm)	Baseline, 12m	N/R	N/R	N/R	N/R	12 months	1,03	0,17	0,001	N/A	Decrease of 1,03 mm (SD = 0,17) in height of alveolar bone.
	Zhang et al (2025), China	Gingival Index in adults	0-3	Baseline, 6m	1,19	0,17	1,42	0,21	6 months	0,23	0,27	0,958	19,30%	Gingivitis worsened over 6 months, with a 19,3% increase in the mean inflammation score.
		Gingival Index in adolescents	0-3	Baseline, 6m	0,15	0,07	0,59	0,19	6 months	0,44	0,20	0,035	293%	Gingivitis worsened over 6 months, with a 293% increase in the mean inflammation score.
		Incidence (Gingivitis) in adults	%	Baseline, 6m	N/R	N/R	70,04	N/A	6 months	N/A	N/A	0,050	N/A	After 6 months, 70,04% of adult patients developed gingivitis.
		Incidence (Gingivitis) in adolescents	%	Baseline, 6m	N/R	N/R	39,39	N/A	7 months	N/A	N/A	0,050	N/A	After 6 months, 39,39% of adolescents patients developed gingivitis.
Periodontal pain	Alcón et al (2021), Spain	VAS	0 to 10 cm (overall)	1m, 2m, 3m, 4m, 5m, 6m, 7m, 8m, 9m, 10m, 11m, 12m	NR	NR	1,98	2,30	1 month	1,98	2,30	0.,047	N/A	Peak of periodontal pain in the 1st month with values of 1,98 (SD 2,30).
	Antonio-Zancajo et al (2021), Spain	Questionnaire of intensity Total	moderate (%)	4h, 8h, 24h, 2d, 3d, 4d, 5d, 6d, 1w	NR	NR	53,30	N/A	24 hours	N/A	N/A	0,120	N/A	The degree of pain was moderate at 53,30% of cases at first 24 hours.
		Short-Form McGill Pain Questionnaire	both archs (%)	4h, 8h, 24h, 2d, 3d, 4d, 5d, 6d, 1w	NR	NR	100,00	N/A	24 hours	N/A	N/A	0,001	N/A	Periodontal pain frequently affected both archs at first 24 hours.
		Questionnaire of type of pain	sensitive (%)	4h, 8h, 24h, 2d, 3d, 4d, 5d, 6d, 1w	NR	NR	36,70	N/A	24 hours	N/A	N/A	0,002	N/A	The most frequent periodontal type of pain was sensitive at first 24 hours.
ARR	Al-Dboush et al (2022), Canada	CBCT	Volumetric upper right central incisor (cm3)	Baseline, 26m	236,24	48,34	223,28	47,12	26 months	−12,96	67,51	<0,001	−5,49%	Root volume decreased by 5.49%.
	Almagrami et al (2023), China	CBCT	Apical (mm)	Baseline, 26m	12,72	1,24	12,38	1,37	26 months	−0,34	1,85	0,000	−2,67%	A decrease in root length by 2.67%.
	Eissa et al (2018), Canada	CBCT	Apical upper right central incisor (mm)	Baseline, 18m	23,35	1,79	22,84	1,74	18 months	−0,50	2,50	0,003	−2,18%	A decrease in root length by 2,18%.
	Huo et al (2024), China	CBCT	Upper central incisor (mm)	Baseline, 12m	N/R	N/R	0,85	0,17	12 months	−0,85	0,17	0,001	N/A	Decrease of 0,85 mm (SD 0,17) in root length.
	Khalil et al (2023), Egypt	CBCT	Apical lower right central incisor (mm3)	Baseline, 6m	NR	NR	−6,20	2,94	6 months	−6,20	2,94	0,530	N/A	Decrease of 6.20 mm3 (SD 2.94) in root length.
	Mahayyudin et al (2024), China	N/R	N/R	Baseline, 6m, 18m	14,50	0,921*	13,08	1,432*	18 months	−1,42	1,26*	0,010	9,79%	A decrease in root length by 9,79%.
		Clinically Significant ARR (>2 mm)	%	18m	N/A	N/A	7,69	N/A	18 months	N/A	N/A	0,020	N/A	7,69% experienced significant ARR.
	Mertoglu et al (2025), Turkey	Periapical radiographs	Upper Light central incisor (mm)	Baseline, 3m, 6m	29,12	3,12	28,31	3,26	6 months	−0,82	0,95	<0.001	−2,78%	A decrease in root length by 2,78%.
	Rossi et al (2024), Canada	CBCT	Upper left central incisor (mm3)	Baseline, 24m	493,89	100,57	N/A	N/A	24 months	−2,11	16,95	0,733	−0,43%	The greatest root volume reduction over 24 months was approximately 0.43%.
	Wang et al (2017), China	Prevalence	%	6m, 12m	NR	NR	68,30	N/A	12 months	N/A	N/A	<0,050	N/A	Prevalence of 68.3%.
	Withayanukonkij et al (2023), Thailand	CBCT	mm	Baseline, 6m	20,60	0,87	20,36	0,90	6 months	−0,24	1,25	<0,001	−1,17%	A decrease in root length by 1,17%.
Speech Changes	Damasceno Melo et al (2020), Brazil	VAS	0 to 10 cm	After, 3d, 1m, 6m	NR	NR	2,07	2,47	After	2,07	2,47	0,301	N/A	It interfered with speech, with an average value of 2.07 (SD = 2.47).
		MBGR	%	After, 3d, 1m, 6m	NR	NR	17,00	N/A	After	N/A	N/A	<0,001	N/A	Changes in speech at the beginning of treatment in 17% of cases.
	Fraundorf & Kim (2020), United States	Digital sonography	Upper boundary frequency (UBF; the maximum frequency ofthe band width of the fricative) of the middle 50 ms ofthe /s/ sound.	Baseline, After, 2m	11,68	0,46	11,360	0,50	After	−0,32	0,68	0,640	−2,74%	Speech is altered by 2.74% as the tongue’s contact area shifts due to the appliance on the lingual surface.
		Patient Questionnaire	Likert scale: 1–4	Baseline, After, 2m	1,08	0,27	1,58	0,65	After	0,50	0,70	0,400	46,30%	Increases 46,30% deterioration in articulation immediately after appliance delivery
		Six certified speech and language pathologis	Frequencie of speech alteration (%)	Baseline, After, 2m	NR	NR	48,60	N/A	After	N/A	N/A	NR	N/A	Midlly to moderate speech alteration with a frequencie of 48,60% of cases.
Temporomandibular joint disorders	Alam et al (2024), Saudi Arabia	Incidence (RDC/TMD)	%	Baseline, 6m, 12m	10,00	N/A	14,00	N/A	12 months	N/A	N/A	0,03	40,00%	There was a 40% increase in the number of patients with temporomandibular joint disorders after 12 months.
		Severity (RDC/TMD)	Mild to Moderate	12m	N/A	N/A	100,00	N/A	12 months	N/A	N/A	0,67	N/A	Most patients had mild (71%) and some had moderate (29%) symptoms
White spot lesions	Albhaisi et al (2020), Jordan	QLF	%	Baseline, 3m	67,00	92,60	149,20	183,40	3 months	82,20	195,26	<0,001	122,69%	Increase of 122,69% in white spot lesion.
	Liu & Song (2024), China	Incidence	% (pre WSLs)	Baseline, 24m	31,30	N/A	35,50	N/A	24 months	4,20	N/A	< 0,001	13,42%	Pre-existing WSLs were significantly associated with a 13.42% increase in post-treatment lesions.
	Buschang et al (2019), United States	Frequencie	% (pre-existing WSL that developed more WSL)	Baseline, 18m	NR	NR	84,10	N/A	18 months	N/A	N/A	0,001	N/A	84,10% of cases pre-existing WSL developed more WSL.

### Certainty of evidence

Table 5 presents, for each outcome, the effect estimates (absolute and/or relative), the number of studies and participants, and the certainty of the evidence (high, moderate, low, or very low) assessed using the GRADE approach. Certainty ratings were based on the five GRADE domains: risk of bias, inconsistency, indirectness, imprecision, and publication bias. Explicit justifications for each decision to downgrade or upgrade the certainty are provided in the “Reasons for rating decisions” column. When quantitative synthesis was feasible, certainty assessments were informed by the meta-analysis; otherwise, they were based on the narrative synthesis of the body of evidence.

**Table 5:** Summary of findings.

Outcome	№ of studies Study design (n)	Effect (95% CI) Tool	Certainty of the evidence (GRADE)	Comments/Reasons for rating
**Pain** **24h**	10 studies 3 RCT, 2 NRCT, 5 Cohort (418)	Mean 3.06 (2.15 to 4.37) VAS 0–10	⨁◯◯◯ **Very low**	**Risk of bias:** Most studies were NRS with self-reported outcomes and a lack of blinding. **Inconsistency:** High heterogeneity (I² > 80%) and variability in effect sizes across studies. **Indirectness:** Populations, interventions, and outcomes were directly relevant. **Imprecision:** Wide CIs and small sample sizes reduce certainty in the true effect. **Publication bias:** Few small studies with unclear risk of selective reporting.
**Pain** **3 days**	7 studies 3 RCT, 1 NRCT, 3 Cohort (259)	Mean 1.55 (1.09 to 2.21) VAS 0–10	⨁⨁◯◯ **Low**	**Risk of bias:** Evidence included RCTs and NRS; downgraded due to concerns in non-randomized designs (confounding, lack of blinding, self-reported outcomes). **Inconsistency:** High heterogeneity (I² > 80%) with variable effect sizes. **Indirectness:** Not serious; evidence directly applicable. **Imprecision:** Not serious; relatively narrow confidence interval. **Publication bias:** Possible due to small number of studies and inability to formally assess.
**Pain** **1 week**	6 studies 2 RCT, 1 NRCT, 3 Cohort (229)	Mean 1.08 (0.33 to 3.51) VAS 0–10	⨁◯◯◯ **Very low**	**Risk of bias:** Evidence included RCTs and NRS; downgraded due to concerns in non-randomized designs (confounding, lack of blinding, self-reported outcomes). **Inconsistency:** High heterogeneity (I² > 80%) with variable effect sizes. **Indirectness:** Not serious; evidence directly applicable. **Imprecision:** Serious; wide confidence interval indicating substantial uncertainty. **Publication bias:** Possible due to small number of studies and inability to formally assess.
**Plaque**	3 Studies 3 RCT (87) 1 Study RCT (27)	Mean 0,23 (−0,49–0,95) Silness&Löe Mean 1,70 (SD = 2,40) (QLF red)	⨁⨁◯◯ **Low**	**Risk of bias:** Four small RCTs with unavoidable lack of blinding and observer-dependent plaque indices. **Inconsistency:** All studies consistently favored aligners for less plaque. **Indirectness:** Populations and plaque measures (PI, DR30, microbiology) appropriate. **Imprecision:** Despite pooling, the 95% CI is wide and crosses the null and the minimal clinically important difference, indicating serious imprecision. **Publication bias:** Few small studies, no clear reporting bias.
**ARR** **6 months**	3 studies 2 RCT, 1 Cohort (125)	Mean −0.29 mm (−0.45 to −0.05) CBCT/periapical radiograph	⨁⨁⨁◯ **Moderate**	**Risk of bias:** Evidence included RCTs and cohort studies; some concerns due to non-randomized designs and measurement variability. **Inconsistency:** Not serious; effect estimates were relatively consistent across studies. **Indirectness:** Not serious; populations and outcome measures were clinically appropriate. **Imprecision:** Not serious; confidence interval relatively narrow and does not include no effect. **Publication bias:** Possible but not detected; limited number of studies.
**ARR** **End of treatment**	6 studies 2 RCT, 1 NRCT, 3 Cohort (216)	Mean −0.33 mm (−0.55 to −0.11) CBCT	⨁⨁◯◯ **Low**	**Risk of bias:** Evidence included RCTs and NRS; downgraded due to concerns in non-randomized designs and potential confounding. **Inconsistency:** Some heterogeneity across studies and timepoints. **Indirectness:** Not serious; evidence directly applicable. **Imprecision:** Not serious; confidence interval relatively consistent in direction of effect. **Publication bias:** Possible due to limited number of studies.
**Volumetric ARR**	3 studies 1 NRCT, 2 Cohort (47)	Mean −4.37 mm³ (−5.51 to −3.24) CBCT	⨁⨁⨁◯ **Moderate**	**Risk of bias:** Evidence based on non-randomized and cohort studies; downgraded due to potential confounding and selection bias. **Inconsistency:** Not serious; effect estimates were consistent across studies. **Indirectness:** Not serious; CBCT measures directly relevant. **Imprecision:** Not serious; confidence interval relatively narrow and precise. **Publication bias:** Possible due to small number of studies.
**Awake bruxism**	1 study RCT (19)	Frequency 53,5% EMA	⨁⨁◯◯ **Low**	**Risk of bias:** Lack of blinding, reliance on self-reported EMA with partial compliance, and small single-arm sample raise concerns about bias. **Inconsistency:** Only one study available, with consistent within-study results and no conflicting directions. **Indirectness:** Population, intervention, and outcome are directly relevant, though applicability beyond young adults may be limited. **Imprecision:** Small sample size and wide variability limit confidence in the true effect estimate. **Publication bias:** Evidence is limited to a single small study, with no clear signals of selective reporting.
**Caries / Fillings**	1 Study Cohort (25)	DMFT: + 0.52 (ICC = 0.88) Bite-wing radiograph	⨁⨁◯◯ **Low**	**Risk of bias:** Comparative cohort with small sample increases confounding risk. **Inconsistency:** Results were consistent within study comparisons. **Indirectness:** Population and DMFT outcomes directly address caries risk. **Imprecision:** Limited by n = 25 per group and short-term follow-up (6m). **Publication bias:** Only one study, no evidence of selective reporting.
**Chewing Performance**	1 Study RCT (27)	Mean 19.56 (SD = 4.63) VAS 1–100	⨁⨁◯◯ **Low**	**Risk of bias:** RCT but lack of blinding and subjective VAS outcomes. **Inconsistency:** Within-study results were coherent, no conflicting trends. **Indirectness:** Outcomes directly relevant to functional chewing. **Imprecision:** Small sample per arm limits certainty about effect size. **Publication bias:** Single RCT, no selective reporting signals.
**Enamel Demineralization**	1 Study RCT (19)	Mean ↑ 82px QLF (lesion area)	⨁⨁◯◯ **Low**	**Risk of bias:** RCT with short follow-up and limited blinding of participants. **Inconsistency:** One study, internally consistent findings. **Indirectness:** Population and QLF outcomes directly relevant. **Imprecision:** Small n and narrow timeframe limit confidence. **Publication bias:** Single small RCT, no reporting bias identified.
**Halitosis**	1 Study NRS (31)	Mean 42.46 (SD = 14.78) Halimeter® (ppb)	⨁⨁◯◯ **Low**	**Risk of bias: NRS** Crossover design without control and unblinded participants. **Inconsistency:** Single study with coherent within-study results. **Indirectness:** Outcomes (Halimeter, organoleptic index) directly measure halitosis. **Imprecision:** n = 31 and very few events limit certainty of effect. **Publication bias:** One study, no evidence of selective reporting.
**Open Gingival Embrasures**	1 Study Cohort (100)	Incidence 35–38% (maxillar-mandibular) Photograph	⨁⨁◯◯ **Low**	**Risk of bias:** Retrospective cohort with possible confounding and selection bias. **Inconsistency:** Findings consistent within study. **Indirectness:** Outcome and population relevant, though limited to mild crowding. **Imprecision:** n = 100 in Aligner arm yields wide CIs for incidence estimates. **Publication bias:** Only one observational study available.
**Periodontal health status**	7 Studies 3 RCT, 4 Cohort (311)	Worsening gingival inflammatory indicators (BOP, GI, PD) over 6 months and a clinically relevant loss of alveolar bone height, thickness, and volume within up to 18 months. CBCT, Bleeding on probing, Gingival Index, Pocket probing depth	⨁⨁◯◯ **Low**	**Risk of bias:** Combination of RCT and cohorts with confounding risk. **Inconsistency:** Direction of effect unclear across studies. **Indirectness:** Populations, interventions, and periodontal measures directly relevant. **Imprecision:** Modest sample sizes and ≤6m follow-up limit certainty. **Publication bias:** Few studies, unclear small-study effects.
**Speech changes**	2 Studies 1 RCT, 1 Cohort (84)	Early, mild-to-moderate and partly measurable speech disturbances, which are most pronounced immediately after insertion and tend to diminish with adaptation. VAS, MBGR, Digital Sonography, Patient Questionnaire, Six certified speech and language pathologies	⨁◯◯◯ **Very low**	**Risk of bias:** Small RCT and cohort, both unblinded with subjective and objective measures. **Inconsistency:** Discrepancy in recovery time (30d vs persistence at 2m). **Indirectness:** Populations and speech outcomes directly relevant. **Imprecision:** n = 20–24 in CAT arms, small event counts. **Publication bias:** Very few studies, no selective reporting signs.
**Temporomandibular disorders**	1 Study Cohort (50)	Incidence 14%, mostly mild/moderate; no association with Clear Aligners. Incidence and Severity (RDC/TMD)	⨁⨁◯◯ **Low**	**Risk of bias:** Cohort with small aligner arm and possible confounding. **Inconsistency:** Only one study, internally consistent results. **Indirectness:** Adolescents studied, applicability to adults uncertain. **Imprecision:** Small with only 7 events, wide CI. **Publication bias:** Only one cohort available, unclear bias.
**White spot lesion**	3 Studies 2 RCT, 1 Cohort (474)	Substantial increase in WSL occurrence, especially when pre-existing lesions are present. QLF, Incidence and Frequence	⨁◯◯◯ **Very low**	**Risk of bias:** Evidence includes retrospective cohorts and two small RCTs. **Inconsistency:** Large variation in incidence across studies. **Indirectness:** Differences in age, methods (photos vs QLF), and follow-up. **Imprecision:** Small samples and few events in some cohorts. **Publication bias:** Limited studies, no reporting bias detected.

## Discussion

This SR identified several adverse effects associated with removable clear aligners, many of which also observed with fixed appliances and should be considered during treatment planning and informed consent. Pain is expected at treatment initiation and is generally mild–moderate, peaking at 24–48 h, decreasing by day 3, and reaching minimal levels by week one. Differences across studies largely reflect variation in measurement instruments (0–100 mm vs 0–10 cm VAS) and assessment methods. The removability of clear aligners may also influence perceived discomfort [47, 64, 65]. Our meta-analyses confirmed the temporal pattern, with pain highest at 24 h, lower by day 3, and minimal by week one, although heterogeneity was very high (I² ≈ 83–99%). This level of heterogeneity is expected in single-arm syntheses and supports the need to explore, rather than exclude, variability [41]. The high heterogeneity observed across several analyses should be interpreted with caution but is not unexpected in SR of adverse effects, where variability in outcome definitions, measurement instruments, treatment protocols, and follow-up duration is common [66]. Methodological guidance indicates that statistical heterogeneity alone does not preclude meta-analysis when studies address a common clinical question; in such cases, random-effects models can be used to account for between-study variability [41]. Accordingly, pooled estimates should be interpreted as average effects across heterogeneous clinical contexts rather than as precise treatment effects. Risk of bias was generally low among RCTs, although one trial was rated as high risk. Non-randomized studies ranged from moderate to critical risk, primarily due to confounding and selection bias [11, 46, 47, 52, 59]. The use of standardized pain measures and longer follow-up periods is warranted to improve comparability and strengthen the evidence base [11, 51, 65, 67].

Changes in plaque were small, with wide confidence intervals crossing the null and inconsistent directions across studies and measurement approaches (whole-mouth assessments vs Ramfjord sites), resulting in very high heterogeneity (I² = 95–96%) and low certainty of evidence. Periodontal pain tended to peak at 24 hours, particularly in the mandibular arch [11, 67].

Although ARR remains a concern, particularly in the maxillary incisors, pooled estimates indicated a minimal mean reduction by the end of treatment (approximately −0.33 mm; 95% CI −0.55 to −0.11), with small volumetric losses. The magnitude of these changes is unlikely to be clinically significant over the long term [14, 15, 68, 69]. Variability appears to be related to factors such as age, sex, malocclusion, bone density, treatment duration, and biomechanics, as well as differences in outcome assessment methods [15]. While some studies relied on linear measurements obtained from periapical radiographs or CBCT-derived lengths, others used volumetric analyses based on three-dimensional segmentation, which may provide a more comprehensive evaluation of structural changes. These methodological differences limit direct comparability and may contribute to heterogeneity in the pooled estimates. CBCT-based assessments provide higher sensitivity for volumetric root loss and alveolar bone changes and should be considered when available for precise quantification [70]. Study quality ranged from low to high risk of bias across designs. Current guidance supports the use of light forces and staged tooth movements, with caution during intrusion and rotational movements [14, 15, 56, 59, 71].

Aligner therapy was primarily associated with mild TMD symptoms, with no reports of severe manifestations. However, one study was assessed as having a critical risk of bias (selection, confounding, and reporting), which limits confidence in these findings despite a low risk of bias in outcome measurement [72, 73].

One RCT assessing AB found no difference between aligners and fixed appliances; an initial decrease returned to baseline at six months (low risk across RoB 2 domains) (59). Other studies have reported a higher prevalence of AB among aligner users, highlighting the need for individual risk assessment [74].

Aligners were associated with increased occurrence of OGEs in one study (overall moderate risk; low risk in key domains), with supporting external evidence indicating notable incidence in anterior teeth. Interproximal reduction may help prevent OGEs by repositioning the contact point apically [22, 75].

Three studies reported buccal bone reduction (−0.80 to −3.05 mm; 6.3–12% volume/density), which may increase the risk of alveolar bone dehiscence or fenestration in susceptible patients. These defects may compromise periodontal stability and predispose teeth to gingival recession when orthodontic movement exceeds the biological limits of the alveolar bone housing. Careful treatment planning is therefore essential, particularly in cases involving dental expansion or significant buccal tooth movement. Assessment of the patient’s periodontal phenotype, control of orthodontic force magnitude, and individualized biomechanics should be considered to minimize the risk of alveolar bone defects. When clinically indicated, imaging modalities such as cone-beam computed tomography may aid in evaluating the buccal bone plate and guiding safer treatment decisions. The broader orthodontic literature reports a higher prevalence of buccal dehiscence and fenestrations associated with no-extractions expansion, particularly in patients with thin periodontal phenotypes or limited alveolar bone support [76–78], supporting the need for preventive planning and periodontal monitoring during treatment [16, 17, 44, 79]. The risk of bias across these studies was mixed, with cohort designs generally presenting moderate risk or some concerns, warranting cautious interpretation of the findings [16, 44, 62].

Transient speech alterations (e.g., /ch/, /s/, /z/) were frequently reported, with recovery typically occurring within 1–3 months, although longer adaptation periods may be observed in indivuduals with speech-sensitive professions. Referral may be beneficial in persistent cases [23, 61, 80, 81]. Methodological quality varied across studies with RCT generally at low risk of bias and cohort studies at moderate risk) [23, 61].

Evidence suggests that WSLs may increase with aligner therapy, particularly in the presence of suboptimal oral hygiene, pre-existing lesions, or attachments. Although some comparisons with fixed appliances report a lower incidence, lesions may be larger and less mineralized. Overall, findings are inconsistent and of very low to low certainty [18–21, 57, 82].

Perceptions of halitosis and dry mouth may increase transiently during the first months of aligner therapy, particularly within the first 3–4 months, with minimal impact on volatile sulfur compounds (VSCs) or inflammatory parameters and only temporary benefit from chlorhexidine; oral hygiene remains a key factor [24]. The cohort study addressing halitosis presented a serious risk of bias, primarily due to confounding and participant selection, which limits the reliability of its findings [24]. These short-term perceptions are generally mild and tend to decrease over time [24, 54]. Evidence regarding longer-term changes remains limited; however, available data suggest that these effects may stabilize during treatment. Persistent alterations may occur in the presence of inadequate oral hygiene or sustained biofilm accumulation on aligner surfaces. Orthodontic appliances, including thermoplastic aligners, can modify oral microbial ecology and plaque accumulation patterns, partly due to surface irregularities and microabrasions that facilitate bacterial adhesion and biofilm formation [82, 83]. These biofilms may contribute to oral malodor when hygiene is suboptimal, as demonstrated by studies reporting bacterial colonization of removable orthodontic appliances [84, 85]. Together, these findings support the importance of strict oral and aligner hygiene to minimize both short- and longer-term adverse effects.

Many adverse effects are minor and/or transient. Clinicians should assess individual risks factors (e.g., bruxism, susceptibility to ARR), monitor bone and periodontal health (with imaging when indicated), provide guidance on pain and speech adaptation, consider IPR to mitigate OGEs, and reinforce strict oral hygiene to reduce the risk of WSLs, halitosis, and related complications. These clinical considerations are consistent with general orthodontic practice principles, although the present review did not include direct comparisons with fixed appliances. Based on the findings of this review, the following clinical considerations may assist in interpreting potential adverse effects in practice (Table 6).

**Table 6:** Clinical considerations based on the synthesized evidence. Legend: AB – Awake Bruxism. DMFT – Decay, Missing, Filled Teeth Index. VAS – Visual Analogue Scale. QLF – Quantitative Light-induced Fluorescence. WSL: White Spot Lesion. VSC: Volatile Sulfur Compound. TMJ – Temporomandibular Joint.

Adverse Effect	Key Finding	Clinical Considerations
Awake Bruxism	Frequency increased slightly by 0.18% after appliance placement (55.9% to 56.0%), with light tooth contact being the most common behavior, followed by clenching.	The prevalence of awake bruxism remained stable with aligner use, suggesting that these appliances neither reduce nor aggravate the behavior. In patients with pre-existing bruxism, careful monitoring is recommended, particularly for light tooth contact and clenching, given their potential impact on tooth wear and treatment outcomes [58].
Caries/filling	Increase of 9,92% in the number of DMFT Index after 6 months of treatment.	Caries risk is not unique to aligners, but prolonged wear and insufficient tray hygiene may exacerbate it. Proper cleaning of the appliances and regular monitoring are essential, particularly for high-risk patients [20].
Discomfort	Pain levels initially reached 19.36 mm (SD = 22.51) on a VAS (0–100 mm), increasing by 79.93% before decreasing to 12.60 mm (SD = 11.33) within a few days.	Initial discomfort with aligners is like that with brackets, typically mild to moderate and transient. Temporary relief may be achieved by removal, but continuous wear is essential to ensure treatment efficacy [52].
Enamel changes	The surface area of WSLs increased by 122.69%, while QLF loss, a marker of enamel demineralization, grew by 5.48% over 3 months, indicating progressive enamel deterioration. At 18 months, the frequency of WSLs reached 1.2% across both arches, and 84.1% of patients with pre-existing lesions developed additional ones. Moreover, pre-existing WSLs were significantly associated with a 13.42% increase in new post-treatment lesions.	Preventive strategies, including strict oral hygiene and regular fluoride varnish application, to minimize risks and support patient care [18, 19].
Halitosis [46]	VSC levels measured by Halimeter® ranged from 42.27 to 46.77 ppb (SD = 16.58 to 24.29). Within normal limits.	No clinically significant halitosis was observed with aligner use, but strict oral and tray hygiene is essential to prevent odor [24].
Open gingival embrasures	Photographic analysis at 26 months showed an average area of 0.21 mm² (SD = 0.24) in the mandible and 0.16 mm² (SD = 0.12) in the maxilla, with an incidence of 36.5%.	IPR may reduce its degree by relocating the contact point more gingival [22].
Pain	The most common types of pain shifted from “pressure” at baseline to “bite and touch” at 24 hours, both mild in intensity.	Aligner-related pain was mild and transient, shifting from initial pressure to biting discomfort, and is generally perceived as more manageable than with brackets [53, 60].
Periodontal health	Increased bleeding on probing (up to 60%), greater probing depth (up to 15%), worsened gingival inflammation (up to 293%), and reduced alveolar bone height and thickness (up to 12%). Gingivitis developed in 70% of adults and 39% of adolescents within six months.	Increased alveolar dehiscences and fenestrations from dental expansion in moderate crowding highlight the need for preventive treatment planning to consider alveolar bone integrity [16, 17, 44, 54, 59].
Periodontal pain	Moderate pain was reported by 53.3% of patients, while 100% experienced pain in both arches. Additionally, 36.7% reported sensitive pain.	Periodontal pain is frequent at the start of aligner therapy, often moderate but transient, and patient counseling is essential to set expectations and emphasize that symptoms usually decrease over time [11, 55].
Plaque	Reported a significant increase, up to 240% over six months.	This variability highlights the importance of reinforcing preventive strategies, including strict oral and aligner hygiene, to minimize risks and support patient care [18].
ARR	The prevalence reached 68.3%, though most cases were mild, with significant resorption reported in 7.69% of patients.	During orthodontic treatment, light forces and staged movements are recommended, with caution advised for intrusion and rotation [45, 62].
Speech changes	85% of participants reported speech changes, which were reduced after 3 days. The /s/ sound was most affected (17% of cases), 2.74% reported tongue contact with aligners, and 46.3% experienced speech deterioration. 48.6% had mild to moderate impairment.	Speech changes are common in the first days of aligner use, usually mild and transient; referral to speech therapy may be helpful in persistent cases [23, 61].
Temporomandibular joint disorders	After 12 months of aligner treatment, there was a 40% increase in the number of patients with temporomandibular joint disorders, with most presenting mild (71%) and some moderate (29%) symptoms.	TMD symptoms may increase during aligner therapy, usually mild to moderate; as this is a multifactorial condition, findings should be interpreted with caution and TMJ function monitored, particularly in at-risk patients [24].

### Limitations

Substantial heterogeneity in measures, study populations, and follow-up durations, along with incomplete representation of malocclusion complexity, limits comparability and generalizability. Standardized methodologies and consistent follow-ups protocols are needed to strengthen the evidence base.

## Conclusion

The main adverse effects of clear aligners include pain and discomfort, typically most intense during the first 24–48 hours, and mild ARR, particularly in incisors. Less frequently reported but clinically relevant effects include enamel demineralization, white spot lesions, caries in areas with poor hygiene, open gingival embrasures after prolonged treatment, and mild, transient speech alterations. Periodontal health compromise is generally mild. Bruxism and temporomandibular disorders, though often mild to moderate, remain a concern. Although this review analyzed aligner arms only and direct comparisons with fixed appliances cannot be made, evidence from randomized trials comparing aligners and fixed appliances provides important context for interpreting patient-reported outcomes and adverse effects.

## Supporting information

S1 Checklist PRISMA checklist. (DOCX)

S2 Checklist SWiM checklist. (DOCX)

S3 Table Search strategy. (DOCX)

S4 AI Risk of Bias Prompts. (DOCX)

S5 Outcome harmonization procedures and worked examples. (DOCX)

S6 Assessment of selective reporting and completeness of adverse effects. (DOCX)

S7 Excluded studies and reasons for exclusion. (DOCX)

S8 R Scripts. (DOCX)

## Abbreviations

AB: Awake bruxism
AI: Artificial intelligence
BVS: Biblioteca Virtual em Saúde
CA: Clear aligner
CBCT: Cone beam computed tomography
CI: Confidence interval
DC/TMD: Diagnostic Criteria for Temporomandibular Disorders
GRADE: Grading of Recommendations Assessment, Development and Evaluation
IPR: Interproximal enamel reduction
LILACS: Literatura Latinoamericana y del Caribe en Ciencias de la Salud
MA: Meta-analysis
NRS: Non-randomized studies (study without random allocation)
OHIP-14: Oral Health Impact Profile (14-item version)
OGE: Open gingival embrasure
PRISMA: Preferred Reporting Items for Systematic Reviews and Meta-Analyses
PRISMA-P: Preferred Reporting Items for Systematic Reviews and Meta-Analyses Protocols
PROSPERO: International prospective register of systematic reviews
QLF: Quantitative Light-induced Fluorescence
RDC/TMD: Research Diagnostic Criteria for Temporomandibular Disorders
RCT: Randomized controlled trial
RoB 2: Risk of Bias 2 tool for randomized trials
ROBINS-I: Risk Of Bias In Non-randomized Studies – of Interventions
SD: Standard deviation
SR: Systematic review
SWiM: Synthesis Without Meta-analysis
VAS: Visual Analog Scale
TMD: temporomandibular disorders
TMJ: temporomandibular joint
VSC: Volatile sulfur compound
WSL: White spot lesion

## References

[1] Phan X, Ling PH. Clinical limitations of Invisalign. J Can Dent Assoc. 2007;73(3):263–6. 17439714

[2] Weir T. Clear aligners in orthodontic treatment. Aust Dent J. 2017;62 Suppl 1:58–62. doi: 10.1111/adj.12480 28297094

[3] Allareddy V, Nalliah R, Lee MK, Rampa S, Allareddy V. Adverse clinical events reported during Invisalign treatment: analysis of the MAUDE database. Am J Orthod Dentofacial Orthop. 2017;152(5):706–10. doi: 10.1016/j.ajodo.2017.06.014 29103448

[4] Talic NF. Adverse effects of orthodontic treatment: a clinical perspective. Saudi Dent J. 2011;23(2):55–9. doi: 10.1016/j.sdentj.2011.01.003 24151415 PMC3770235

[5] Janson GR, De Luca Canto G, Martins DR, Henriques JF, De Freitas MR. A radiographic comparison of apical root resorption after orthodontic treatment with 3 different fixed appliance techniques. Am J Orthod Dentofacial Orthop. 2000;118(3):262–73. doi: 10.1067/mod.2000.99136 10982926

[6] Yassir YA, McIntyre GT, Bearn DR. Orthodontic treatment and root resorption: an overview of systematic reviews. Eur J Orthod. 2021;43(4):442–56. doi: 10.1093/ejo/cjaa058 33215186

[7] Sadowsky C, BeGole EA. Long-term effects of orthodontic treatment on periodontal health. Am J Orthod. 1981;80(2):156–72. doi: 10.1016/0002-9416(81)90216-5 6943936

[8] Alotaibi S. Potential side effects of comprehensive fixed orthodontic treatment: a narrative review. TODENTJ. 2023;17(1). doi: 10.2174/18742106-v17-e230320-2022-74

[9] Sifakakis I, Eliades T. Adverse reactions to orthodontic materials. Aust Dent J. 2017;62 Suppl 1:20–8. doi: 10.1111/adj.12473 28297095

[10] Krishnan V. Orthodontic pain: from causes to management--a review. Eur J Orthod. 2007;29(2):170–9. doi: 10.1093/ejo/cjl081 17488999

[11] Antonio-Zancajo L, Montero J, Garcovich D, Alvarado-Lorenzo M, Albaladejo A, Alvarado-Lorenzo A. Comparative analysis of periodontal pain according to the type of precision orthodontic appliances: vestibular, lingual and aligners. a prospective clinical study. Biology (Basel). 2021;10(5):379. doi: 10.3390/biology10050379 33924818 PMC8145976

[12] Jaber ST, Al-Sabbagh R, Hajeer MY. Evaluation of the efficacy of laser-assisted flapless corticotomy in accelerating canine retraction: a split-mouth randomized controlled clinical trial. Oral Maxillofac Surg. 2022;26(1):81–9. doi: 10.1007/s10006-021-00963-x 33876339

[13] Gao M, Yan X, Zhao R, Shan Y, Chen Y, Jian F, et al. Comparison of pain perception, anxiety, and impacts on oral health-related quality of life between patients receiving clear aligners and fixed appliances during the initial stage of orthodontic treatment. Eur J Orthod. 2021;43(3):353–9. doi: 10.1093/ejo/cjaa037 32613250

[14] Withayanukonkij W, Chanmanee P, Promsawat M, Viteporn S, Leethanakul C. Root resorption during maxillary molar intrusion with clear aligners: a randomized controlled trial. Angle Orthod. 2023;93(6):629–37. doi: 10.2319/010723-14.1 37922387 PMC10633803

[15] Eissa O, Carlyle T, El-Bialy T. Evaluation of root length following treatment with clear aligners and two different fixed orthodontic appliances. A pilot study. J Orthod Sci. 2018;7:11. doi: 10.4103/jos.JOS_120_17 29963506 PMC6004740

[16] Huo M-L, Lei Y, Xin X, Zhang Y, Wang R-M. Effect of 2 kinds of appliances on anterior tooth root and alveolar bone in patients with mild to moderate overcrowding of ClassⅠmalocclusion. Shanghai Kou Qiang Yi Xue. 2024;33(4):407–10. 39478399

[17] Annamalaisamy S, Malthesh BS, Shashikumar GM, Shantharam S, Kumar PK. Comparative study of periodontal health in patients with fixed braces versus clear aligners. J Pharm Bioallied Sci. 2024;16(Suppl 4):S3790–2. doi: 10.4103/jpbs.jpbs_993_24 39927008 PMC11805022

[18] Albhaisi Z, Al-Khateeb SN, Abu Alhaija ES. Enamel demineralization during clear aligner orthodontic treatment compared with fixed appliance therapy, evaluated with quantitative light-induced fluorescence: a randomized clinical trial. Am J Orthod Dentofacial Orthop. 2020;157(5):594–601. doi: 10.1016/j.ajodo.2020.01.004 32354432

[19] Buschang PH, Chastain D, Keylor CL, Crosby D, Julien KC. Incidence of white spot lesions among patients treated with clear aligners and traditional braces. Angle Orthod. 2019;89(3):359–64. doi: 10.2319/073118-553.1 30556747 PMC8117696

[20] Çetin S, Akdeniz BS. Comparative study of proximal caries formation and decay, missing, filled teeth scores in clear aligners and fixed orthodontic treatments. Turk J Orthod. 2025;38(1):30–5. doi: 10.4274/TurkJOrthod.2024.2023.140 40150850 PMC11976324

[21] Azeem M, Ul Hamid W. Incidence of white spot lesions during orthodontic clear aligner therapy. Journal of the World Federation of Orthodontists. 2017;6(3):127–30. doi: 10.1016/j.ejwf.2017.07.001

[22] Yang T, Jiang L, Sun W, Zhu M, Jiang K, Li H, et al. The incidence and severity of open gingival embrasures in adults treated with clear aligners and fixed appliances: a retrospective cohort study. Head Face Med. 2023;19(1):30. doi: 10.1186/s13005-023-00375-0 37461116 PMC10351162

[23] Fraundorf EC, Araújo E, Ueno H, Schneider PP, Kim KB. Speech performance in adult patients undergoing Invisalign treatment. Angle Orthod. 2022;92(1):80–6. doi: 10.2319/122820-1037.1 34415296 PMC8691480

[24] Schaefer I, Braumann B. Halitosis, oral health and quality of life during treatment with Invisalign(®) and the effect of a low-dose chlorhexidine solution. J Orofac Orthop. 2010;71(6):430–41. doi: 10.1007/s00056-010-1040-6 21082306

[25] Ali Baeshen H, El-Bialy T, Alshehri A, Awadh W, Thomas J, Dhillon H, et al. The effect of clear aligners on speech: a systematic review. Eur J Orthod. 2023;45(1):11–9. doi: 10.1093/ejo/cjac018 35522548

[26] Yazdi M, Daryanavard H, Ashtiani AH, Moradinejad M, Rakhshan V. A systematic review of biocompatibility and safety of orthodontic clear aligners and transparent vacuum-formed thermoplastic retainers: Bisphenol-A release, adverse effects, cytotoxicity, and estrogenic effects. Dent Res J (Isfahan). 2023;20:41. doi: 10.4103/1735-3327.372658 37180685 PMC10166753

[27] Crego-Ruiz M, Jorba-García A. Assessment of the periodontal health status and gingival recession during orthodontic treatment with clear aligners and fixed appliances: a systematic review and meta-analysis. Med Oral Patol Oral Cir Bucal. 2023;28(4):e330–40. doi: 10.4317/medoral.25760 36641738 PMC10314350

[28] Ronchi Lemos C, Ventura Fadel MA, Polmann H, Meller Dias de Oliveira J, Pauletto P, Miron Stefani C, et al. Clear aligner’s adverse effects: a systematic review protocol. PLoS One. 2024;19(5):e0302049. doi: 10.1371/journal.pone.0302049 38696380 PMC11065214

[29] Page MJ, McKenzie JE, Bossuyt PM, Boutron I, Hoffmann TC, Mulrow CD, et al. The PRISMA 2020 statement: an updated guideline for reporting systematic reviews. BMJ. 2021;372:n71. doi: 10.1136/bmj.n71 33782057 PMC8005924

[30] Campbell M, McKenzie JE, Sowden A, Katikireddi SV, Brennan SE, Ellis S, et al. Synthesis without meta-analysis (SWiM) in systematic reviews: reporting guideline. BMJ. 2020;368:l6890. doi: 10.1136/bmj.l6890 31948937 PMC7190266

[31] Rayyan. AI-powered systematic review management platform. [cired 2025 Aug 5.]. https://www.rayyan.ai

[32] Ouzzani M, Hammady H, Fedorowicz Z, Elmagarmid A. Rayyan-a web and mobile app for systematic reviews. Syst Rev. 2016;5(1):210. doi: 10.1186/s13643-016-0384-4 27919275 PMC5139140

[33] Sider. ChatGPT sidebar with GPT-4o, Claude 3.5, Gemini 1.5 and AI tools [Internet]. [cited 2025 Jan 15]. https://sider.ai

[34] OpenAI. ChatGPT [Internet]. 2024 [cited 2025 Jul 7]. https://chat.openai.com

[35] Wan X, Wang W, Liu J, Tong T. Estimating the sample mean and standard deviation from the sample size, median, range and/or interquartile range. BMC Med Res Methodol. 2014;14:135. doi: 10.1186/1471-2288-14-135 25524443 PMC4383202

[36] Hozo SP, Djulbegovic B, Hozo I. Estimating the mean and variance from the median, range, and the size of a sample. BMC Med Res Methodol. 2005;5:13. doi: 10.1186/1471-2288-5-13 15840177 PMC1097734

[37] Automeris.io. Computer vision assisted data extraction from charts using WebPlotDigitizer. [cited 2025 Mar 5]. https://automeris.io

[38] Sterne JAC, Savović J, Page MJ, Elbers RG, Blencowe NS, Boutron I, et al. RoB 2: a revised tool for assessing risk of bias in randomised trials. BMJ. 2019;366:l4898. doi: 10.1136/bmj.l4898 31462531

[39] Sterne JA, Hernán MA, Reeves BC, Savović J, Berkman ND, Viswanathan M, et al. ROBINS-I: a tool for assessing risk of bias in non-randomised studies of interventions. BMJ. 2016;355:i4919. doi: 10.1136/bmj.i4919 27733354 PMC5062054

[40] McGuinness LA, Higgins JPT. Risk-of-bias VISualization (robvis): An R package and Shiny web app for visualizing risk-of-bias assessments. Res Synth Methods. 2021;12(1):55–61. doi: 10.1002/jrsm.1411 32336025

[41] Deeks JJ, Higgins JPT, Altman DG, McKenzie JE, Veroniki AA. Analysing data and undertaking meta-analyses. In: Higgins JPT, Thomas J, Chandler J, Cumpston M, Li T, Page MJ, et al., editors. Cochrane Handbook for Systematic Reviews of Interventions. Cochrane; 2024.

[42] Alfailany DT, Hajeer MY, Darwich K. The transparency of reporting “harms” encountered with the surgically assisted acceleration of orthodontic tooth movement in the published randomized controlled trials: a meta-epidemiological study. Prog Orthod. 2023;24(1):11. doi: 10.1186/s40510-023-00457-4 36941520 PMC10027979

[43] Stern C, Munn Z, Barker TH, Porritt K, Stone JC, Pap R, et al. Implementing GRADE in systematic reviews that adhere to JBI methodological conduct. JBI Evid Synth. 2024;22(3):351–8. doi: 10.11124/JBIES-23-00543 38385457

[44] Almagrami I, Almashraqi AA, Almaqrami BS, Mohamed AS, Wafaie K, Al-Balaa M, et al. A quantitative three-dimensional comparative study of alveolar bone changes and apical root resorption between clear aligners and fixed orthodontic appliances. Prog Orthod. 2023;24(1):6. doi: 10.1186/s40510-023-00458-3 36843193 PMC9968667

[45] Wang G, Yang L, Zhang Y-F, Luo S-L, Zheng J-W. A retrospective study on incisor root resorption in patients treated with bracketless invisible appliance and straight wire appliance. Shanghai Kou Qiang Yi Xue. 2017;26(1):121–4. 28474083

[46] Zhao R, Mei L, Long H, Jian F, Lai W. Changing clear aligners every 10 days or 14 days? A randomised controlled trial. Australas Orthod J. 2023;39(1):1–12. doi: 10.2478/aoj-2023-0002

[47] Diddige R, Negi G, Kiran KVS, Chitra P. Comparison of pain levels in patients treated with 3 different orthodontic appliances - a randomized trial. Med Pharm Rep. 2020;93(1):81–8. doi: 10.15386/mpr-1311 32133451 PMC7051823

[48] Fujiyama K, Honjo T, Suzuki M, Matsuoka S, Deguchi T. Analysis of pain level in cases treated with Invisalign aligner: comparison with fixed edgewise appliance therapy. Prog Orthod. 2014;15(1):64. doi: 10.1186/s40510-014-0064-7 25416143 PMC4240829

[49] Ali D, Abdal H, Alsaeed M. Comparison of self-rated pain and salivary alpha-amylase and cortisol levels during early stages of fixed orthodontic and clear aligner therapy. Acta Odontol Scand. 2023;81(8):627–32. doi: 10.1080/00016357.2023.2236214 37466389

[50] Almasoud NN. Pain perception among patients treated with passive self-ligating fixed appliances and Invisalign® aligners during the first week of orthodontic treatment. Korean J Orthod. 2018;48(5):326–32. doi: 10.4041/kjod.2018.48.5.326 30206531 PMC6123079

[51] Al-Dboush R, Rossi A, El-Bialy T. Impact of low intensity pulsed ultrasound on volumetric root resorption of maxillary incisors in patients treated with clear aligner therapy: A retrospective study. Dental Press J Orthod. 2023;28(2):e2321252. doi: 10.1590/2177-6709.28.2.e2321252.oar 37255132 PMC10229116

[52] White DW, Julien KC, Jacob H, Campbell PM, Buschang PH. Discomfort associated with Invisalign and traditional brackets: A randomized, prospective trial. Angle Orthod. 2017;87(6):801–8. doi: 10.2319/091416-687.1 28753032 PMC8317568

[53] Rucker JW. A prospective, longitudinal, study of initial discomfort associated with fixed and Invisalign treatment. St. Louis: Saint Louis University; 2012. https://www.proquest.com/dissertations-theses/global/docview/1534373

[54] Levrini L, Abbate GM, Migliori F, Orrù G, Sauro S, Caprioglio A. Assessment of the periodontal health status in patients undergoing orthodontic treatment with fixed or removable appliances: a microbiological and preliminary clinical study. Cumhur Dent J. 2013;16(4):296–307. doi: 10.7126/cdj.2013.1974

[55] Alcón S, Curto A, Alvarado M, Albaladejo A, Garcovich D, Alvarado-Lorenzo A. Comparative analysis of periodontal pain using two different orthodontic techniques, fixed multibrackets and removable aligners: a longitudinal clinical study with monthly follow-ups for 12 months. Applied Sciences. 2021;11(24):12013. doi: 10.3390/app112412013

[56] Khalil O, Abouelnour A, Abu-Shahba R. Apical root resorption accompanied orthodontic treatment using clear aligners versus fixed appliances: a CBCT comparative study. J Pharm Negat Results. 2023;14(Special Issue 2):750–60. doi: 10.47750/pnr.2023.14.S02.92

[57] Liu F, Wang Y, Luopei D, Qu X, Liu L. Comparison of fixed braces and clear braces for malocclusion treatment. BMC Oral Health. 2024;24(1):941. doi: 10.1186/s12903-024-04469-2 39143508 PMC11323350

[58] Pereira NC, Oltramari PVP, Conti PCR, Bonjardim LR, de Almeida-Pedrin RR, Fernandes TMF, et al. Frequency of awake bruxism behavior in orthodontic patients: randomized clinical trial. J Oral Rehabil. 2021;48(4):422–9. doi: 10.1111/joor.13130 33278836

[59] Mertoglu IE, Sahin D, Seker ED. Comparison of orthodontic adverse effects: braces versus clear aligners. J Clin Pediatr Dent. 2025;49(1):74–86. doi: 10.22514/jocpd.2025.007

[60] Alturki G, Jamel A, Alshuaybi A, Baeshen H, Farag AM. Perception of pain intensity and quality in patients treated with conventional fixed orthodontic appliances versus clear removable aligners: a pilot study. TODENTJ. 2024;18(1). doi: 10.2174/0118742106314583240801074709

[61] Damasceno Melo PE, Bocato JR, de Castro Ferreira Conti AC, Siqueira de Souza KR, Freire Fernandes TM, de Almeida MR, et al. Effects of orthodontic treatment with aligners and fixed appliances on speech. Angle Orthod. 2021;91(6):711–7. doi: 10.2319/110620-917.1 34037699 PMC8549558

[62] Mahayyudin MAM, Ibrar M, Jamil S, Islam ZUL, Malik F, Zaheer R. A comparative etudy of alveolar bone changes and apical root resorption between clear aligners and fixed orthodontic appliances. J Popul Ther Clin Pharmacol. 2024;31(8):1847–54. doi: 10.53555/x1kx7275

[63] Rossi A, Lagravère-Vich M, Heo G, Major PW, El-Bialy T. An evaluation of root resorption associated with the use of photobiomodulation during orthodontic treatment with clear aligners: a retrospective cohort pilot study. Angle Orthod. 2024;94(3):294–302. doi: 10.2319/081823-567.1 38412960 PMC11050451

[64] Alajmi S, Shaban A, Al-Azemi R. Comparison of short-term oral impacts experienced by patients treated with invisalign or conventional fixed orthodontic appliances. Med Princ Pract. 2020;29(4):382–8. doi: 10.1159/000505459 31842018 PMC7445657

[65] Allereau B, Sabouni W. Perception of pain in orthodontic treatment with thermoformed aligners. Orthod Fr. 2017;88(4):383–9. doi: 10.1051/orthodfr/2017028 29315072

[66] Peryer G, Golder S, Junqueira D, Vohra S, Loke YK. Adverse effects. In: Higgins JPT, Thomas J, Chandler J, Cumpston M, Li T, Page MJ, editors. Cochrane Handbook for Systematic Reviews of Interventions. Cochrane; 2024.

[67] Alvarado-Lorenzo A, Antonio-Zancajo L, Baptista H, Colino Gallardo P, Albaladejo-Martinez A, Garcovich D, et al. Comparative analysis of periodontal pain and quality of life in patients with fixed multibracket appliances and aligners (Invisalign®): longitudinal clinical study. BMC Oral Health. 2023;23(1):850. doi: 10.1186/s12903-023-03565-z 37951878 PMC10638788

[68] Costello CJ, Kerr B, Weir T, Freer E. The incidence and severity of root resorption following orthodontic treatment using clear aligners. Australas Orthod J. 2020;36(2):130–7.

[69] Gay G, Ravera S, Castroflorio T, Garino F, Rossini G, Parrini S, et al. Root resorption during orthodontic treatment with Invisalign®: a radiometric study. Prog Orthod. 2017;18(1):12. doi: 10.1186/s40510-017-0166-0 28503724 PMC5430001

[70] Kara-Boulad JM, Burhan AS, Hajeer MY, Nawaya FR, Jaber ST. CBCT-based assessment of apical root resorption and alveolar bone height following orthodontic treatment of Class I moderate crowding with labial vs. lingual fixed appliances in young adults: A randomized controlled trial. Int Orthod. 2025;23(2):100968. doi: 10.1016/j.ortho.2025.100968 39837069

[71] Vaquero Niño P, Perea Pérez B, Labajo González E, Santiago Sáez A, García Marín F. Reabsorción radicular durante el tratamiento ortodóncico: causas y recomendaciones de actuación. Científica Dent. 2011;8(1):61–70.

[72] Alam MK, Hajeer MY, Alruwaili RA, Alfaleh AA, Alhashash OB. Investigating the Relationship Between Orthodontic Treatment and Temporomandibular Joint Disorders in Adolescents. Journal of Pharmacy and Bioallied Sciences. 2024;16(Suppl 4):null. doi: 10.4103/jpbs.jpbs_1176_24 PMC11805211 39926840

[73] Uzunçıbuk H, Marrapodi MM, Meto A, Ronsivalle V, Cicciù M, Minervini G. Prevalence of temporomandibular disorders in clear aligner patients using orthodontic intermaxillary elastics assessed with diagnostic criteria for temporomandibular disorders (DC/TMD) axis II evaluation: A cross-sectional study. J Oral Rehabil. 2024;51(3):500–9. doi: 10.1111/joor.13614 38041596

[74] Saccomanno S, Laganà D, Mastrapasqua R, Giancaspro S, Manenti RJ, Saran S. The relationship between TMJ symptoms and orthodontic treatments: a survey on 236 orthodontic patients. J Biol Regul Homeost Agents. 2021;35(3 Suppl. 1):197–204. doi: 10.23812/21-3supp1-22 34289679

[75] Zhang Y, Wang X, Wang J, Gao J, Liu X, Jin Z, et al. IPR treatment and attachments design in clear aligner therapy and risk of open gingival embrasures in adults. Prog Orthod. 2023;24(1):1. doi: 10.1186/s40510-022-00452-1 36617584 PMC9826765

[76] Handelman CS. The anterior alveolus: its importance in limiting orthodontic treatment and its influence on the occurrence of iatrogenic sequelae. Angle Orthod. 1996;66(2):95–109; discussion 109-10. doi: 10.1043/0003-3219(1996)066<0095:TAAIII>2.3.CO;2 8712499

[77] Garib DG, Yatabe MS, Ozawa TO, Silva Filho DOG. Alveolar bone morphology under the perspective of the computed tomography: defining the biological limits of tooth movement. Dent Press J Orthod. 2010;15(5):192–205. doi: 10.1590/S2176-94512010000500023

[78] Evangelista K, Vasconcelos K de F, Bumann A, Hirsch E, Nitka M, Silva MAG. Dehiscence and fenestration in patients with Class I and Class II Division 1 malocclusion assessed with cone-beam computed tomography. Am J Orthod Dentofacial Orthop. 2010;138(2):133.e1-7; discussion 133-5. doi: 10.1016/j.ajodo.2010.02.021 20691344

[79] Allahham DO, Kotsailidi EA, Barmak AB, Rossouw PE, El-Bialy T, Michelogiannakis D. Association between nonextraction clear aligner therapy and alveolar bone dehiscences and fenestrations in adults with mild-to-moderate crowding. Am J Orthod Dentofacial Orthop. 2023;163(1):22-32.e4. doi: 10.1016/j.ajodo.2021.08.022 36153200

[80] Angelopoulos GG, Kanarelis P, Vagdouti G, Zavlanou A, Sifakakis I. Applied Sciences. 2021;11(21):10074. doi: 10.3390/app112110074

[81] Pogal-Sussman-Gandia CB, Tabbaa S. Effects of Invisalign treatment on speech articulation. Orthod Craniofac Res. 2019;22(3):195–200. doi: 10.1111/ocr.12305 31326368

[82] Low B, Lee W, Seneviratne CJ, Samaranayake LP, Hägg U. Ultrastructure and morphology of biofilms on thermoplastic orthodontic appliances in “fast” and “slow” plaque formers. Eur J Orthod. 2011;33(5):577–83. doi: 10.1093/ejo/cjq126 21187528

[83] Rouzi M, Jiang Q, Zhang H, Li X, Long H, Lai W. Characteristics of oral microbiota and oral health in the patients treated with clear aligners: a prospective study. Clin Oral Investig. 2023;27(11):6725–34. doi: 10.1007/s00784-023-05281-y 37775585

[84] Shpack N, Greenstein RB-N, Gazit D, Sarig R, Vardimon AD. Efficacy of three hygienic protocols in reducing biofilm adherence to removable thermoplastic appliance. Angle Orthod. 2014;84(1):161–70. doi: 10.2319/012413-75.1 23786595 PMC8683063

[85] Sifakakis I, Papaioannou W, Papadimitriou A, Kloukos D, Papageorgiou SN, Eliades T. Salivary levels of cariogenic bacterial species during orthodontic treatment with thermoplastic aligners or fixed appliances: a prospective cohort study. Prog Orthod. 2018;19(1):25. doi: 10.1186/s40510-018-0230-4 30066184 PMC6068060

