# Supplementary material for: Adverse effects of removable orthodontic aligners: A systematic review with single-arm meta-analysis
Source: PLoS One. 2026 Jul 20;21(7):e0350741. doi: 10.1371/journal.pone.0350741 (PMC13384317; doi:10.1371/journal.pone.0350741)
Supplement: S1 Checklist — (DOCX) [file pone.0350741.s001.docx]

# Supplementary Material 1

# PRISMA 2020 Checklist – Orthodontic Aligners and Their Adverse Effects: a Comprehensive Systematic Review and Meta-Analysis

This checklist follows the PRISMA 2020 statement (Page MJ et al., BMJ 2021;372:n71) and corresponds to the final version of the manuscript entitled 'Orthodontic Aligners and Their Adverse Effects: a Comprehensive Systematic Review and Meta-Analysis'. All page numbers have been updated to match the final manuscript.

| **Section and Topic** | **Item #** | **Checklist item** | **Location where item is reported** |
| --- | --- | --- | --- |
| TITLE | 1 | Identify the report as a systematic review. | Page 1 – Title |
| ABSTRACT | 2 | See the PRISMA 2020 for Abstracts checklist. | Pages 1–2 – Abstract |
| INTRODUCTION | 3 | Describe the rationale for the review in the context of existing knowledge. | Pages 3–6 – Introduction |
|  | 4 | Provide an explicit statement of the objective(s) or question(s) the review addresses. | Page 6 – Final Introcution |
| METHODS | 5 | Specify the inclusion and exclusion criteria and how studies were grouped for the syntheses. | Pages 6-8 – Methods: Eligibility Criteria |
|  | 6 | Specify all information sources and the date of last search. | Page 8 – Information Sources |
|  | 7 | Present the full search strategies for all databases, including filters and limits used. | Pages 8-9 - Supporting information (S3 Table) |
|  | 8 | Specify the methods used to decide whether a study met inclusion criteria, including details of automation tools. | Pages 9–11 – Study Selection Process |
|  | 9 | Specify data collection methods and automation tools used. | Page 11-12 – Data items and collection |
|  | 10a | List and define all outcomes for which data were sought. | Pages 12-14, Tables 1–2 |
|  | 10b | List and define all other variables collected (e.g., participant and intervention characteristics). | Page 12 – Tables 1–2 |
|  | 11 | Specify the methods used to assess risk of bias in included studies. | Pages 13-15 – Risk of bias/quality assessment |
|  | 12 | Specify for each outcome the effect measures used. | Page 15-16 – Synthesis Methods |
|  | 13a | Describe the process used to decide which studies were eligible for each synthesis. | Page 15 – Synthesis methods |
|  | 13b | Describe data preparation and conversions performed. | Page 15 – Synthesis methods |
|  | 13c | Describe methods used to tabulate or display individual study results. | Pages 20-40, Figures 2–4 |
|  | 13d | Describe synthesis methods and models, heterogeneity measures, and software used. | Pages 15–17 – Statistical synthesis |
|  | 13e | Describe any methods used to explore causes of heterogeneity. | Page 16–17 – Synthesis methods |
|  | 14 | Describe methods used to assess reporting bias. | Page 17 – Reporting Bias Assessment |
|  | 15 | Describe methods used to assess certainty in the evidence (GRADE). | Page 17-18 – Certainty Assessment (GRADE) |
| RESULTS | 16a | Describe the search and selection results with a flow diagram. | Page 18-19 – Figure 2 |
|  | 16b | Cite excluded studies and reasons for exclusion. | Pages 60–65, Supporting information – Excluded Studies (S7) |
|  | 17 | Cite each included study and present its characteristics. | Pages 20–45 (Table 1 A–C) |
|  | 18 | Present risk of bias assessments for each study. | Pages 45–48 – Figure 3 |
|  | 19 | Present results of individual studies (summary statistics and effect estimates). | Pages 48–55, Tables 2–3 |
|  | 20a | Summarize characteristics and risk of bias among contributing studies for each synthesis. | Pages 48–58 – Quantitative Synthesis |
|  | 20b | Present results of all meta-analyses and measures of heterogeneity. | Pages 50–58 – Figures 5–7 |
|  | 20c | Present results of investigations of possible causes of heterogeneity | Pages 55–58 |
|  | 21 | Present assessments of risk of bias due to missing results (reporting bias) | Pages 58–59 – Reporting bias assessment |
|  | 22 | Present assessments of certainty (GRADE). | Pages 59–60 |
| DISCUSSION | 23a | Interpret results in context of other evidence. | Pages 60–66 – Discussion |
|  | 23b | Discuss limitations of the included evidence. | Pages 65–66 – Discussion |
|  | 23c | Discuss limitations of the review process. | Page 66 – Discussion |
|  | 23d | Discuss implications for practice, policy, and future research. | Pages 66–67 – Discussion |
| OTHER INFORMATION | 24a | Provide registration information for the review. | Page 6 – PROSPERO CRD42023458491 |
|  | 24b | Indicate where the protocol can be accessed. | Page 6 – Published in PLOS ONE 2024; Reference [15] |
|  | 24c | Describe and explain any protocol amendments. | No deviations from the registered protocol were made. |
|  | 25 | Describe sources of financial or non-financial support. | Page 68 – Funding |
|  | 26 | Declare any competing interests of the authors. | Page 68 – Competing Interests |
|  | 27 | Report availability of data, code, and other materials. | Page 18, 68– Data Availability |
