# Supplementary material for: Adverse effects of removable orthodontic aligners: A systematic review with single-arm meta-analysis
Source: PLoS One. 2026 Jul 20;21(7):e0350741. doi: 10.1371/journal.pone.0350741 (PMC13384317; doi:10.1371/journal.pone.0350741)
Supplement: S2 Checklist — (DOCX) [file pone.0350741.s002.docx]

# Supplementary Material 2

# SWiM 2020 Checklist – Orthodontic Aligners and Their Adverse Effects: a Comprehensive Systematic Review and Meta-Analysis

This checklist follows the SWiM (Synthesis Without Meta-analysis) reporting guideline (Campbell et al., BMJ 2020;368:l6890) and corresponds to the final version of the manuscript entitled 'Orthodontic Aligners and Their Adverse Effects: a Comprehensive Systematic Review and Meta-Analysis'. All page numbers have been updated to match the final manuscript.

| SWiM reporting item | Item description | Page in manuscript where item is reported | Other* |
| --- | --- | --- | --- |
| 1a. Grouping studies for synthesis | Provide a description of, and rationale for, the groups used in the synthesis (e.g., groupings of populations, interventions, outcomes, study design). | Page 15 – Synthesis methods: studies grouped by outcome domain, study design (RCTs, non-RCTs, cohort studies), measurement methods, and follow-up duration. | Defined in 'Synthesis methods' section. |
| 1b. Changes from protocol | Detail and provide rationale for any changes made subsequent to the protocol in the groups used in the synthesis. | Page 15 – No major deviations; grouping followed pre-specified protocol. | No amendments from protocol (see PROSPERO CRD42023458491). |
| 2. Standardized metric and transformations | Describe the standardized metric for each outcome and any methods used to transform data, citing methodological guidance. | Pages 15–16 – Means and SDs standardized from medians/ranges using Wan et al. (2014) and Hozo et al. (2005). | Transformation equations cited in references [22–23]. |
| 3. Synthesis methods | Describe and justify the methods used to synthesize effects when meta-analysis was not possible. | Pages 16–17 – Narrative synthesis using SWiM approach, structured by outcome domain and follow-up duration. | See 'Narrative Synthesis' section. |
| 4. Criteria for prioritizing results | Provide criteria used to select studies or prioritize results for synthesis or conclusions. | Pages 15–17 – Studies were prioritized for synthesis when outcomes were conceptually comparable and reported with sufficient methodological consistency across studies. | Described in synthesis section and Table 2. |
| 5. Investigation of heterogeneity | State methods used to examine heterogeneity in reported effects when meta-analysis was not feasible. | Pages 16–17 – Heterogeneity explored qualitatively based on differences in study design, outcome definitions, measurement instruments, and follow-up duration; quantitative heterogeneity assessed using I² and τ² in meta-analyses. | Quantitative heterogeneity (I², τ²) reported for meta-analyses only. |
| 6. Certainty of evidence | Describe methods used to assess certainty of synthesis findings. | Pages 59–60 – Certainty assessed via GRADE across five domains; summarized in Table 4. | GRADE summary in 'Certainty of Evidence' section. |
| 7. Data presentation methods | Describe graphical and tabular methods used to present results and key study characteristics. | Pages 20–58 – Tables 1–3 summarize findings; Figures 2–7 present results (flowchart, risk of bias, forest plots). | Structured by study design and outcome. |
| 8. Reporting results | For each comparison and outcome, describe synthesized findings and indicate contributing studies. | Pages 48–58 – Results synthesized narratively with references to contributing studies for each outcome. | Tables 2–3 provide summary of key results and clinical implications. |
| 9. Limitations of the synthesis | Report limitations of the synthesis methods or groupings and how these affect conclusions. | Pages 65–66 – Discussion: limitations of heterogeneity, outcome variability, and generalizability discussed. | Linked to SWiM reporting; see 'Limitations' section. |

PRISMA = Preferred Reporting Items for Systematic Reviews and Meta-Analyses.
*If the information is not provided in the systematic review, indicate where it is available (e.g., protocol, supplementary material, or published article).
