# Supplementary material for: Adverse effects of removable orthodontic aligners: A systematic review with single-arm meta-analysis
Source: PLoS One. 2026 Jul 20;21(7):e0350741. doi: 10.1371/journal.pone.0350741 (PMC13384317; doi:10.1371/journal.pone.0350741)
Supplement: S3 Table — (DOCX) [file pone.0350741.s003.docx]

**Supplementary Material 3**

**Search Strategy**

| **Database** | **Search Strategy** | **Results** |
| --- | --- | --- |
| MEDLINE (via PubMed) | ("Invisalign"[Title/Abstract] OR "Invisible Appliance"[Title/Abstract] OR "Invisible Appliances"[Title/Abstract] OR "Aligners"[Title/Abstract] OR "Aligner"[Title/Abstract]) AND Orthodont*[Title/Abstract] | 1673 |
| Cochrane (Central) | (Invisalign OR "Invisible Appliance" OR "Invisible Appliances" OR Aligners OR Aligner):ti,ab,kw AND Orthodont*:ti,ab,kw | 787 |
| Embase | (Invisalign:ab,ti OR "Invisible Appliance":ab,ti OR "Invisible Appliances":ab,ti OR Aligners:ab,ti OR Aligner:ab,ti) AND Orthodont*:ab,ti | 1551 |
| Scopus | TITLE-ABS("Invisalign" OR "Invisible Appliance" OR "Invisible Appliances" OR "Aligners" OR "Aligner") AND TITLE-ABS(Orthodont*) | 2231 |
| Web of Science | TS=("Invisalign" OR "Invisible Appliance" OR "Invisible Appliances" OR "Aligners" OR "Aligner") AND TS=(Orthodont*) | 1572 |
| Livivo | (Invisalign OR "Invisible Appliance" OR "Invisible Appliances" OR Aligners OR Aligner) AND Orthodont* | 321 |
| LILACS (via BVS) | (Invisalign OR "Invisible Appliance" OR "Invisible Appliances" OR Aligners OR Aligner) AND (Orthodont*) | 979 |
| ProQuest | TI,AB(Invisalign OR "Invisible Appliance" OR "Invisible Appliances" OR Aligners OR Aligner) AND TI,AB(Orthodont*) | 250 |
| Google Scholar | "Invisalign" OR "Invisible Appliance" OR "Invisible Appliances" OR "Aligners" OR "Aligner" AND Orthodont* | 100 |
