# Supplementary material for: Adverse effects of removable orthodontic aligners: A systematic review with single-arm meta-analysis
Source: PLoS One. 2026 Jul 20;21(7):e0350741. doi: 10.1371/journal.pone.0350741 (PMC13384317; doi:10.1371/journal.pone.0350741)
Supplement: S4 — (DOCX) [file pone.0350741.s004.docx]

**Supplementary Material 4**

**Risk of Bias Prompt (RoB 2) for Information Retrieval in the Studies**

Act as a researcher and specialist in systematic reviews. Assume the stance of an experienced methodologist in risk of bias assessment.

Complete the RoB 2 tool for each randomized clinical trial provided.
 Use **only** the studies supplied; do not consult any external databases or prior knowledge. All evidence must be extracted from the attached manuscript(s).

For each study and for each domain of RoB 2:

1. **Extract the relevant information.**
2. **Identify in the study text the passages that answer the signaling questions regarding:**
   - **Selection of participants**
   - **Execution of the interventions**
   - **Deviations from the intended intervention**
   - **Measurement of outcomes**
   - **Selective reporting of results**
3. **Clearly state the outcome being assessed** (e.g., pain at 24 hours, root resorption, etc.).
4. **Specify whether the analysis was conducted as intention-to-treat or per-protocol**, since this affects interpretation of deviations and the analysis. Adapt the signaling questions and judgments accordingly based on that definition.
5. **Justify the judgment.**
   - Explain why the domain was rated as “low risk,” “some concerns,” or “high risk of bias,” explicitly comparing the RoB 2 criteria with the extracted content.
   - Ground your explanations in the methodological standards of the RoB 2 tool.
6. **Indicate the source of each piece of information used in the justification** by including, in parentheses:
   - The section (e.g., “Methods”),
   - The line number, and
   - The page of the manuscript where the evidence was found.
7. *Example citation format:* (“Methods,” line 3, page 5)

*Deliver the filled RoB 2 assessments with these detailed justifications and properly referenced source locations for each domain and outcome.*

*_________________________________________________________________________*

***Risk of Bias Prompt (ROBINS-I) for Information Retrieval in the Studies***

*Act as a researcher and specialist in systematic reviews. Assume the stance of an experienced methodologist in risk of bias assessment.*

*Complete the* ***ROBINS-I*** *tool for each non-randomized study of interventions provided. Use* ***only*** *the supplied studies; do not consult external databases or prior knowledge. All evidence must be extracted from the attached manuscript(s).*

*For each study and for each domain of ROBINS-I (pre-intervention, at-intervention, and post-intervention stages):*

1. ***Define the target trial and effect of interest.***
   - *Clearly state what hypothetical ideal (randomized) trial the study is being compared to and which effect is being assessed (e.g., effect of the intervention as planned, the intended effect, or the effect under adherence).*
   - *Specify the outcome under evaluation (e.g., pain at 24 hours, root resorption, periodontal inflammation) and the exact contrast (intervention vs comparator).*
2. ***Extract the relevant information.***
3. ***Identify in the study text the passages that answer the signaling questions for each ROBINS-I domain:***
   - ***Bias due to confounding*** *(pre-intervention): Were important confounders measured and appropriately accounted for? Is there evidence of baseline imbalance on prognostic factors?*
   - ***Bias in selection of participants into the study*** *(pre-intervention): Were inclusion/exclusion processes related to both intervention and outcome?*
   - ***Bias in classification of interventions*** *(at intervention): Was the intervention status correctly classified? Were there misclassifications?*
   - ***Bias due to deviations from intended interventions*** *(at/post intervention): Were there deviations from the intended intervention, and were they related to the intervention group and outcome? Were co-interventions handled appropriately?*
   - ***Bias due to missing data*** *(post-intervention): Is there loss to follow-up or missing outcome data that could depend on both intervention and outcome?*
   - ***Bias in measurement of outcomes*** *(post-intervention): Were outcome assessors blinded or could measurement differ by intervention group?*
   - ***Bias in selection of the reported result*** *(post-intervention): Is there evidence of selective reporting or multiple analyses with cherry-picking?*
4. ***Specify whether and how the study adjusted for confounding and other relevant factors.***
   - *Describe the methods used (e.g., regression adjustment, propensity scores, stratification), and whether time-varying confounding or post-exposure adjustment could introduce bias.*
   - *State if the analysis aligns with the effect of interest defined in step 1.*
5. ***Justify each judgment.***
   - *For every domain, explain why it was rated as “low risk,” “some concerns,” “serious risk,” or “critical risk” of bias, explicitly comparing the ROBINS-I criteria with the extracted content.*
   - *Discuss any assumptions or deviations from the ideal target trial that influenced the rating.*
6. ***Indicate the source of each piece of information used in the justification*** *by including, in parentheses:*
   - *The section (e.g., “Methods”),*
   - *The line number, and*
   - *The page of the manuscript where the evidence was found.*
7. *Example citation format: (“Methods,” line 12, page 7)*

*Deliver the completed ROBINS-I assessments for each study, outcome, and effect of interest, with detailed justifications and precise source locations for all judgments.*
