# Supplementary material for: Adverse effects of removable orthodontic aligners: A systematic review with single-arm meta-analysis
Source: PLoS One. 2026 Jul 20;21(7):e0350741. doi: 10.1371/journal.pone.0350741 (PMC13384317; doi:10.1371/journal.pone.0350741)
Supplement: S5 — (DOCX) [file pone.0350741.s005.docx]

# S5 File – Outcome harmonization procedures and worked examples

Outcome harmonization was performed to enable quantitative synthesis across studies with heterogeneous reporting formats, measurement instruments, and units of analysis. Procedures followed methodological guidance for adverse effects synthesis, prioritizing clinical interpretability and transparency.

**Stepwise harmonization approach**

1. **Standardization of measurement scales**

When outcomes were reported using different scales, values were converted to a common metric.

Visual Analogue Scale (VAS):

0–100 mm → converted to 0–10 by dividing by 10

0–10 numeric scales → used directly

10-point categorical scales → assumed linear equivalence

Example:

A mean pain score of 45 mm on a 0–100 mm VAS was converted to 4.5 on a 0–10 scale.

1. **Harmonization of summary statistics**

When studies reported medians and ranges instead of means and standard deviations (SDs):

Means and SDs were estimated using established methods [22,23]

Example:

Median pain = 3 (range 1–6) → converted to mean and SD using Wan et al. (2014)

1. **Alignment of units of analysis**

When outcomes were reported at the tooth or site level:

Data were aggregated to the study (patient) level

Example:

Root resorption measured in multiple teeth per patient was averaged to obtain a single value per participant or study arm before pooling.

1. **Derivation of change scores**

When only baseline and follow-up means were available:

Change = follow-up − baseline

SD of change was estimated using assumed correlations (r = 0.3–0.7)

Example:

Baseline = 2.0, Follow-up = 1.0 → Change = −1.0

SD_change estimated assuming r = 0.3–0.7

Sensitivity analyses were conducted to assess the impact of these assumptions.

1. **Selection of outcome format**

Post-intervention means were prioritized

Change scores were used only when necessary

Different formats were not pooled together

Rationale (aligned with Cochrane guidance)
