# Supplementary material for: Adverse effects of removable orthodontic aligners: A systematic review with single-arm meta-analysis
Source: PLoS One. 2026 Jul 20;21(7):e0350741. doi: 10.1371/journal.pone.0350741 (PMC13384317; doi:10.1371/journal.pone.0350741)
Supplement: S6 — (DOCX) [file pone.0350741.s006.docx]

## **S6 – Assessment of selective reporting and completeness of adverse effects**

| **Study** | **Harms mentioned in Methods** | **Harms reported** | **Missing harms (not assessed/reported)** | **Judgment (Cochrane-aligned)** |
| --- | --- | --- | --- | --- |
| Zhao 2023 | Pain | Pain (complete) | Periodontal, root resorption, enamel | High risk |
| Gao 2020 | Pain, anxiety, QoL | Same (complete) | Biological harms (periodontal, enamel, root) | High risk |
| Khalil 2023 | Root resorption | Root resorption (complete) | Pain, periodontal, enamel | Some concerns |
| Pereira 2020 | Bruxism, pain | Same (complete) | Biological harms | High risk |
| Rucker 2012 | Discomfort | Same (complete) | All clinical harms | High risk |
| Schaefer 2010 | Periodontal, halitosis | Same (complete) | Other domains limited | Low risk |
| White 2017 | Pain | Same (complete) | Biological harms | High risk |
| Withayanukonkij 2023 | Root resorption | Same (complete) | Other harms | Some concerns |
| Yang 2023 | Gingival embrasures | Same (complete) | Other harms | Some concerns |
| Ali 2023 | Pain, biomarkers | Same (complete) | Clinical harms | High risk |
| Almagrami 2023 | Bone, root | Same (complete) | Other harms | Some concerns |
| Antonio-Zancajo 2021 | Pain | Same (complete) | Other harms | High risk |
| Buschang 2019 | WSL | Same (complete) | Other harms | Some concerns |
| Damasceno-Melo 2021 | Speech | Same (complete) | Biological harms | High risk |
| Diddige 2019 | Pain | Same (complete) | Other harms | High risk |
| Eissa 2018 | Root resorption | Same (complete) | Other harms | Some concerns |
| Fraundorf 2022 | Speech | Same (complete) | Biological harms | High risk |
| Fujiyama 2014 | Pain | Same (complete) | Other harms | High risk |
| Levrini 2013 | Periodontal, microbiology | Same (complete) | Limited scope | Low risk |
| Albhaisi 2020 | WSL, plaque | Same (complete) | Other harms | Some concerns |
| Alcón 2021 | Pain | Same (complete) | Other harms | High risk |
| Al-Dboush 2023 | Root resorption | Same (complete) | Other harms | Some concerns |
| Almasoud 2018 | Pain | Same (complete) | Other harms | High risk |
| Annamalaisamy 2024 | Periodontal | Same (complete) | Other harms | Some concerns |
| Çetin 2025 | Caries, DMFT | Same (complete) | Other harms | Some concerns |
| Mahayyudin 2024 | Bone, root | Same (complete) | Other harms | Some concerns |
| Mertoglu 2025 | Multidomain harms | Multidomain (complete) | Minimal | Low risk |
| Rossi 2024 | Root resorption | Same (complete) | Other harms | Some concerns |
| Zhang 2025 | Gingivitis | Same (complete) | Other harms | Some concerns |
| Alam 2024 | Discomfort (implicit) | Discomfort | All other harms | High risk |
| Alturki 2024 | Pain | Same (complete) | Other harms | High risk |
| Liu & Song 2024 | WSL | Same (complete) | Other harms | Some concerns |
