# Supplementary material for: Adverse effects of removable orthodontic aligners: A systematic review with single-arm meta-analysis
Source: PLoS One. 2026 Jul 20;21(7):e0350741. doi: 10.1371/journal.pone.0350741 (PMC13384317; doi:10.1371/journal.pone.0350741)
Supplement: S7 — (DOCX) [file pone.0350741.s007.docx]

**Supplementary Material 5**

**Excluded articles and reasons for exclusion.**

| **Author** | **Reason for exclusion** |
| --- | --- |
| Abbate et al. (2015)[1] | 2 |
| Abdullah et al. (2020)[2] | 2 |
| Abu Alhaija et al. (2019)[3] | 2 |
| Akram et al. (2022)[4] | 2 |
| Al-Dboush et al. (2023)[5] | 3 |
| Al-Moghrabi et al. (2018)[6] | 2 |
| Al-Tayar et al. (2023)[7] | 2 |
| Al-Worafi et al. (2024)[8] | 1 |
| Alajmi et al. (2020)[9] | 4 |
| Alanazi et al. (2022)[10] | 2 |
| Alasiri et al. (2024)[11] | 3 |
| Alfawal et al. (2022)[12] | 2 |
| Allahham et al. (2023)[13] | 4 |
| Allareddy et al. (2017)[14] | 4 |
| Allereau and Sabouni (2017)[15] | 4 |
| AlSeraidi et al. (2021)[16] | 2 |
| Alvarado-Lorenzo et al. (2023)[17] | 2 |
| Aman et al. (2018)[18] | 4 |
| Ana-Madalina et al. (2019)[19] | 2 |
| Angelopoulos et al. (2021)[20] | 2 |
| Antonio-Zancajo et al. (2020)[21] | 2 |
| Ashutosh et al. (2023)[22] | 2 |
| Azaripour et al. (2015)[23] | 2 |
| Azeem and UI Hamid (2017)[24] | 4 |
| Ba-Hattab et al. (2025)[25] | 1 |
| Bai et al. (2009)[26] | 2 |
| Balboni et al. (2023)[27] | 4 |
| Bargellini et al. (2022)[28] | 1 |
| Barreda et al. (2020)[29] | 2 |
| Barreda et al. (2020)[30] | 2 |
| Baseer et al. (2021)[31] | 2 |
| Belgal et al. (2023)[32] | 4 |
| Boggio and Cozzani (2024)[33] | 4 |
| Bowman et al. (2023)[34] | 2 |
| Bräscher et al. (2016)[35] | 2 |
| Brignardello-Petersen (2019)[36] | 3 |
| Caldas et al. (2024)[37] | 3 |
| Caruso et al. (2019)[38] | 2 |
| Caruso et al. (2021)[39] | 2 |
| Casteluci et al. (2021)[40] | 1 |
| Castroflorio et al. (2018)[41] | 1 |
| Chan et al. (2024)[42] | 1 |
| Chauhan et al. (2024)[43] | 2 |
| Chen et al. (2022)[44] | 2 |
| Chen et al. (2022)[45] | 2 |
| Ciavarella et al. (2022)[46] | 2 |
| Clements et al. (2003)[47] | 2 |
| Cohen-Lévy et al. (2022)[48] | 2 |
| Costello et al. (2020)[49] | 4 |
| Dianiskova et al. (2023)[50] | 4 |
| Donovan and Millett (2024)[51] | 2 |
| Ertugrul and Veli (2022)[52] | 4 |
| Falconi et al. (2020)[53] | 4 |
| Favero et al. (2023)[54] | 3 |
| Feng et al. (2024)[55] | 3 |
| Flores-Mir et al. (2018)[56] | 4 |
| Fountoulaki and Thurzo (2022)[57] | 2 |
| Fowler and Sameshima (2010)[58] | 2 |
| Frenkel and Bollen (2022)[59] | 2 |
| Gandia and Tabbaa (2015)[60] | 4 |
| Garret and Buschang (2012)[61] | 4 |
| Gay et al. (2017)[62] | 4 |
| Gowda et al. (2021)[63] | 2 |
| Gu et al. (2022)[64] | 4 |
| Gupta et al. (2023)[65] | 4 |
| Hakami (2023)[66] | 4 |
| Han (2015)[67] | 2 |
| Han et al. (2020)[68] | 2 |
| Harriott and Myneni Venkatasatya (2017)[69] | 2 |
| He et al. (2025)[70] | 2 |
| Heleiwa-Ferioli and Cruz-Vigo (2024)[71] | 2 |
| Hellak et al. (2016)[72] | 2 |
| Huang et al. (2025)[73] | 1 |
| Husain et al. (2024)[74] | 1 |
| Iglesias-Linares (2017)[75] | 2 |
| Jaber et al. (2022)[76] | 2 |
| Jan and Lin, G (2023)[77] | 2 |
| Jung (2021)[78] | 4 |
| Jyotirmay et al. (2021)[79] | 2 |
| Karkhanechi et al. (2013)[80] | 2 |
| Kim et al. (2024)[81] | 2 |
| Klein and Araújo (2013)[82] | 2 |
| Knode et al. (2025)[83] | 2 |
| Kobylyanskyy et al. (2024)[84] | 3 |
| Krieger et al. (2013)[85] | 4 |
| Kuncio et al. (2007)[86] | 2 |
| Kuroda et al. (2014)[87] | 2 |
| Kurnaz and Buyukcavus (2024)[88] | 1 |
| Kurtzner and Al-Jewair (2019)[89] | 2 |
| Levrini et al. (2015)[90] | 2 |
| Levrini et al. (2023)[91] | 4 |
| Lin et al. (2022)[92] | 4 |
| Liu et al. (2025)[93] | 3 |
| Liu et al. (2024)[94] | 3 |
| Liu et al. (2021)[95] | 4 |
| Lou et al. (2021)[96] | 4 |
| Lz et al. (2022)[97] | 2 |
| Madariaga et al. (2020)[98] | 2 |
| Marcelino et al. (2023)[99] | 2 |
| McKenna (2001)[100] | 3 |
| Miguel et al. (2024)[101] | 3 |
| Miller et al. (2007)[102] | 2 |
| Moon and Allareddy (2023)[103] | 2 |
| Mulla Issa et al. (2020)[104] | 2 |
| Nedwed and Miethke (2005)[105] | 4 |
| Nicholson (2011)[106] | 2 |
| Nota et al. (2021)[107] | 4 |
| Ozturk et al. (2025)[108] | 3 |
| Pacheco-Pereira et al. (2018)[109] | 2 |
| Paes-Souza et al. (2023)[110] | 4 |
| Parrini et al. (2018)[111] | 2 |
| Patterson and Kim (2018)[112] | 2 |
| Pescheret and Kusnoto (2017)[113] | 2 |
| Pittar et al. (2024)[114] | 4 |
| Pittar et al. (2023)[115] | 2 |
| Pogal-Sussman-Gandia et al. (2019)[116] | 4 |
| Risinger et al. (2023)[117] | 2 |
| Rouzi et al. (2023)[118] | 2 |
| Rouzi et al. (2023)[119] | 4 |
| Sabban et al. (2024)[120] | 1 |
| Saccomanno et al. (2021)[121] | 4 |
| Saccomanno et al. (2022)[122] | 4 |
| Sánchez Gómez (1996)[123] | 3 |
| Santonocito and Polizzi (2022)[124] | 3 |
| Sauer et al. (2023)[125] | 2 |
| Sharma et al. (2021)[126] | 2 |
| Shokeen et al. (2022)[127] | 2 |
| Steele et al. (2022)[128] | 2 |
| Tepedino et al. (2023)[129] | 2 |
| Thavarajah and Thennukonda (2015)[130] | 4 |
| Toyokama-Sperandio et al. (2021)[131] | 2 |
| Tran et al. (2020)[132] | 4 |
| Tunca (2024)[133] | 2 |
| Wadia (2023)[134] | 4 |
| Wang et al. (2023)[135] | 4 |
| Xu et al. (2022)[136] | 2 |
| Xu et al. (2022)[137] | 2 |
| Yi et al. (2018)[138] | 4 |
| Zamora-Martínez et al. (2021)[139] | 4 |
| Zhang et al. (2023)[140] | 4 |
| Zhao et al. (2020)[141] | 2 |
| Zheng et al. (2023)[142] | 2 |
| Zybutz et al. (2021)[143] | 2 |

| **Legend:** | |
| --- | --- |
| **1** | Wrong population |
| **2** | Wrong intervention |
| **3** | Wrong outcome |
| **4** | Wrong study design |

**REFERENCES**

1. Abbate GM, Caria MP, Montanari P *et al.* Periodontal health in teenagers treated with removable aligners and fixed orthodontic appliances. *Fortschritte Kieferorthopadie J Orofac Orthop* 2015;**76**:240â250.

2. Abdullah AAN, Ibrahim A, Serene AB *et al.* Relationship between oral health impacts and personality profiles among orthodontic patients treated with Invisalign clear aligners. *Sci Rep Vol 10 Iss 1 Pp 1-* 2020;**12**.

3. Abu Alhaija ESJ, Al-Abdallah SY, Taha NA. A comparative study of initial changes in pulpal blood flow between clear aligners and fixed orthodontic appliances. *Am J Orthod Dentofac Orthop* 2019;**156**:603–10.

4. Akram S, Zeeshan M, Masood M *et al.* The Impact of Orthodontic fixed appliance and clear plastic aligner in periodontal health; A comparative clinical study. *Pak J Med Health Sci* 2022;**16**:305–7.

5. Al-Dboush R, Al-Zawawi E, El-Bialy T. Does short-term treatment with clear aligner therapy induce changes in muscular activity? *Evid Based Dent* 2023, DOI: 10.1038/s41432-023-00931-2.

6. Al-Moghrabi D, Johal A, O’Rourke N *et al.* Effects of fixed vs removable orthodontic retainers on stability and periodontal health: 4-year follow-up of a randomized controlled trial. *Am J Orthod Dentofacial Orthop* 2018;**154**:167-174.e1.

7. Al-Tayar B, Al-Somairi MAA, Alshoaibi LH *et al.* Impact of molar teeth distalization by clear aligners on temporomandibular joint: a three-dimensional study. *Prog Orthod* 2023;**24**, DOI: 10.1186/s40510-023-00474-3.

8. Al-Worafi NA, Zheng BW, Al-Warafi LA *et al.* Impact of molar teeth distalization by clear aligners on maxillary alveolar bone thickness and root resorption: a three-dimensional study. *BMC ORAL Health* 2024;**24**, DOI: 10.1186/s12903-024-03987-3.

9. Alajmi S, Shaban A, Al-Azemi R. Comparison of Short-Term Oral Impacts Experienced by Patients Treated with Invisalign or Conventional Fixed Orthodontic Appliances. *Med Princ Pr* 2020;**29**:382–8.

10. Alanazi AKA, Alqahtani AMN, Asiri AMA *et al.* Frequencies of Root Canal Treatment and Deep Caries in Clear Aligner compared To Conventional Orthodontic Treatment: A Retrospective Cohort Study. *Eur J Mol Clin Med* 2022;**9**:8218–23.

11. Alasiri MM, Almalki A, Alotaibi S *et al.* Association between Gingival Phenotype and Periodontal Disease Severity—A Comparative Longitudinal Study among Patients Undergoing Fixed Orthodontic Therapy and Invisalign Treatment. *Healthc Switz* 2024;**12**, DOI: 10.3390/healthcare12060656.

12. Alfawal AMH, Burhan AS, Mahmoud G *et al.* The impact of non-extraction orthodontic treatment on oral health-related quality of life: clear aligners versus fixed appliances - a randomized controlled trial. *Eur J Orthod* 2022;**44**:595–602.

13. Allahham DO, Kotsailidi EA, Barmak AB *et al.* Association between non-extraction clear aligner therapy and alveolar bone dehiscences and fenestrations in adults with mild-to-moderate crowding. *Am J Orthod Dentofac Orthop* 2023;**163**:22-32.e4.

14. Allareddy V, Nalliah R, Lee MK *et al.* Adverse clinical events reported during Invisalign treatment: Analysis of the MAUDE database. *Am J Orthod Dentofac Orthop* 2017;**152**:706–10.

15. Allereau B, Sabouni W. [Perception of pain in orthodontic treatment with thermoformed aligners]. *Orthod Fr* 2017;**88**:383–9.

16. AlSeraidi M, Hansa I, Dhaval F *et al.* The effect of vestibular, lingual, and aligner appliances on the quality of life of adult patients during the initial stages of orthodontic treatment. *Prog Orthod* 2021;**22**:3.

17. Alvarado-Lorenzo A, Antonio-Zancajo L, Baptista H *et al.* Comparative analysis of periodontal pain and quality of life in patients with fixed multibracket appliances and aligners (Invisalign®): longitudinal clinical study. *BMC ORAL Health* 2023;**23**, DOI: 10.1186/s12903-023-03565-z.

18. Aman C, Azevedo B, Bednar E *et al.* Apical root resorption during orthodontic treatment with clear aligners: A retrospective study using cone-beam computed tomography. *Am J Orthod Dentofacial Orthop* 2018;**153**:842–51.

19. Ana-Madalina R, Sebastian M, Aureliana C *et al.* Studies regarding salivary total antioxidant activity in different types of orthodontic treatement. *ROMANIAN Biotechnol Lett* 2019;**24**:332–9.

20. Angelopoulos GG, Kanarelis P, Vagdouti G *et al.* Oral impacts of aligners versus fixed self-ligating lingual orthodontic appliances. *Appl Sci Switz* 2021;**11**, DOI: 10.3390/app112110074.

21. Antonio-Zancajo L, Montero J, Albaladejo A *et al.* Pain and Oral-Health-Related Quality of Life in Orthodontic Patients During Initial Therapy with Conventional, Low-Friction, and Lingual Brackets and Aligners (Invisalign): A Prospective Clinical Study. *J Clin Med* 2020;**9**, DOI: 10.3390/jcm9072088.

22. Ashutosh W, Verghese Y, Mohammed A *et al.* A comparative evaluation of nickel-titanium wires and clear aligners in the management of mandibular incisor crowding. *J Orthod Sci* 2023;**12**:21.

23. Azaripour A, Weusmann J, Mahmoodi B *et al.* Braces versus Invisalign®: Gingival parameters and patients’ satisfaction during treatment: A cross-sectional study. *BMC Oral Health* 2015;**15**, DOI: 10.1186/s12903-015-0060-4.

24. Azeem M, Ul Hamid W. Incidence of white spot lesions during orthodontic clear aligner therapy. *J World Fed Orthod* 2017;**6**:127–30.

25. Ba-Hattab R, Almashraqi AA, Nasrawi YH *et al.* The effect of clear aligner and fixed orthodontic treatment on the development of pulp stones: a retrospective observational study. *Angle Orthod* 2025, DOI: 10.2319/091824-763.1.

26. Bai YX, Yang B, Dai Q *et al.* [Patients with anterior spaces caused by periodontal disease treated with aligner technique]. *Zhonghua Kou Qiang Yi Xue Za Zhi* 2009;**44**:421–4.

27. Balboni A, Lombardo EC, Balboni G *et al.* Vertical effects of distalization protocol with Clear aligners in Class II patients: a prospective study. *Minerva Dent Oral Sci* 2023;**72**:291–7.

28. Bargellini A, Graziano V, Cugliari G *et al.* Effects on Sleep Bruxism Activity of Three Different Oral Appliances: One Year Longitudinal Cohort Study. *Curr Drug Deliv* 2022, DOI: 10.2174/1567201819666220519123754.

29. Barreda GJ, Dzierewianko EA, Mazza V *et al.* Expansion treatment using Invisalign®: Periodontal health status and maxillary buccal bone changes. A clinical and tomographic evaluation. *Acta Odontol Latinoam* 2020;**33**:69–81.

30. Barreda GJ, Dzierewianko EA, Mazza V *et al.* Tratamiento de expansión con Invisalign®: estado de salud periodontal y cambios óseos maxilares. Evaluación clínica y tomográfica. *Acta Odontol Latinoam AOL* 2020;**33**:69–81.

31. Baseer MA, Almayah NA, Alqahtani KM *et al.* Oral Impacts Experienced by Orthodontic Patients Undergoing Fixed or Removable Appliances Therapy in Saudi Arabia: A Cross-Sectional Study. *Patient Prefer Adherence* 2021;**15**:2683–91.

32. Belgal P, Mhay S, Patel V *et al.* Adverse Events Related to Direct-To-Consumer Sequential Aligners-A Study of the MAUDE Database. *Dent J Basel* 2023;**11**, DOI: 10.3390/dj11070174.

33. Boggio A, Cozzani M. Indiscriminate alignment in cases with severe mandibular crowding: How to prevent and manage an everyday orthodontic problem. *J Clin Orthod JCO* 2024;**58**:29–36.

34. Bowman E, Bowman P, Weir T *et al.* Evaluation of the predicted vs. achieved occlusal outcomes with the Invisalign® appliance: A retrospective investigation of adult patients. *Int Orthod* 2023;**21**:100746.

35. Brascher AK, Zuran D, Feldmann RE *et al.* Patient survey on Invisalign<SUP>A®</SUP> treatment comparen the SmartTrack<SUP>A®</SUP> material to the previous aligner material. *J Orofac Orthop-FORTSCHRITTE KIEFERORTHOPADIE* 2016;**77**:432–8.

36. Brignardello-Petersen R. Moderate-to-high levels of satisfaction and no important concerns in patients who received treatment with clear orthodontic aligners. *J Am Dent Assoc* 2019;**150**:e20.

37. Caldas W, Bonin FA, Vianna CP *et al.* Influence of pain duration and severity on oral health-related quality of life and patient satisfaction during adult treatment with clear aligners. *Prog Orthod* 2024;**25**:18.

38. Caruso S, Nota A, Ehsani S *et al.* Impact of molar teeth distalization with clear aligners on occlusal vertical dimension: a retrospective study. *BMC Oral Health* 2019;**19**:182.

39. Caruso S, Nota A, Severino M *et al.* Mandibular advancement with clear aligners in the treatment of skeletal Class II. A retrospective controlled study. *Eur J Paediatr Dent* 2021;**22**:26–30.

40. Casteluci CEVF, Oltramari PVP, Conti PCR *et al.* Evaluation of pain intensity in patients treated with aligners and conventional fixed appliances: Randomized clinical trial. *Orthod Craniofac Res* 2021;**24**:268–76.

41. Castroflorio T, Bargellini A, Lucchese A *et al.* Effects of clear aligners on sleep bruxism: randomized controlled trial. *J Biol Regul Homeost Agents* 2018;**32**:21–9.

42. Chan V, Shroff B, Kravitz ND *et al.* Orthodontic pain with fixed appliances and clear aligners: A 6-month comparison. *Am J Orthod Dentofac Orthop* 2024;**166**:469–79.

43. Chauhan A, Mishra N, Patil D *et al.* Impact of Orthodontic Treatment on the Incidence of Dental Caries in Adolescents: A Prospective Cohort Study. *Cureus* 2024;**16**:e55898.

44. Chen Y, Li T, Xu Y *et al.* Oral health-related quality of life between Chinese and American orthodontic patients: A two-center cross-sectional study. *Chin J Plast Reconstr Surg* 2022;**4**:171–7.

45. Chen JP, Wen J, Huang L *et al.* Comparisons of maxillary incisor retraction effects for patients with first premolar extractions between Damon Q and Invisalign® A retrospective study. *Medicine (Baltimore)* 2022;**101**, DOI: 10.1097/MD.0000000000030919.

46. Ciavarella D, Fanelli C, Suriano C *et al.* Occlusal Plane Modification in Clear Aligners Treatment: Three Dimensional Retrospective Longitudinal Study. *Dent J Basel* 2022;**11**, DOI: 10.3390/dj11010008.

47. Clements KM, Bollen AM, Huang G *et al.* Activation time and material stiffness of sequential removable orthodontic appliances. Part 2: Dental improvements. *Am J Orthod Dentofac Orthop* 2003;**124**:502–8.

48. Cohen-Lévy J, Boulos C, Rompré P *et al.* Is the quality of occlusal contacts comparable after aligner and fixed orthodontic therapy? A non-randomized cohort comparison using computerized occlusal analysis during 6 months of retention. *Cranio* 2022:1–13.

49. Costello CJ, Kerr B, Weir T *et al.* The incidence and severity of root resorption following orthodontic treatment using clear aligners. *Australas Orthod J* 2020;**36**:130–7.

50. Dianiskova S, Bucci R, Solazzo L *et al.* Patient and Parental Satisfaction following Orthodontic Treatment with Clear Aligners and Elastodontic Appliances during Mixed Dentition: A Cross-Sectional Case-Control Study. *Appl Sci-BASEL* 2023;**13**, DOI: 10.3390/app13074074.

51. Donovan J, Millett D. Interproximal Reduction in Orthodontics: A Survey of Specialist Orthodontists and Patients. 2021.

52. Ertugrul BY, Veli İ. Evaluating the effects of orthodontic treatment with clear aligners and conventional brackets on mandibular condyle bone quality using fractal dimension analysis of panoramic radiographs. *J Stomatol Oral Maxillofac Surg* 2022;**123**:538–45.

53. Falconi V, Musella G, Borgia L *et al.* Evaluation of phonetic alterations in patients treated with f22 aligners. *Eur J Musculoskelet Dis* 2020;**9**:79–84.

54. Favero R, Libralato L, Balestro F *et al.* Edge level of aligners and periodontal health: a clinical perspective study in young patients. *Dent Press J Orthod Impr* 2023;**28**:e2321124–e2321124.

55. Feng QC, Jiang FL, Wang HX *et al.* Evaluation of modified clear Twin Block aligner in treating adolescents with skeletal class II malocclusion: A two-centre cephalometric study. *Orthod Craniofac Res* 2024;**27**:665–73.

56. Flores-Mir C, Brandelli J, Pacheco-Pereira C. Patient satisfaction and quality of life status after 2 treatment modalities: Invisalign and conventional fixed appliances. *Am J Orthod Dentofac Orthop* 2018;**154**:639–44.

57. Fountoulaki G, Thurzo A. Change in the Constricted Airway in Patients after Clear Aligner Treatment: A Retrospective Study. *Diagn Basel* 2022;**12**, DOI: 10.3390/diagnostics12092201.

58. Fowler B, Sameshima G. A Comparison of Root Resorption Between Invisalign Treatment and Contemporary Orthodontic Treatment. 2010:100.

59. Frenkel ES, Bollen A-M. Occlusal Contact Changes in Patients Treated with Clear Aligners. 2022:38.

60. Gandia C, Tabbaa S. The effect of Invisalign on the articulation of speech. 2015:59.

61. Garrett JA, Buschang PH. Effect of reducing the incremental distance of tooth movement per aligner while maintaining overall rate of movement on self-reported discomfort in Invisalign patients. 2012:71.

62. Gay G, Ravera S, Castroflorio T *et al.* Root resorption during orthodontic treatment with Invisalign®: a radiometric study. *Prog Orthod* 2017;**18**:12.

63. Gowda SVP, Chavan M, Kashyap R *et al.* Efficiency of hybrid aligners in surgical & non surgical cases undergoing orthodontic treatment: an original research. *Turk J Physiother Rehabil* 2021;**32**:10461â10465.

64. Gu Y, Tang X, Zhao Z. A comparative study on the effect of bracketless invisible orthodontics and fixed orthodontics on salivary flora. *Acta Medica Mediterr* 2022;**38**:3943–50.

65. Gupta S, Singh VK, Pandey S *et al.* 3D assessment of alveolar bone alterations in orthodontic movement among Indians. *Bioinformation* 2023;**19**:764–9.

66. Hakami Z. Comparison of sleep quality between clear aligner and fixed appliance orthodontic therapies. *J World Fed Orthod* 2023;**12**:245–50.

67. Han JY. A comparative study of combined periodontal and orthodontic treatment with fixed appliances and clear aligners in patients with periodontitis. *J Periodontal Implant Sci* 2015;**45**:193–204.

68. Han S, Zhao JW, Da HQ *et al.* Clinical study of digital clear aligner treatment of 33 adult periodontal disease patients with malocclusion. *Shanghai Kou Qiang Yi Xue Shanghai J Stomatol* 2020;**29**:386â389.

69. Harriott K, Myneni Venkatasatya S. The Periodontal Health Status of Invisalign Patients: A Clinical Study. 2017:71.

70. He X, Li X, Zhou X *et al.* Comparative evaluation of alveolar bone remodeling and root length changes in fixed appliances versus clear aligners: A retrospective cohort study on skeletal Class III malocclusion treatment. *J World Fed Orthod* 2025;**14**:12–9.

71. Heleiwa-Ferioli T, de la Cruz Vigo S. Association between bruxism and the use of aligners in orthodontics. *J Clin Exp Dent* 2024;**16**:e1247–55.

72. Hellak A, Schmidt N, Schauseil M *et al.* Influence of Invisalign treatment with interproximal enamel reduction (IER) on bone volume for adult crowding: a retrospective three-dimensional cone beam computed tomography study. *BMC Oral Health* 2016;**16**:83.

73. Huang J, Wang M, Tang X *et al.* Three-dimensional partitioning and quantification of orthodontic root resorption via automatic root extraction from cone-beam computed tomography. *BMC Oral Health* 2025;**25**.

74. Husain F, Warunek S, Gurav A *et al.* Influence of Invisalign precision bite ramp utilization on deep bite correction and root length in adults. *Angle Orthod* 2024;**94**:488–95.

75. Iglesias-Linares A, Sonnenberg B, Solano B *et al.* Orthodontically induced external apical root resorption in patients treated with fixed appliances vs removable aligners. *Angle Orthod* 2017;**87**:3–10.

76. Jaber ST, Hajeer MY, Burhan AS *et al.* The Effect of Treatment With Clear Aligners Versus Fixed Appliances on Oral Health-Related Quality of Life in Patients With Severe Crowding: A One-Year Follow-Up Randomized Controlled Clinical Trial. *Cureus* 2022;**14**:e25472.

77. Jan AL, Lin G-H. The Effect of Orthodontic Rotation and Proclination on Periodontium. 2023:45.

78. Jung B. Prevalence and Severity of apical Root Resorption during Orthodontic Treatment with clear Aligners and fixed Appliances: A Cone-Beam-Computed Tomography-Study. *INFORMATIONEN AUS Orthod Kieferorthop* 2021;**53**.

79. Jyotirmay, Singh SK, Adarsh K *et al.* Comparison of Apical Root Resorption in Patients Treated with Fixed Orthodontic Appliance and Clear Aligners: A Cone-beam Computed Tomography Study. *J Contemp Dent Pr* 2021;**22**:763–8.

80. Karkhanechi M, Chow D, Sipkin J *et al.* Periodontal status of adult patients treated with fixed buccal appliances and removable aligners over one year of active orthodontic therapy. *Angle Orthod* 2013;**83**:146–51.

81. Kim JE, Kim S, Kim DH. Comparison of oral health status, oral hygiene management behaviours and satisfaction of patients with fixed orthodontic appliance and clear aligner: A quasi-experimental design. *Int J Dent Hyg* 2024;**22**:939–48.

82. Klein BM, Araujo EA. A cephalometric study of adult mild Class II nonextraction treatment with the Invisalign system. 2013:96.

83. Knode V, Ludwig B, Retrouvey JM *et al.* Directly printed aligner therapy: A 12-month evaluation of application and effectiveness. *Am J Orthod Dentofacial Orthop* 2025;**167**:73–9.

84. Kobylyanskyy O, Schwarz L, Rausch MA *et al.* Changes in the alveolar bone morphology among different patterns of incisor inclination during the alignment phase in orthodontic treatment without premolar extraction. *Orthod Craniofac Res* 2024;**27**:645–55.

85. Krieger E, Drechsler T, Schmidtmann I *et al.* Apical root resorption during orthodontic treatment with aligners? A retrospective radiometric study. *Head Face Med* 2013;**9**:21.

86. Kuncio D, Maganzini A, Shelton C *et al.* Invisalign and traditional orthodontic treatment postretention outcomes compared using the American Board of Orthodontics objective grading system. *Angle Orthod* 2007;**77**:864–9.

87. Kuroda S, Iwata M, Tamamura N *et al.* Variables affecting orthodontic tooth movement with clear aligners. *Am J Orthod Dentofacial Orthop* 2014;**145**:S82–91.

88. Kurnaz S, Buyukcavus MH. Panoramic evaluation of external root resorption in mandibular molars during orthodontic treatment: a comparison between root-filled and vital teeth treated with fixed appliances or clear aligners. *BMC Oral Health* 2024;**24**:1152.

89. Kurtzner K, Al-Jewair T. A Longitudinal Study of the Effects of Invisalign Treatment on Upper Pharyngeal Airway Dimensions in Adults with Class II Malocclusion: A Cbct Pilot Study. 2019:64.

90. Levrini L, Mangano A, Montanari P *et al.* Periodontal health status in patients treated with the Invisalign® system and fixed orthodontic appliances: A 3 months clinical and microbiological evaluation. *Eur J Dent* 2015;**9**:404–10.

91. Levrini L, Bocchieri S, Mauceri F *et al.* Chewing Efficiency Test in Subjects with Clear Aligners. *Dent J* 2023;**11**, DOI: 10.3390/dj11030068.

92. Lin FO, Yao LJ, Bhikoo C *et al.* RETRACTED: Impact of fixed orthodontic appliance or clear-aligner on daily performance, in adult patients with moderate need for treatment(Retracted article. See vol.10,pg.2321,2016). *PATIENT Prefer ADHERENCE* 2016;**10**:1639–45.

93. Liu Z, Ouyang YQ, Lou YT *et al.* Orthodontically induced root resorption in endodontically treated and vital teeth: a cone beam computer tomographic study. *Prog Orthod* 2025;**26**, DOI: 10.1186/s40510-025-00553-7.

94. Liu F, Wang YH, Luopei D *et al.* Comparison of fixed braces and clear braces for malocclusion treatment. *BMC ORAL Health* 2024;**24**, DOI: 10.1186/s12903-024-04469-2.

95. Liu W, Shao JH, Li SF *et al.* Volumetric cone-beam computed tomography evaluation and risk factor analysis of external apical root resorption with clear aligner therapy. *ANGLE Orthod* 2021;**91**:597–603.

96. Lou TT, Tran J, Castroflorio T *et al.* Evaluation of masticatory muscle response to clear aligner therapy using ambulatory electromyographic recording. *Am J Orthod Dentofacial Orthop* 2021;**159**:E25–33.

97. Lz A, Mao J, Luo X. Analysis of oral health related life quality of fixed appliance and clear aligner adult patients at different orthodontic stages. *Chin J Orthod* 2022;**29**:205–8.

98. Madariaga ACP, Bucci R, Rongo R *et al.* Impact of Fixed Orthodontic Appliance and Clear Aligners on the Periodontal Health: A Prospective Clinical Study. *Dent J Basel* 2020;**8**, DOI: 10.3390/dj8010004.

99. Marcelino V, Baptista S, Marcelino S *et al.* Occlusal Changes with Clear Aligners and the Case Complexity Influence: A Longitudinal Cohort Clinical Study. *J Clin Med* 2023;**12**, DOI: 10.3390/jcm12103435.

100. McKenna S. Invisalign: technology or mythology? *J Mass Dent Soc* 2001;**50**:8–9.

101. Miguel YD, Alcântara PR, Toyofuku ACMM *et al.* Periodontal monitoring in orthodontic treatment with orthodontic aligners: proof of concept. *Rev Odontol UNESP* 2024;**53**, DOI: 10.1590/1807-2577.01624.

102. Miller KB, McGorray SP, Womack R *et al.* A comparison of treatment impacts between Invisalign aligner and fixed appliance therapy during the first week of treatment. *Am J Orthod Dentofac Orthop* 2007;**131**:302.e1-9.

103. Moon SJH, Allareddy V. Impact of COVID-19 Induced Closure of Orthodontic Clinic on Clinical Outcomes. 2023:60.

104. Mulla Issa F, Mulla Issa Z, Rabah A *et al.* Periodontal parameters in adult patients with clear aligners orthodontics treatment versus three other types of brackets: A cross-sectional study. *J Orthod Sci* 2020;**9**, DOI: 10.4103/jos.JOS_54_17.

105. Nedwed V, Miethke RR. Motivation, acceptance and problems of invisalign patients. *J Orofac Orthop* 2005;**66**:162–73.

106. Nicholson KC. A survey study comparing adult orthodontic patient quality of life between Invisalign and fixed appliances. 2011:67.

107. Nota A, Caruso S, Ehsani S *et al.* Short-Term Effect of Orthodontic Treatment with Clear Aligners on Pain and sEMG Activity of Masticatory Muscles. *Med Kaunas* 2021;**57**, DOI: 10.3390/medicina57020178.

108. Ozturk T, Hashimli ET, Coban G *et al.* Evaluation of white spot lesions after removable appliance treatment in the mixed dentition period with photodiagnostic method. *Clin Investig Orthod* 2025, DOI: 10.1080/27705781.2025.2480011.

109. Pacheco-Pereira C, Brandelli J, Flores-Mir C. Patient satisfaction and quality of life changes after Invisalign treatment. *Am J Orthod Dentofac Orthop* 2018;**153**:834–41.

110. Paes-Souza S de A, Garcia MAC, Souza VH *et al.* Response of masticatory muscles to treatment with orthodontic aligners: a preliminary prospective longitudinal study. *Dent Press J Orthod Impr* 2023;**28**:e232198–e232198.

111. Parrini S, Comba B, Rossini G *et al.* Postural changes in orthodontic patients treated with clear aligners: A rasterstereographic study. *J Electromyogr Kinesiol* 2018;**38**:44–8.

112. Patterson BD, Kim KB. Assessment of Predicted and Achieved Occlusion with Invisalign ®. 2018:74.

113. Pescheret C, Kusnoto B. The Effect of AcceleDent on Arch Alignment and Pain Level During Orthodontic Treatment with Invisalign. 2017:91.

114. Pittar N, Sicignano A, Bardini G *et al.* Effect of orthodontic appliances on masticatory muscle activity. *Semin Orthod* 2024;**30**:341–5.

115. Pittar N, Firth F, Bennani H *et al.* The effect of passive clear aligners on masticatory muscle activity in adults with different levels of oral parafunction. *J Oral Rehabil* 2023;**50**:1409–21.

116. Pogal-Sussman-Gandia CB, Tabbaa S, Al-Jewair T. Effects of Invisalign(®) treatment on speech articulation. *Int Orthod* 2019;**17**:513–8.

117. Risinger RC, Kasper FK, Cozad BE. A Three-Dimensional Comparative Analysis of Root Resorption After Clear Aligner Therapy. 2023:42.

118. Rouzi M, Jiang QS, Zhang HX *et al.* Characteristics of oral microbiota and oral health in the patients treated with clear aligners: a prospective study. *Clin Oral Investig* 2023, DOI: 10.1007/s00784-023-05281.

119. Rouzi M, Zhang XQ, Jiang QS *et al.* Impact of Clear Aligners on Oral Health and Oral Microbiome During Orthodontic Treatment. *Int Dent J* 2023;**73**:603–11.

120. Sabban HM, Al-Labban L, Baeshen N *et al.* Risk of root resorption between Invisalign and fixed orthodontic treatment: A retrospective study. *medRxiv* 2024, DOI: 10.1101/2024.12.05.24318570.

121. Saccomanno S, Laganà D, Mastrapasqua R *et al.* The relationship between TMJ symptoms and orthodontic treatments: a survey on 236 orthodontic patients. *J Biol Regul Homeost Agents* 2021;**35**:197–204.

122. Saccomanno S, Saran S, Salerno M *et al.* Clear aligners technique: patient’s expectations and perceptions. *J Biol Regul Homeost Agents* 2022;**36**:129–38.

123. Sánchez Gómez J. Aparatos removibles. *Conoc Odontol* 1996;**4**:22–22.

124. Santonocito S, Polizzi A. Oral Microbiota Changes during Orthodontic Treatment. *Front Biosci Elite Ed* 2022;**14**:19.

125. Sauer MK, Drechsler T, Peron PF *et al.* Aligner therapy in adolescents: first-year results on the impact of therapy on oral health-related quality of life and oral hygiene. *Clin Oral Investig* 2023;**27**:369–75.

126. Sharma R, Drummond R, Wiltshire W *et al.* Quality of life in an adolescent orthodontic population. *Angle Orthod* 2021;**91**:718–24.

127. Shokeen B, Viloria E, Duong E *et al.* The impact of fixed orthodontic appliances and clear aligners on the oral microbiome and the association with clinical parameters: A longitudinal comparative study. *Am J Orthod Dentofacial Orthop* 2022;**161**:E475–85.

128. Steele BP, Pandis N, Darendeliler MA *et al.* A comparative assessment of the dentoskeletal effects of clear aligners vs miniplate-supported posterior intrusion with fixed appliances in adult patients with anterior open bite. A multicenter, retrospective cohort study. *Am J Orthod Dentofac Orthop* 2022;**162**:214-228.e4.

129. Tepedino M, Colasante P, Staderini E *et al.* Short-term effect of orthodontic clear aligners on muscular activity and occlusal contacts: A cohort study. *Am J Orthod Dentofac Orthop* 2023;**164**:34–44.

130. Thavarajah R, Thennukonda RA. Analysis of adverse events with use of orthodontic sequential aligners as reported in the manufacturer and user facility device experience database. *Indian J Dent Res* 2015;**26**:582–7.

131. Toyokawa-Sperandio KC, Conti A, Fernandes TMF *et al.* External apical root resorption 6 months after initiation of orthodontic treatment: A randomized clinical trial comparing fixed appliances and orthodontic aligners. *KOREAN J Orthod* 2021;**51**:329–36.

132. Tran J, Lou TT, Nebiolo B *et al.* Impact of clear aligner therapy on tooth pain and masticatory muscle soreness. *J ORAL Rehabil* 2020;**47**:1521–9.

133. Tunca Y, Kaya Y, Tunca M *et al.* Comparison of anxiety, pain, and quality of life in individuals with mild or moderate malocclusion between conventional fixed orthodontic treatment versus Invisalign: a randomised clinical trial. *BMC Oral Health* 2024;**24**:576.

134. Wadia R. Clear aligner therapy - OGE & IPR. *Br Dent J* 2023;**234**:100.

135. Wang DR, Firth F, Bennani F *et al.* Immediate effect of clear aligners and fixed appliances on perioral soft tissues and speech. *Orthod Craniofac Res* 2023;**26**:425–32.

136. Xu L, Li HS, Mei L *et al.* Aligner treatment: patient experience and influencing factors. *Australas Orthod J* 2022;**38**:88–95.

137. Xu L, Mei L, Lu RQ *et al.* Predicting patient experience of Invisalign treatment: An analysis using artificial neural network. *KOREAN J Orthod* 2022;**52**:268–77.

138. Yi JR, Xiao JI, Li Y *et al.* External apical root resorption in non-extraction cases after clear aligner therapy or fixed orthodontic treatment. *J Dent Sci* 2018;**13**:48–53.

139. Zamora-Martínez N, Paredes-Gallardo V, García-Sanz V *et al.* Comparative Study of Oral Health-Related Quality of Life (OHRQL) between Different Types of Orthodontic Treatment. *Med Kaunas* 2021;**57**, DOI: 10.3390/medicina57070683.

140. Zhang YBH, Wang X, Wang JH *et al.* IPR treatment and attachments design in clear aligner therapy and risk of open gingival embrasures in adults. *Prog Orthod* 2023;**24**, DOI: 10.1186/s40510-022-00452-1.

141. Zhao R, Huang R, Long H *et al.* The dynamics of the oral microbiome and oral health among patients receiving clear aligner orthodontic treatment. *Oral Dis* 2020;**26**:473–83.

142. Zheng JJ, Zhang YY, Wu QY *et al.* Three-dimensional spatial analysis of the temporomandibular joint in adult patients with Class II division 2 malocclusion before and after orthodontic treatment: a retrospective study. *BMC ORAL Health* 2023;**23**, DOI: 10.1186/s12903-023-03210-9.

143. Zybutz T, Drummond R, Lekic M *et al.* Investigation and comparison of patient experiences with removable functional appliances. *Angle Orthod* 2021;**91**:490–5.
