# Supplementary material for: Adverse effects of removable orthodontic aligners: A systematic review with single-arm meta-analysis
Source: PLoS One. 2026 Jul 20;21(7):e0350741. doi: 10.1371/journal.pone.0350741 (PMC13384317; doi:10.1371/journal.pone.0350741)
Supplement: S8 — (DOCX) [file pone.0350741.s008.docx]

**R Scripts**

***Pain 24 hours***

install.packages("meta")

install.packages("readxl")

install.packages("writexl")

library(meta)

library(readxl)

library(writexl)

pain_24h <- read_excel("AdverseEffect_database/Pain_24h.xlsx")

print(names(pain_24h))

m1 <- metamean(

n = n,

mean = mean,

sd = sd,

studlab = id_study,

data = pain_24h,

sm = "MRAW",

method.tau = "REML",

method.random.ci = "HK",

random = TRUE,

common = FALSE

)

summary(m1)

forest(

m1,

leftcols = c("studlab", "study_design", "tool", "n", "mean", "sd"),

leftlabs = c("Study", "Study Design", "Tool", "Sample", "Mean", "SD"),

rightlabs = c("Mean [95% CI]"),

print.I2 = TRUE,

print.tau2 = TRUE,

print.pval.Q = TRUE,

col.diamond = "black",

col.diamond.lines = "black",

xlab = "Pain - after 24 hours (mean, 95% CI)",

xlim = c(0, 10),

random = TRUE,

common = FALSE

)

funnel(

m1,

xlab = "Effect size",

ylab = "Standard Error",

main = "Funnel plot - Pain after 24 hours"

)

metabias(m1, method.bias = "Egger")

unique(pain_24h$id_study)

pain_24h_no_rucker <- subset(pain_24h, id_study != "Rucker, 2012 (United States)")

unique(pain_24h_no_rucker$id_study)

m1_no_rucker <- metamean(

n = n,

mean = mean,

sd = sd,

studlab = id_study,

data = pain_24h_no_rucker,

sm = "MRAW",

method.tau = "REML",

method.random.ci = "HK",

random = TRUE,

common = FALSE

)

summary(m1_no_rucker)

forest(

m1_no_rucker,

leftcols = c("studlab", "study_design", "tool", "n", "mean", "sd"),

leftlabs = c("Study", "Study Design", "Tool", "Sample", "Mean", "SD"),

rightlabs = c("Mean [95% CI]"),

print.I2 = TRUE,

print.tau2 = TRUE,

print.pval.Q = TRUE,

col.diamond = "black",

col.diamond.lines = "black",

xlab = "Pain - after 24 hours without Rucker, 2012 (mean, 95% CI)",

xlim = c(0, 10),

random = TRUE,

common = FALSE

)

comparison_results <- data.frame(

Analysis = c("Primary analysis", "Sensitivity analysis without Rucker"),

k = c(m1$k, m1_no_rucker$k),

N_total = c(sum(pain_24h$n, na.rm = TRUE),

sum(pain_24h_no_rucker$n, na.rm = TRUE)),

Pooled_mean = c(m1$TE.random, m1_no_rucker$TE.random),

Lower_CI = c(m1$lower.random, m1_no_rucker$lower.random),

Upper_CI = c(m1$upper.random, m1_no_rucker$upper.random),

I2 = c(m1$I2, m1_no_rucker$I2),

tau2 = c(m1$tau^2, m1_no_rucker$tau^2),

Q_pvalue = c(m1$pval.Q, m1_no_rucker$pval.Q)

)

print(comparison_results)

write_xlsx(comparison_results, "comparison_pain_24h_with_without_Rucker.xlsx")

inf_24h <- metainf(m1)

print(inf_24h)

forest(

inf_24h,

xlab = "Leave-one-out analysis: pain after 24 hours"

)

cat("\n============================\n")

cat("PRIMARY ANALYSIS\n")

cat("============================\n")

cat("Pooled mean:", round(m1$TE.random, 2), "\n")

cat("95% CI:", round(m1$lower.random, 2), "to", round(m1$upper.random, 2), "\n")

cat("I2:", round(m1$I2, 1), "%\n")

cat("tau2:", round(m1$tau^2, 4), "\n")

cat("p-value Q:", signif(m1$pval.Q, 3), "\n")

cat("\n============================\n")

cat("SENSITIVITY ANALYSIS WITHOUT RUCKER\n")

cat("============================\n")

cat("Pooled mean:", round(m1_no_rucker$TE.random, 2), "\n")

cat("95% CI:", round(m1_no_rucker$lower.random, 2), "to", round(m1_no_rucker$upper.random, 2), "\n")

cat("I2:", round(m1_no_rucker$I2, 1), "%\n")

cat("tau2:", round(m1_no_rucker$tau^2, 4), "\n")

cat("p-value Q:", signif(m1_no_rucker$pval.Q, 3), "\n")

cat("\n============================\n")

cat("DIFFERENCE IN POOLED MEAN\n")

cat("============================\n")

cat("Difference:", round(m1$TE.random - m1_no_rucker$TE.random, 2), "\n")

***MA Pain 3 days***

install.packages("meta")

library(meta)

library(readxl)

pain_3d <- read_excel("AdverseEffect_database/pain_3d.xlsx")

m2 <- metamean(

n = n,

mean = mean,

sd = sd,

studlab = paste(study),

data = pain_3d,

sm = "MLN",

method.tau = "REML",

method.random.ci = TRUE

)

forest(

m2,

leftcols = c("studlab", "study_design", "tool", "n", "mean", "sd"),

leftlabs = c("Study", "Study Design", "Tool", "Sample", "Mean", "SD"),

rightlabs = c("Mean [95% CI]"),

print.I2 = TRUE,

print.tau2 = TRUE,

print.pval.Q = TRUE,

col.diamond = "black",

col.diamond.lines = "black",

xlab = "Pain - after 3 days (mean, 95% CI)",

xlim = c(0, 10),

random = TRUE,

common = FALSE

)

***MA Pain 1 week***

install.packages("meta")

library(meta)

library(readxl)

pain_1w <- read_excel("AdverseEffect_database/pain_1w.xlsx")

m3 <- metamean(

n = n,

mean = mean,

sd = sd,

studlab = paste(study),

data = pain_1w,

sm = "MLN",

method.tau = "REML",

method.random.ci = TRUE

)

forest(

m3,

leftcols = c("studlab", "study_design", "tool", "n", "mean", "sd"),

leftlabs = c("Study", "Study Design", "Tool", "Sample", "Mean", "SD"),

rightlabs = c("Mean [95% CI]"),

print.I2 = TRUE,

print.tau2 = TRUE,

print.pval.Q = TRUE,

col.diamond = "black",

col.diamond.lines = "black",

xlab = "Pain - after 1 week (mean, 95% CI)",

xlim = c(0, 10), # <--- aqui define o eixo de 0 a 10

random = TRUE, # mostra o efeito randômico (default)

common = FALSE # oculta o efeito fixo

)

***MA Root Resorption 18 months***

install.packages("readxl")

install.packages("meta")

library(readxl)

library(meta)

data <- read_excel("AdverseEffect_database/RootResorption_18m.xlsx")

m1 <- metamean(

n = data$sample,

mean = data$change,

sd = data$sd,

studlab = paste(data$study),

data = data,

sm = "MRAW",

method.tau = "REML"

)

forest(

m1,

leftcols = c("studlab", "study_design", "tool", "n", "change", "sd"),

leftlabs = c("Study", "Study Design", "Tool", "Sample", "MD", "SD"),

rightlabs = c("Mean Difference [95% CI]"),

print.I2 = TRUE,

print.tau2 = TRUE,

print.pval.Q = TRUE,

col.diamond = "black",

col.diamond.lines = "black",

xlab = "Root Resorption (mm) - after 18 months (mean, 95% CI)",

xlim = c(-6, 2) ,

random = TRUE,

common = FALSE

)

***MA Root Resorption Total***

install.packages("readxl")

install.packages("meta")

library(readxl)

library(meta)

data <- read_excel("AdverseEffect_database/RootResorption_Total.xlsx")

m1 <- metamean(

n = data$sample,

mean = data$change,

sd = data$sd,

studlab = paste(data$study),

subgroup = data$follow_up,

data = data,

sm = "MRAW",

method.tau = "REML"

)

forest(

m1, # seu objeto meta-análise

leftcols = c("studlab", "study_design", "tool", "site", "follow_up", "n", "change", "sd"),

leftlabs = c("Study", "Study Design", "Tool", "Site", "Follow up", "Sample", "MD", "SD"),

rightlabs = c("Mean Difference [95% CI]"),

print.I2 = TRUE,

print.tau2 = TRUE,

print.pval.Q = TRUE,

col.diamond = "black",

col.diamond.lines = "black",

xlab = "Root Resorption (mm)- End of treatment (mean, 95% CI)",

xlim = c(-6, 2),

random = TRUE,

common = FALSE

)

***MA Plaque Index***

# install.packages("readxl"); install.packages("meta")

library(readxl)

library(meta)

dat <- read_excel("AdverseEffect_database/PI_change_3studies_from_medians.xlsx", sheet = "change_input_r0.5")

dat_primary <- subset(dat, !grepl("RMS", study))

m_chg_2 <- metamean(

n = dat_primary$n,

mean = dat_primary$mean_change,

sd = dat_primary$sd_change,

studlab = dat_primary$study,

data = dat_primary,

sm = "MRAW",

method.tau = "REML",

method.random.ci = TRUE

)

summary(m_chg_2)

forest(

m_chg_2,

leftcols = c("studlab", "n", "mean", "sd"),

leftlabs = c("Study", "N", "Δ Mean", "SD(Δ)"),

rightlabs = c("Δ Mean [95% CI]"),

print.I2 = TRUE,

print.tau2 = TRUE,

print.pval.Q = TRUE,

col.diamond = "black",

col.diamond.lines = "black",

xlab = "Δ Plaque Index (3m − baseline), Silness–Löe (0–3)",

xlim = c(-1.5, 1.5),

random = TRUE,

common = FALSE

)
